# Palmitic acid-activated GPRs/KLF7/CCL2 pathway is involved in the crosstalk between bone marrow adipocytes and prostate cancer

**DOI:** 10.1186/s12885-024-11826-5

**Published:** 2024-01-15

**Authors:** Jingzhou Wang, Jie Liu, Chenggang Yuan, Bingqi Yang, Huai Pang, Keru Chen, Jiale Feng, Yuchun Deng, Xueting Zhang, Wei Li, Cuizhe Wang, Jianxin Xie, Jun Zhang

**Affiliations:** 1 Shihezi University School of Medicine, Bei-Er-Lu, Shihezi, 832000 Xinjiang China; 2 Laboratory of Xinjiang Endemic and Ethic Diseases, Shihezi University, Shihezi, 832000 Xinjiang China

**Keywords:** Palmitic acid, Bone marrow adipocytes, GPRs/KLF7/CCL2, Prostate cancer

## Abstract

**Background:**

Obesity-induced abnormal bone marrow microenvironment is one of the important risk element for bone metastasis in prostate cancer (PCa). The present study aimed to determine whether obesity-induced elevation in palmitic acid (PA), which is the most abundant of the free fatty acids (FFAs), increased CCL2 via the GPRs/KLF7 pathway in bone marrow adipocytes (BMA) to facilitate PCa growth and metastasis.

**Methods:**

We constructed a bone-tumor bearing mouse model with obesity through high-fat diet, and observed the tumor formation ability of PCa cells. In vitro, observe the effect of PA on the expression level of CCL2 in BMA through GPRs/KLF7 signaling pathway. After co-culture of BMA and PCa cells, CCK8 assay and transwell experiment were used to detect the changes in biological behavior of PCa cells stimulated by BMA.

**Results:**

The BMA distribution in the bone marrow cavity of BALB/c nude mice fed with the high-fat diet (HFD) was evidently higher than that in the mice fed with the normal diet (ND). Moreover, HFD-induced obesity promoted KLF7/CCL2 expression in BMA and PCa cell growth in the bone marrow cavity of the mice. In the vitro experiment, a conditioned medium with increased CCL2 obtained from the BMA cultured with PA (CM-BMA-PA) was used for culturing the PCa cell lines, which evidently enhanced the proliferation, invasion, and migration ability. KLF7 significantly increased the CCL2 expression and secretion levels in BMA by targeting the promoter region of the CCL2 gene. In addition, GPR40/120 engaged in the PA-induced high KLF7/CCL2 levels in BMA to facilitate the malignant progression of PC-3 cells.

**Conclusions:**

PA-activated GPRs/KLF7/CCL2 pathway in BMA facilitates prostate cancer growth and metastasis.

**Supplementary Information:**

The online version contains supplementary material available at 10.1186/s12885-024-11826-5.

## Background

Prostate cancer (PCa) is one of the most frequently diagnosed malignant tumors in elderly men [1]. The incidence of PCa has increased over time, particularly in the developing nations of Asia, Northern Europe, and Western Europe [2–4]. According to the clinical data, 75%–80% of PCa patients develop bone metastasis. Among patients who develop advanced PCa, 80% are affected by bone metastasis, with a sharp drop in survival rate [5]. Therefore, bone tissue is considered the main site of PCa metastasis [6–8]. In addition, about 90% of patients who died of PCa had bone metastasis, which means that the degree of bone metastasis may predict the prognosis of PCa [9].

The main explanation accepted currently for bone metastasis in PCa is the “seed and soil” theory, according to which the unique bone marrow microenvironment offers “fertile soil” for PCa colonization and growth [10, 11]. Numerous epidemiological studies have demonstrated that obesity-induced abnormal bone marrow microenvironment is the key risk element for bone metastasis in PCa [12, 13]. Specifically, HFD-induced obesity could promote the formation of bone marrow adipocytes (BMA) and inhibit the formation of osteoblasts in the bone marrow [14]. The increased BMA in the obese state serves as the energy source for metastatic cancer cells, while also releasing a few adipokines to facilitate PCa cell proliferation and metastasis [12, 15].

In the bone marrow microenvironment, overexpression of the C–C chemokine ligand 2 (CCL2) secreted by BMA, is the main contributor to tumor growth and metastasis in the obese state [16, 17]. It is reported that adipocyte-derived CCL2 contributes to PCa cell survival, proliferation, and metastasis via the CCR2 signaling pathway. A previous study by our research group revealed that the CCL2 secretion levels of 3T3-L1 adipocytes are significantly increased after stimulation with high concentrations of palmitic acid (PA) [18, 19]. However, whether PA could be one of the important inducements for BMA to secrete huge amounts of CCL2 for promoting the colonization and growth of PCa cells in the bone under an obese state remains to be elucidated so far.

The G protein-coupled receptors (GPRs) are a group of fatty acid receptors, including GPR40, GPR41, GPR43, GPR84, and GPR120, which participate in the regulation of downstream signaling pathways in various types of cancer. Among the different GPRs, GPR40 and GPR120 act as the receptors of PA [20]. Therefore, it is important to determine whether GPR40 and GPR120 affect the PA-mediated elevated levels of CCL2 in BMA.

Krüppel-like factor 7 (KLF7) is a member of the KLF family. KLF7 regulates the expression and secretion of several genes associated with fat metabolism and several inflammatory factors in mature adipocytes [21, 22]. Recent studies conducted in our laboratory revealed a significant increase in the expression levels of KLF7 and CCL2 in 3T3-L1 adipocytes under stimulation with high concentrations of PA. In addition, KLF7 upregulation significantly increases the CCL2 expression [19, 23].

Therefore, the present study involved the establishment of a nude mice model of obesity, which was established and injected with PC-3 cells, and a co-culture model of BMA and PCa cells in vitro to explore whether high concentrations of PA could activate the GPRs/KLF7/CCL2 pathway in BMA, thereby leading to prostate cancer growth and metastasis of the bone marrow. The present study provides a novel theoretical and experimental foundation for future research on the prevention and treatment of PCa bone metastasis clinically.

## Methods

### Animals

Twelve male BALB/c nude mice aged four weeks were procured from Beijing Vital River Laboratory Animal Technology Co. Ltd. The animals were maintained at the experimental animal center of the Xinjiang Medical University. After one week of adaptive feeding, the mice were fed with a high-fat diet (HFD, *n* = 8, 60% fat kcal; Jiangsu Madison Biomedical Co. Ltd., Cat. No: MD12033) or a normal diet (ND, *n* = 4, 10% fat kcal; Jiangsu Madison biomedical Co. Ltd. Cat. No: MD12031). Refers to Jinlu Dai et al [24], after five weeks of HFD feeding, PC-3-Luc cells (PC-3 cells stably transfected with Luci elements; Suzhou Jima gene Co. Ltd.) were injected at a density of 5 × 10^5^  into the femur of the left leg of each mouse. The femoral wounds were sealed using bone wax, following which the mice were continued with the HFD feeding for three weeks.

In the eighth week, the mice were anesthetized using phenobarbital, followed by the administration of the intraperitoneal injection of the D-Luc substrate enzyme. The small animal imaging technology was adopted to observe the PCa formation in the bone marrow within 30 min. Subsequently, blood samples were collected from the retroorbital vein and subjected to centrifugation at 2504 g for 10 min. The obtained serum was freeze-stored at –80℃. The left femur was fixed in 4% tissue cell fixation solution for 24 h, soaked in the neutral EDTA decalcification solution for a day, dehydrated using 70% ethanol for 24 h, and then with 95% ethanol for another day, and finally, embedded in paraffin. The bone marrow in the femur of the right leg was washed using 1 mL PBS and then freeze-stored at –80℃. All mice experiments were conducted with the approval of the Medical Ethics Committee of the First Affiliated Hospital, Shihezi University School of Medicine (reference number: A2017–115–01).

### Hematoxylin and Eosin (H&E) staining

The paraffin-embedded tissue slides were placed in an oven at 60℃ for 30 min. Subsequently, the slides were placed successively into xylene, 100% alcohol, 90% alcohol, 80% alcohol, and 70% alcohol. Next, the slides were washed three times in water (30 s/time) and then subjected to hematoxylin dying for 5 min. After rinsing five times in water, the slides were placed in 1% hydrochloric acid for 5 s. After three more rounds of washing with water, the slides were subjected to staining with eosin for 1 min. Again, the slides were washed three times with water and then placed successively in 70%, 80%, 90%, and absolute ethanol and xylene of different purity levels for dehydration. Finally, the slides were sealed with neutral gum. According to existing references, the bone marrow cavity was photographed under the Olympus microscope at 200×. Use cellSens Standard software to measure the BMA which greater than 50μm [25].

### Immunohistochemistry (IHC)

The paraffin-embedded tissue slides were placed in an oven at 60℃ for 30 min. Subsequently, the slides were placed successively in xylene, 100% alcohol, 100% alcohol, 95% alcohol, 90% alcohol, 80% alcohol, and 70% alcohol. After three washes with clean water (30 s/time), the slices were placed inside the repair box containing EDTA solution (pH 8.0) for antigen repair inside a high-pressure cooker for 8 min. Afterward, the slides were cooled to room temperature, rinsed three times in water, and incubated overnight with anti-KLF7 antibodies (1:200; Abcam, Cambridge, USA) or anti-CCL2 antibodies (1:100; Abcam, Cambridge, USA) at 4℃. Next, the slides were incubated with the anti-mouse or anti-rabbit HRP secondary antibody (DAKO, Glostrup, Denmark) at 37℃ for 30 min and then visualized with 3.3’-diaminobenzidine (DAB). The IHC scores were reviewed by two pathologists blinded to the study design, at the First Affiliated Hospital, Shihezi University School of Medicine. The scoring criteria were as follows: the proportion of the positive cells (0%–5%, 0; 6%–25%, 1; 26%–50%, 2; 51%–75%, 3; 76%–100%, 4) and positive staining intensity (negative, 0; canary yellow, 1; brownish yellow, 2; brown, 3). The final score was calculated by multiplying the proportion of the positive cells by the positive staining intensity.

### Biochemical indicator test

In this study, FFA, TG, TC, HDL-C, LDL-C, and glucose levels were all performed according to the procedure in the kit (A042-2, A110-1–1, A111-1–1, A112-1–1, A113-1–1, and F006-1–1, Nanjing Jiancheng Bioengineering Institute, China).CCL2 were performed according to the procedure in the ELISA kit(human: KE00091, mouse: KE10006, Proteintech Group, USA), the same as PA in serum (FS-0501, Shanghai Fusheng Bioengineering Institute, China).

### Cell lines and culture conditions

Adult bone marrow mesenchymal stem cells (hMSC-BM) were obtained from Cyagen (Guangzhou) Biotechnology Co. Ltd. Human PCa cells [PC-3 (CVCL_0035) and 22RV1 (CVCL_1045) cell lines] were obtained from the cell bank of the typical culture treasure committee at the Chinese Academy of Sciences.

Culture and differentiation of hMSC-BM: The hMSC-BM cells were cultured in DMEM supplemented with 10% fetal bovine serum. The differentiation of hMSC-BM was induced using the adipogenic-differentiation medium (Cyagen Biosciences, Santa Clara, USA). Oil red O stain (Cyagen Biosciences) was used for detecting the adipogenic differentiation in cells.

Culture of PC-3 cells and 22RV1 cells: PC-3 cells were cultured in the F12 medium supplemented with 10% fetal bovine serum and 1% penicillin–streptomycin. The 22RV1 cells were cultured in RPMI 1640 supplemented with 10% fetal bovine serum and 1% penicillin–streptomycin.

Culture of 293 T cells: The 293 T cells were cultured in 4.5 g/L of D-Glucose DMEM medium supplemented with 10% fetal bovine serum and 1% penicillin–streptomycin.

Co-culture of hMSC-BM and PCa cells: Collect BMA cell supernatants from different groups for processing PC-3 cells and 22RV1 cells. In the proliferation assays, the PC-3 and 22RV1 cells were inoculated at a density of 8 × 10^3^ cells/well and 2 × 10^4^ cells/well, respectively, in the wells of 96-well plates. After 24 h, all cells adhered to the wall and grew. Different groups of CM-BMA (100 µ L/well) were used to co-culture PCa cells. In the invasion and migration assays, PC-3 (8 × 10^4^ cells) and 22RV1 (2 × 10^5^ cells) were applied to the upper Transwell chamber(3422, Corning Costar, USA), while different CM-BMA were placed in the lower chamber(500 µL/well).

The culture medium that was used for the adipogenic differentiation of hMSC-BM was harvested and diluted using the F12/RPMI 1640 medium containing 10% FBS. The resulting medium was then used for culturing the PC-3 cells or 22RV1 cells.

The above method has been used for a long time in our laboratory to cultivate PC-3, 22RV1, and hMSC-BM cells. In 2020, this method was used for the research that was subsequently published in the Cancer Management and Research [26] and Cancer Science [27] journals.

In the last three years, short tandem repeat profiling was used for the verification of human cell lines. The experiments were conducted in mycoplasma-free cells.

### Reagents and materials

The preparation of 40 mM PA solution: PA (Sigma-Aldrich, St. Louis, USA, 0.0614 g) was injected into 3 mL of 0.1 mol/L NaOH solution, and the mixture was placed in a full saponification water bath at 75℃ for half an hour until the PA particles were completely dissolved and the liquid had turned colorless and apparent. Afterward, 3 mL of BSA (40%, free of fatty acid) solution was injected into the liquid followed by thorough mixing. Next, 100 mM AH7614 solution was prepared by dissolving AH7614 (TOCRIS, England, 10 mg) in 285 µL of DMSO. The 100 mM GW1100 solution was prepared by dissolving 5 mg of GW1100 (MCE, America, 5 mg) in 960 µL of DMSO.

Turkish galls (Quercus infectoria Oliv.) is a finished product donated by Professor Han Bo from the School of Pharmacy, Shihezi University. In this study, dry powdered Turkish galls were dissolved in sterile PBS at the required concentration and used for subsequent experiments. The insect galls was identified by Professor Bo Han. Voucher specimens (NO. 20,160,305) were preserved in the School of Pharmacy, Shihezi University.

### Quantitative Real-Time PCR (qRT-PCR)

The total RNA was extracted using TRIzol (Invitrogen, CA, USA). First-strand cDNA was produced based on the RNA template (1 µg) using the RevertAid First Strand cDNA Synthesis Kit (ThermoScientific, CA, USA). Reverse transcription was performed at 42℃ for 60 min and then at 70℃ for a quarter. PCR amplification was conducted using the qRT-PCR instrument (QIAGEN, Hilden, Germany) at the following program settings: 95℃ for 3–5 min, followed by 40–45 cycles of 95℃ for 10 s, 50–60℃ for 30 s, and 72℃ for 40 s. GAPDH or β-actin was used as the internal control. The data were collected as CT values, which were calculated using the 2^−ΔCt^ method or the 2^−ΔΔCt^ method. The primer sequences are provided in Supplementary Table 1.

### Western blot

Cell lysis was performed using the RIPA (including 1% PMSF) protein extraction solution (Solarbio, Beijing, China). The protein concentrations were measured using the BCA protein concentration assay kit (Solarbio, Beijing, China). SDS–polyacrylamide gel electrophoresis (PAGE) was conducted to separate the extracted proteins with different molecular weights. The target proteins were immediately transferred to nitrocellulose membranes, which were then incubated overnight at 4℃ with antibodies against β-actin (42 kDa; ZSGB-BIO, Beijing, China), β-Tubulin (55 kDa; ZSGB-BIO, Beijing, China), GPR40 (40 kDa; Abcam, Cambridge, USA), GPR120 (40 kDa; Abcam, Cambridge, USA), KLF7 (25 kDa; Abcam, Cambridge, USA), CCL2 (40 kDa; Abcam, Cambridge, USA), CCR2 (35 kDa; San Ying Biotechnology, Wuhan, China), Ki67 (384 kDa; Abcam, Cambridge, USA), and MMP2 (72 kDa; Abcam, Cambridge, USA). Afterward, the membranes were incubated with the secondary antibody at room temperature for 2 h. It needs to be explained that, in order to reduce the mutual interference of polyclonal antibody incubation among multiple antibodies, we have tailored according to MW markers in this study, and each membrane containing different target proteins is incubated separately with corresponding antibodies.The protein bands were detected based on chemiluminescence (ThermoScientific, Waltham, America).

### The Luciferase reporter gene experiment

In the present study, the Eukaryotic Promotor Database software was employed to search the gene sequence in the CCL2 promoter region. Jaspar database was used for predicting the binding site in the KLF7 and CCL2 promoter regions. The human CCL2 promoter region core segment luciferase plasmid (2001 bp) and the truncated luciferase reporter gene plasmids (1501 bp, 1001 bp, and 501 bp) (GenePharma), along with every luciferase reporter gene plasmid, KLF7 overexpression plasmid, and Renilla fluorescence plasmid were co-transfected into the 293 T cells. The equipment used was a full-wavelength scanning multi-functional microplate reader (BioTeK). The evaluation kit used was the Promega Dual-Glo Luciferase Assay System E2920. After sample addition, the following steps were performed in the order described: Dual Glo Luciferase Assay Reagent was added to the plate, which was then incubated at 20–25℃ for 10 min–2 h, followed by the firefly luminescence measurement; the Dual-Glo Stop and Glo Reagent was added to the plate, which was then incubated at 20–25℃ for 10 min, followed by the Renilla luminescence measurement. In order to determine the firefly ratio, Renilla luminescence per well was determined, followed by the normalization of the sample well ratio with respect to the control well ratio (or ratio of a series of control wells).

### Cell proliferation analysis

The PC-3 and 22RV1 cells were inoculated at a density of 8 × 10^3^ cells/well and 2 × 10^4^ cells/well, respectively, in the respective media (200 µL/well) in the wells of 96-well plates. The cells were then incubated at 37℃ for three different time durations of 24 h, 48 h, and 72 h. Finally, 10 µL of the Cell Counting Kit-8 (CCK-8) (Model 680; Bio-Rad Laboratories, Inc., Hercules, CA, USA) solution was added to each well, and the plates were incubated at 37℃ for 2 h. The optical density (OD) value for each well was measured at 450 nm inside a microplate reader (Model 680; Bio-Rad Laboratories Inc., Hercules, CA, USA).

### Cell invasion assay and migration assay

The cell migration assays were conducted using the Boyden Chamber Transwell system (3422, Corning Costar, USA). In the invasion assays, cells invading via the Matrigel-coated membrane (356234, Solarbio, Beijing, China) were examined. PC-3 (8 × 10^4^ cells) and 22RV1 (2 × 10^5^ cells) were applied to the upper Transwell chamber, while different media were placed in the lower chamber. Subsequently, 500 µL of 0.01% crystal violet dye solution was added to stain the cells at room temperature for 20 min. The cells that had not invaded or migrated were wiped off using cotton swabs. The bottom of each chamber was divided into nine grids. The number of cells in each grid was counted by two different researchers who were blinded to the study. The mean values were calculated for the statistical analysis.

### Elisa assay

The contents of the cytokine CCL2 in cells were detected using the ELISA kit (Xitang Biotechnology, Shanghai, China).

### Statistical analysis

SPSS 17.0 software was employed to conduct the unpaired t-test, one-way ANOVA, and the nonparametric rank-sum test.* P* < 0.05 was considered the threshold of statistical significance.

## Results

### Obesity promoted tumor growth in the bone marrow cavity

**Fig. 1:**
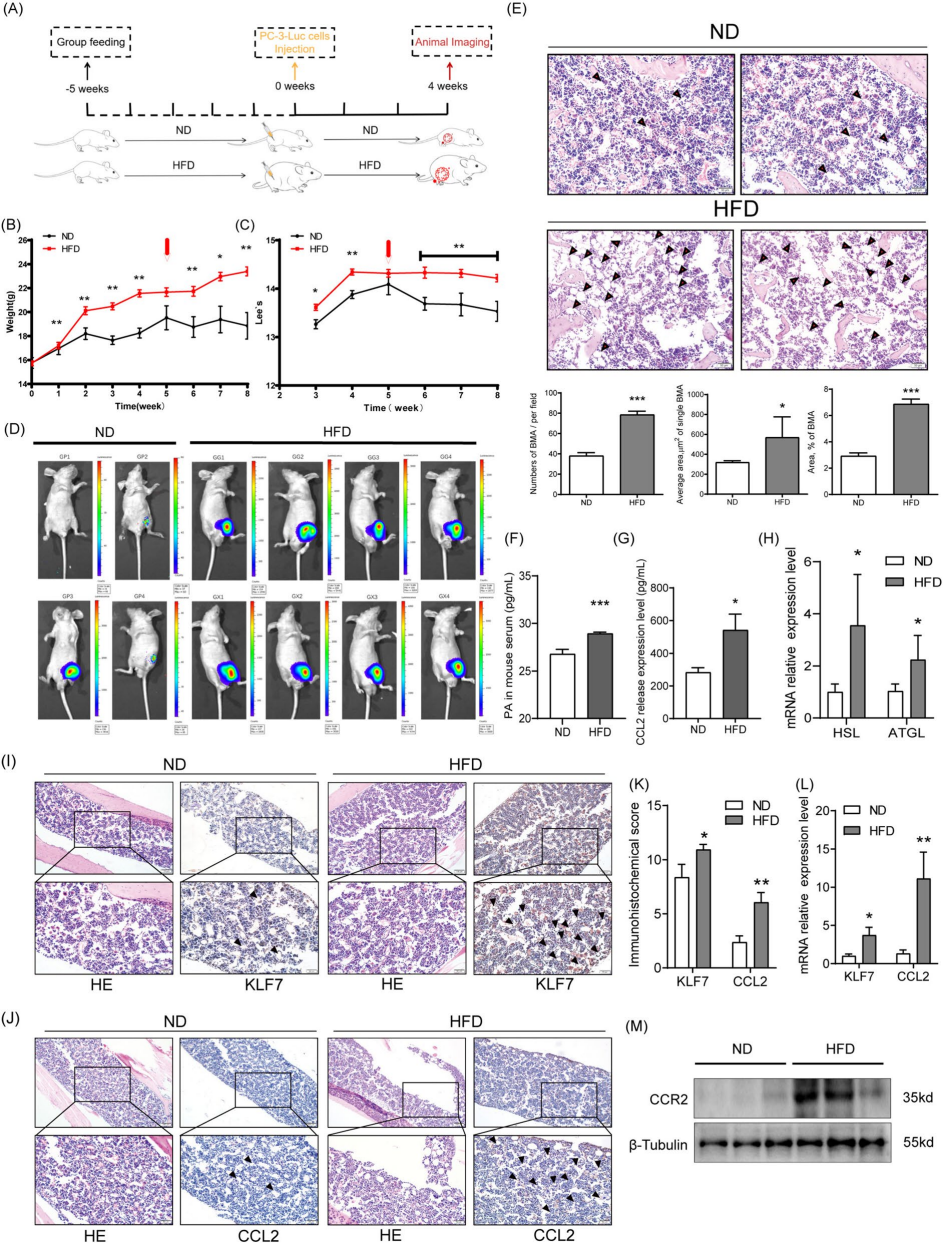
Tumor-forming ability of PCa cells in bone and the expression levels of KLF7/CCL2 in the bone under the conditions of obesity. BALB/c nude mice were fed a 60% high-fat diet. **A** Schema for the bone marrow PCa-bearing mouse model in obese state. **B** The weight and (**C**) Lee’s index were determined for these mice. In the fourth week, fluorescent PC-3-Luc cells were injected into the femur of the left leg of each mouse. Subsequently, (**D**) the Small Animal Imaging Technology was applied. After the H&E staining of the femur of the left leg of the mice, (**E**, Up) the BMA cells with a size of over 50 µm were observed under a microscope at a magnification of 200 × and using the CellSens standard software. (**E**, Down) The number of BMAs, area of a single BMA, and proportion of the BMA area in the bone marrow cavity were determined. PA (**F**) and CCL2 (**G**) levels in mouse serum were determined using ELISA. **H** The mRNA expression levels of HSL/ATGL in bone marrow were determined using qRT-PCR. The protein expression levels of KLF7 (**I**) and CCL2 (**J**) in BMA were determined using immunohistochemistry. **K** The immunohistochemistry score of KLF7 and CCL2. **L** The mRNA expression levels of HSL/ATGL in bone marrow were determined using qRT-PCR. **M** The protein expression level of CCR2 in mouse`s tumor tissue were determined using Western Blot. In the non-parametric rank-sum test, the differences with *P < 0.05, **P < 0.01, and ***P < 0.001 were statistically significant

Initially, to investigate whether HFD feeding promotes tumor growth, the BALB/c nude mouse were fed with a 60% high-fat diet (HFD) (Fig. 1A). After five weeks of HFD feeding, the HFD mouse exhibited 20% higher weight compared to the ND mouse (Fig. 1B). Moreover, Lee’s index and the serum levels of lipids, such as FFA, TG, HDL, and GLU, were evidently higher in the HFD mouse (Fig. 1C, Supplementary Table 2), which suggested that the nude mouse model of obesity had been established successfully. The weight changes of each mice are shown in Supplementary Table 3. Next, fluorescent PC-3-Luc cells were injected into the left femur of each mice. After continuing HFD for three weeks, the small animal imaging technology revealed that the tumorigenesis rate of PC-3-Luc cells (the maximum fluorescence value of over 100) in the left femur of the mouse fed with HFD (8/8, 100%) was higher than ND mouse (1/4, 25%), as shown in Fig. 1D. Therefore, it was inferred that PCa cells are more likely to form tumors in the bones of obese mice.

### Obesity facilitated KLF7/CCL2 expression of BMA in the bone marrow cavity

Further observation of the distribution of BMA in obese individuals revealed that the number of BMA, the area of single BMA, and the proportion of BMA area in the bone marrow cavity in HFD mouse were evidently higher than those in the ND mouse (Fig. 1E). Furthermore, the levels of PA and CCL2 in the serum of HFD mouse were significantly higher than ND mouse (Fig. 1F-G). The mRNA expression levels of Homone sensitive triglyceride lipase (HSL) and Adipose triglyceride lipase (ATGL) in the bone marrow of HFD mouse were also significantly higher (Fig. 1H), indicating that the lipolysis level in BMA further increased in obese status, which may lead to the positive regulation of FFAs content.

To determine whether HFD feeding leads to increased expression of KLF7/CCL2 in the bone marrow cavity, IHC analyses and qRT-PCR experiments were performed in the present study to evaluate KLF7/CCL2 expression levels. The staining scores for KLF7 and CCL2 were evidently higher in the BMA of HFD mouse, which implied that HFD feeding led to higher protein expression levels for both KLF7 and CCL2 (Fig. 1I-K) in the BMA of mice. Understandably, the qRT-PCR results revealed a significant promotion of mRNA levels for both KLF7 and CCL2 (Fig. 1L). Collectively, these results suggested that obesity facilitates KLF7/CCL2 expression of BMA in the bone marrow cavity. In previous studies, we constructed an situ-PCa bearing mouse model in an obese state [28]. It is worth noting that after retesting the CCR2 in these tumor tissues, we found that the expression levels of CCR2 in PCa tumor tissues also increased under obesity, which may be another positive response of tumor cells to search for CCL2 in distal bone marrow (Fig. 1M).

### PA upregulated CCL2 of BMA to facilitate the proliferation, invasion, and migration ability of PCa cells

**Fig. 2:**
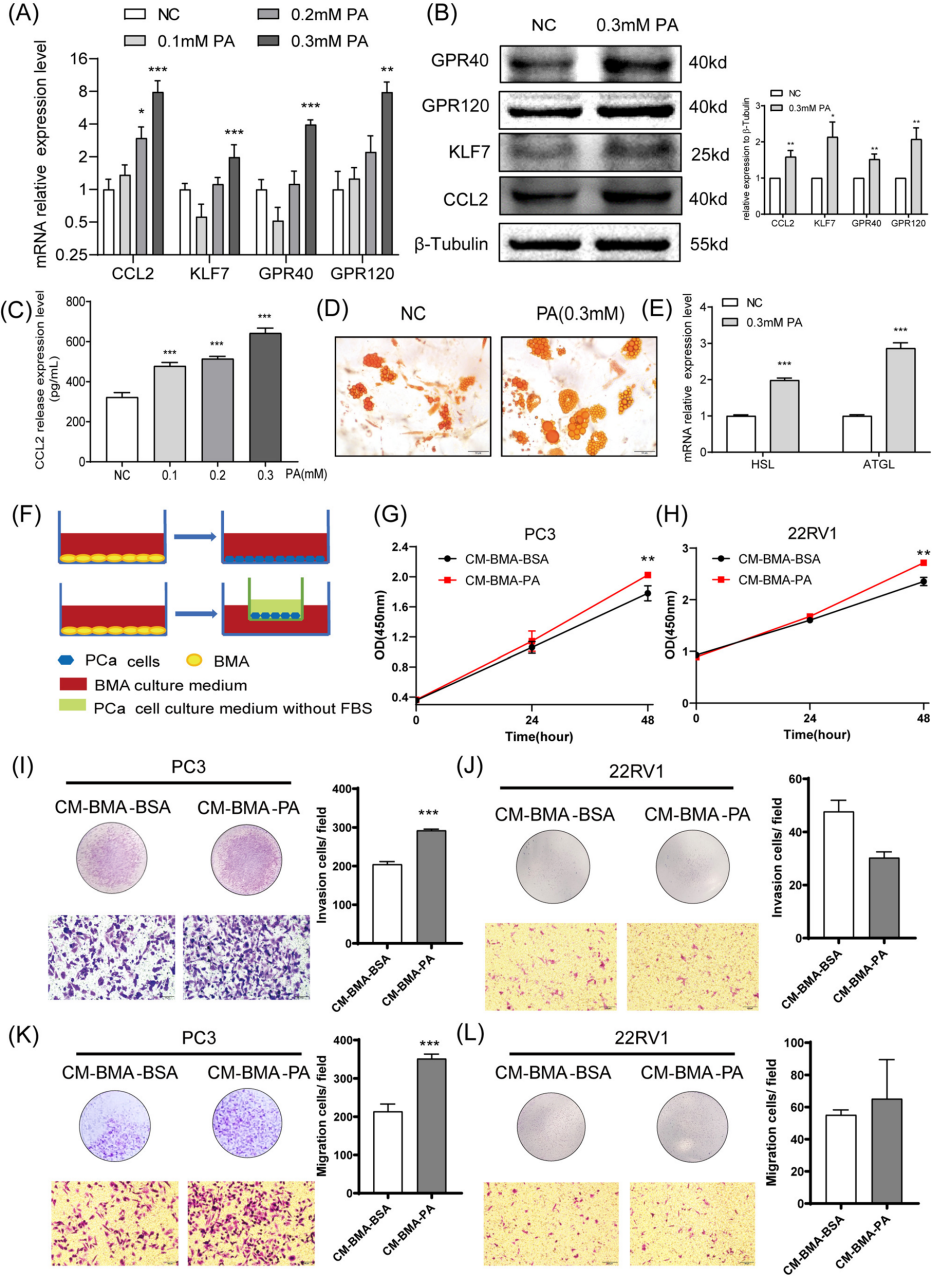
PA upregulated CCL2 in BMA and promoted the proliferation, migration, and invasion abilities of PCa cells. PA was used for stimulating the adipocytic differentiation of MSCs for 48 h. **A** The mRNA expression levels of CCL2/KLF7/GPR40/GPR120 in BMA were determined using qRT-PCR. **B** The protein expression levels of CCL2/KLF7/GPR40/GPR120 in BMA were determined using Western Blot. **C** The CCL2 secretion levels were determined using ELISA. **D** The oil red O staining was performed. **E** The mRNA expression levels of HSL/ATGL in BMA were determined using qRT-PCR. **F** The conditioned medium obtained from the BMA incubated with PA (CM-BMA-PA) was adopted for culturing the PCa cell lines. The (**G**, **H**) proliferation, (**I**, **J**) invasion, and (**K**, **L**) migration abilities of PC-3 cells and 22RV1 cells were determined. In the t-test, the differences with *P < 0.05, **P < 0.01, and ***P < 0.001 were statistically significant

In order to assess whether PA promoted the levels of CCL2 in the BMA model, the expression and secretion levels of CCL2 in BMA were evaluated after incubation with PA for 48 h. It was revealed that PA facilitated the expression and secretion levels of CCL2 in a dose-dependent manner, the same as KLF7/GPR40/GPR120 (Fig. 2A-C). In addition, 0.3 mM PA significantly enhanced the lipolysis level of BMA. (Fig. 2D-E). Next, the PCa cell lines were cultured in a conditioned medium from the BMA cultured with PA (CM-BMA-PA) (Fig. 2F). It was observed that compared to the CM-BMA-BSA group, the PC-3 cells (entirely capable of migrating) co-cultured with the CM-BMA-PA group exhibited augmented proliferation (Fig. 2G), invasion (Fig. 2I), as well as migration (Fig. 2K) ability (*P* < 0.05). Moreover, the 22RV1 cells (with a limited migrating ability) co-cultured with CM-BMA-PA exhibited significantly increased (*P* < 0.05) proliferation ability (Fig. 2H), while the invasion (Fig. 2J) and migration (Fig. 2L) abilities of these cells exhibited no significant difference.

**Fig. 3:**
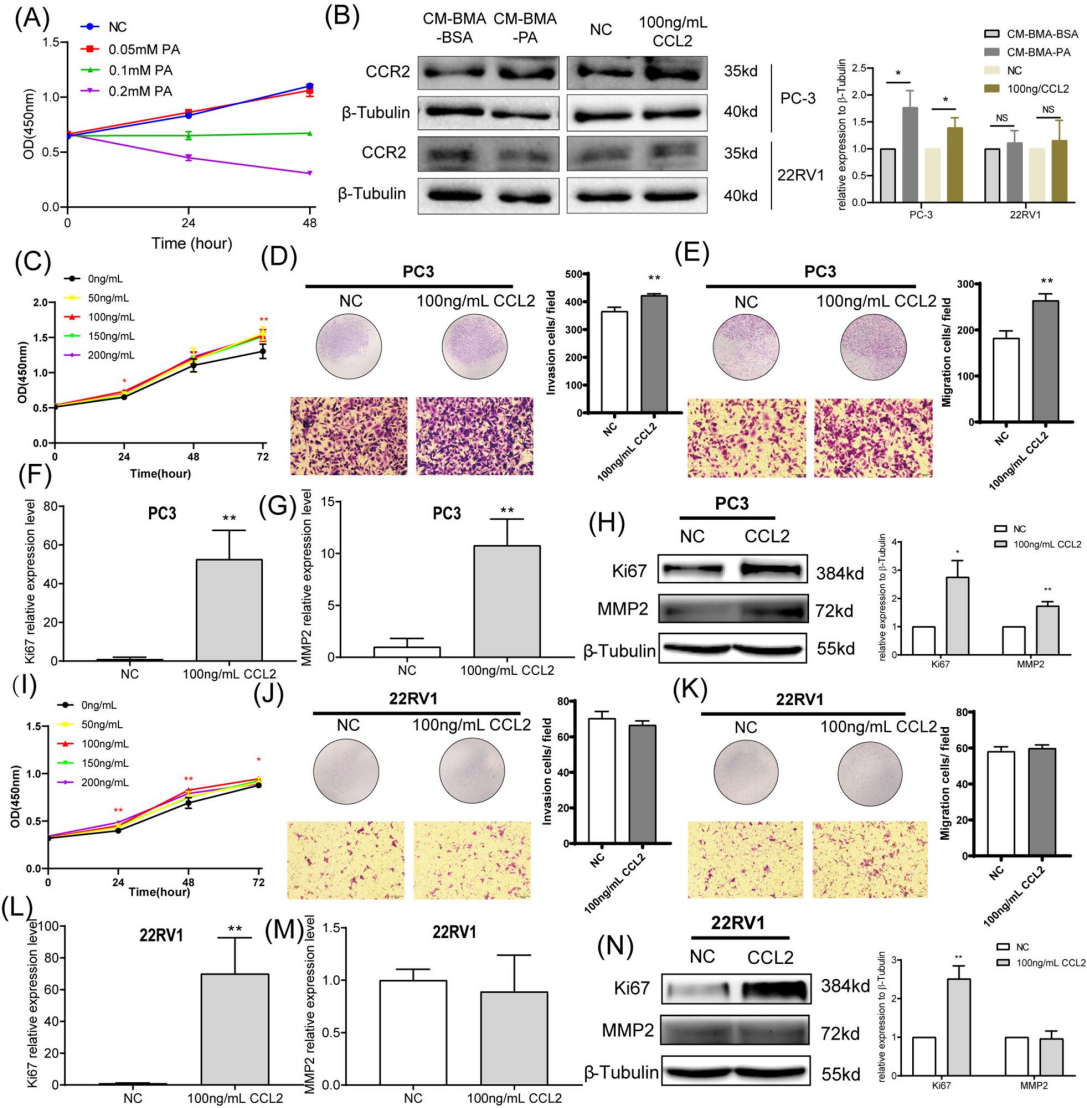
CCL2 stimulated the proliferation, invasion, and migration abilities of PCa cells. **A** After direct stimulation of PC-3 cells with different concentrations of PA, the proliferation level of cells was detected using CCK8. **B** Stimulate PC-3 and 22RV1 cells with CM-BMA-PA or CCL2 recombinant protein, and then detect the expression level of CCR2 in PC-3 and 22RV1 cells. After CCL2 recombinant protein (100 ng/mL) was used for treating PC-3 cells. The proliferation (**C**), invasion (**D**), and migration (**E**) abilities of PC-3 cells were determined. Moreover, qRT-PCR was performed to evaluate the mRNA expression levels of the proliferation-related factor Ki67 (**F**) and the metastasis-related factor MMP2 (**G**). The results of western blotting revealed the protein expression levels for Ki67 and MMP2 (**H**). After CCL2 recombinant protein (100 ng/mL) was used for treating 22RV1 cells. The proliferation (**I**), invasion (**J**), and migration (**K**) abilities of 22RV1 cells were determined. Moreover, qRT-PCR was performed to evaluate the mRNA expression levels of the proliferation-related factor Ki67 (**L**) and the metastasis-related factor MMP2 (**M**). The results of western blotting revealed the protein expression levels for Ki67 and MMP2 (**N**). In the t-test, the differences with *P < 0.05, **P < 0.01, and ***P < 0.001 were statistically significant

To exclude the effect of PA on PCa cells, we used different concentrations of PA to directly stimulate PC-3 cells. Interestingly, pure PA stimulation significantly inhibited the proliferation level of PCa, presenting completely opposite results to CM-BMA-PA (Fig. 3A). This suggests that high concentrations of PA mainly promote bone metastasis of PCa by altering the tumor microenvironment. Furthermore, we treated PC-3 cells and 22RV1 cells with CM-BMA-PA and 100 ng/mL CCL2, respectively. The results showed that both CM-BMA-PA and 100 ng/mL CCL2 significantly promoted the expression level of CCR2 in PC-3 cells, while there was no significant response in 22RV1 cells (Fig. 3B). Perhaps, that's one reason there are different abilities of invasion and migration between two types of PCa cells.

In order to directly explore the role of CCL2 in the biological behavior of tumor cells, 100 ng/mL of the CCL2 recombinant protein was used to evaluate the proliferation, invasion, and migration abilities of PC-3 and 22RV1 cells. The results revealed that the CCL2 recombinant protein significantly promoted (*P* < 0.05) the proliferation, invasion, as well as migration of PC-3 cells (Fig. 3C-E). Moreover, the CCL2 recombinant protein significantly upregulated (*P* < 0.05) the expressions of the proliferation-related factor Ki67 and the metastasis-related factor MMP2 (Fig. 3F–H). The 22RV1 cells exhibited significantly enhanced (*P* < 0.05) proliferation ability and increased Ki67 expression when treated with the CCL2 recombinant protein (Fig. 3I, L, N), while their invasion ability, migration ability, and MMP2 expression levels (Fig. 3J-K, M-N) exhibited no significant differences.

**Fig. 4:**
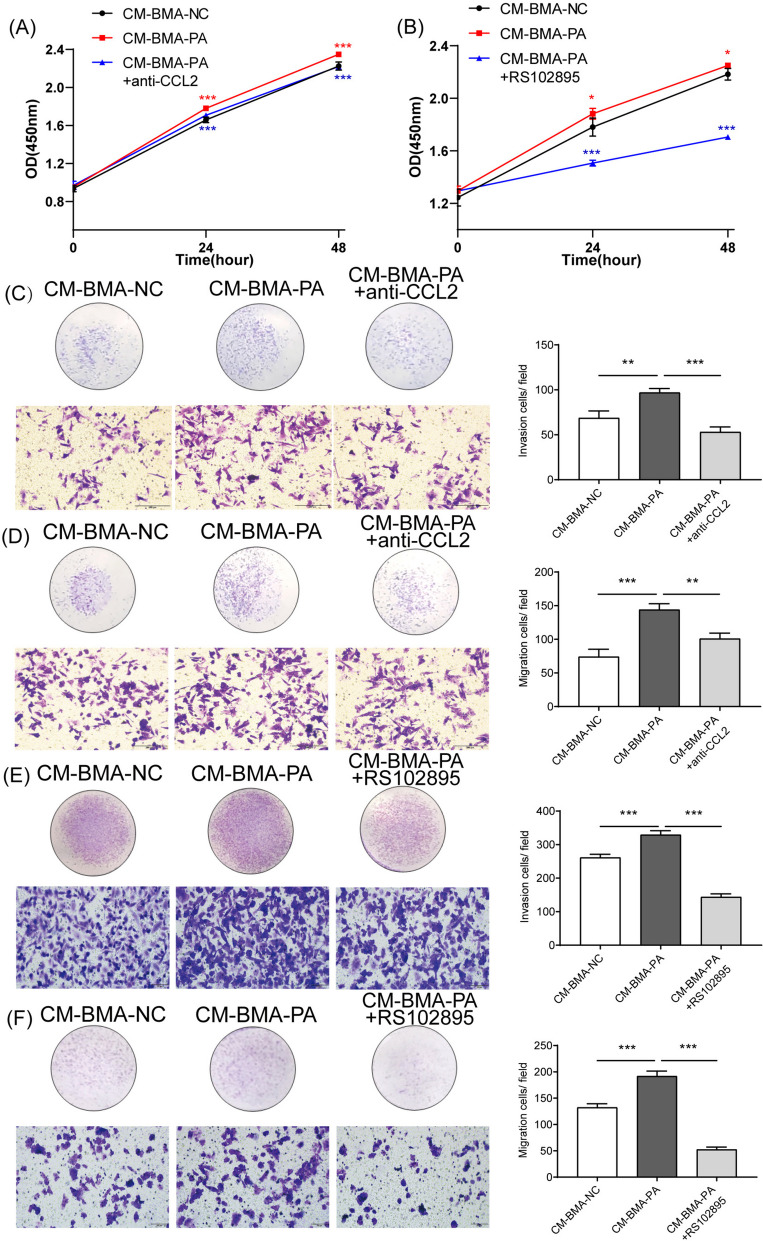
PA upregulated CCL2 in BMA to promote the proliferation, migration, and invasion abilities of PC-3 cells. The CCL2-neutralizing antibody were employed to block the efficiency of CCL2 under PA stimulation. The proliferation (**A**), invasion (**C**), and migration (**D**) abilities of PC-3 cells were determined. The CCR2 antagonist RS102895 were employed to block the efficiency of CCL2 under PA stimulation. The proliferation (**B**), invasion (**E**), and migration (**F**) abilities of PC-3 cells were determined. In the t-test, the differences with *P < 0.05, **P < 0.01, and ***P < 0.001 were statistically significant

Next, the CCL2-neutralizing antibody was used to block the efficiency of CCL2 under PA stimulation. It was observed that the use of the CCL2-neutralizing antibody significantly reversed the CM-BMA-PA-induced deteriorated biological behavior and elevated the expressions of tumor-associated genes in PC-3 cells (Fig. 4A, C-D). Another CCR2 antagonist, RS102895, was used, and it was observed to notably reverse (*P* < 0.05) the proliferation, invasion, and migration abilities of the PC-3 cells co-cultured with CM-BMA-PA (Fig. 4B, E-F). Collectively, these findings indicated that high concentrations of PA facilitated the proliferation, invasion, and migration of PCa cells through the regulation of the CCL2–CCR2 axis in BMA cells.

### PA-induced KLF7/CCL2 pathway in BMA stimulated the proliferation, invasion, and migration abilities of PCa cells

**Fig. 5:**
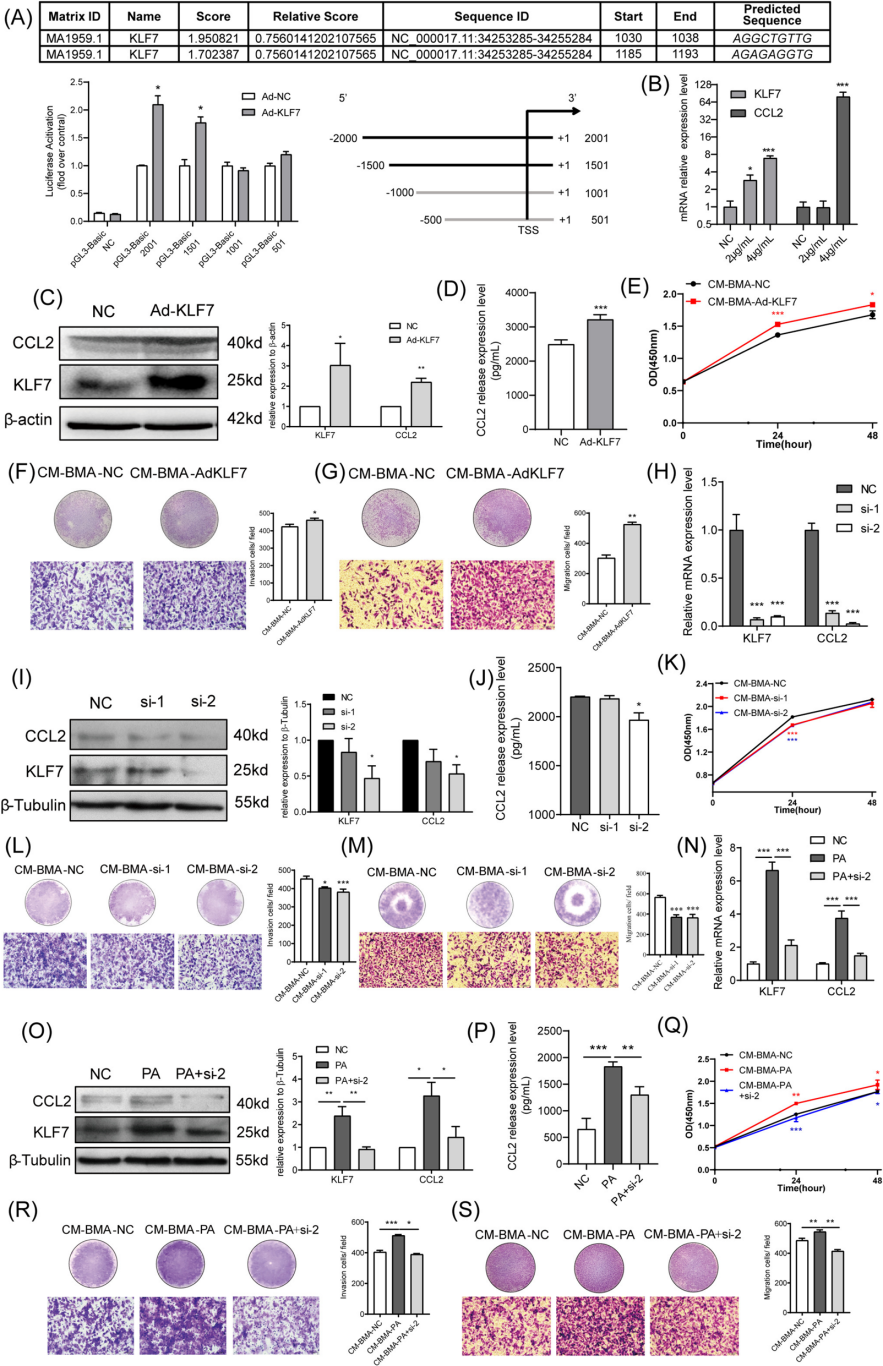
PA-induced KLF7/CCL2 pathway in BMA promoted the malignancy of PC-3 cells. **A** Co-transfection of the human CCL2 promoter region luciferase plasmid and the KLF7-overexpression plasmid into 293 T cells and the detection of the luciferase activity. The KLF7 overexpression plasmid was transfected into BMA cells. The mRNA and protein expression levels of KLF7/ CCL2 (**B**, **C**)and the secretion levels of CCL2 (**D**) were determined. Next, the conditioned medium obtained from the BMA with KLF7 overexpression (CM-BMA-AdKLF7) was used for stimulating the PC-3 cells. The proliferation (**E**), invasion (**F**), and migration (**G**) abilities of PC-3 cells were determined. Afterward, the small interfering RNA for KLF7 (si-KLF7) was transfected into BMA cells. The mRNA and protein expression levels of KLF7/CCL2 (**H**, **I**) and the secretion levels of CCL2 (**J**) were determined. Subsequently, the conditioned medium obtained from the BMA with si-KLF7 (CM-BMA-siKLF7) was used for stimulating PC-3 cells. The proliferation (**K**), invasion (**L**), and migration (**M**) abilities of PC-3 cells were determined. Finally, si-KLF7 was transfected into BMA cells under PA stimulation. The mRNA and protein expression levels of KLF7 and CCL2 were determined (**N**, **O**). The secretion levels of CCL2 were determined (**P**). Afterward, the conditioned medium obtained from the BMA with PA + si-KLF7 (CM-BMA-PA + siKLF7) was used for stimulating PC-3 cells. The proliferation (**Q**), invasion (**R**), and migration (**S**) abilities of PC-3 cells were determined. In the t-test and one-way ANOVA, the differences with *P < 0.05, **P < 0.01, and ***P < 0.001 were statistically significant

To determine whether KLF7 binds to the CCL2 promoter, the 2000-bp region surrounding the CCL2 transcription start site was analyzed, and three CCL2 promoter region truncates were constructed. The possibility of the presence of a KLF7 binding site was detected in the 1501 bp to 1001 bp region of the CCL2 promoter (Fig. 5A). Next, a KLF7-overexpression plasmid and interference fragments were employed to further investigate whether KLF7 could upregulate the CCL2 levels in BMA, thereby leading to the changes in the biological behavior of PCa cells. As illustrated in Fig. 5B-D, KLF7 overexpression significantly increased the CCL2 expression and secretion levels in BMA. Indeed, the proliferation, invasion, and migration of PC-3 cells were remarkably enhanced when co-cultured with CM-BMA-AdKLF7 (Fig. 5E-G). Congruently, the small interfering RNA for KLF7 (si-KLF7) could significantly inhibit the expression and secretion of CCL2 (Fig. 5H-J). In addition, the proliferation, invasion, and migration of PC-3 cells were substantially suppressed when co-cultured with CM-BMA-siKLF7 (Fig. 5K-M). These results suggested that KLF7-induced elevated expression and secretion of CCL2 in BMA promotes the proliferation, invasion, and migration abilities of PCa cells.

The results revealed that PA could significantly upregulate KLF7/CCL2 expression in BMA (Fig. 2A-B). Therefore, to further assess whether KLF7 was involved in the PA-induced production of CCL2, PA-stimulated BMA cells were transfected with si-KLF7. It was observed that CCL2 expression and secretion were significantly inhibited in these cells (Fig. 5N-P). Meanwhile, in contrast to the CM-BMA-PA group, the CM-BMA-PA + siKLF7 group exhibited suppressed proliferation, invasion, and migration abilities of PC-3 cells (Fig. 5Q-S). Collectively, these findings suggested that the PA-induced KLF7/CCL2 pathway in BMA facilitates the proliferation, invasion, and migration abilities of PCa cells.

### PA-activated GPRs/KLF7/CCL2 pathway in BMA facilitated the proliferation, invasion, and migration abilities of PCa cells

**Fig. 6:**
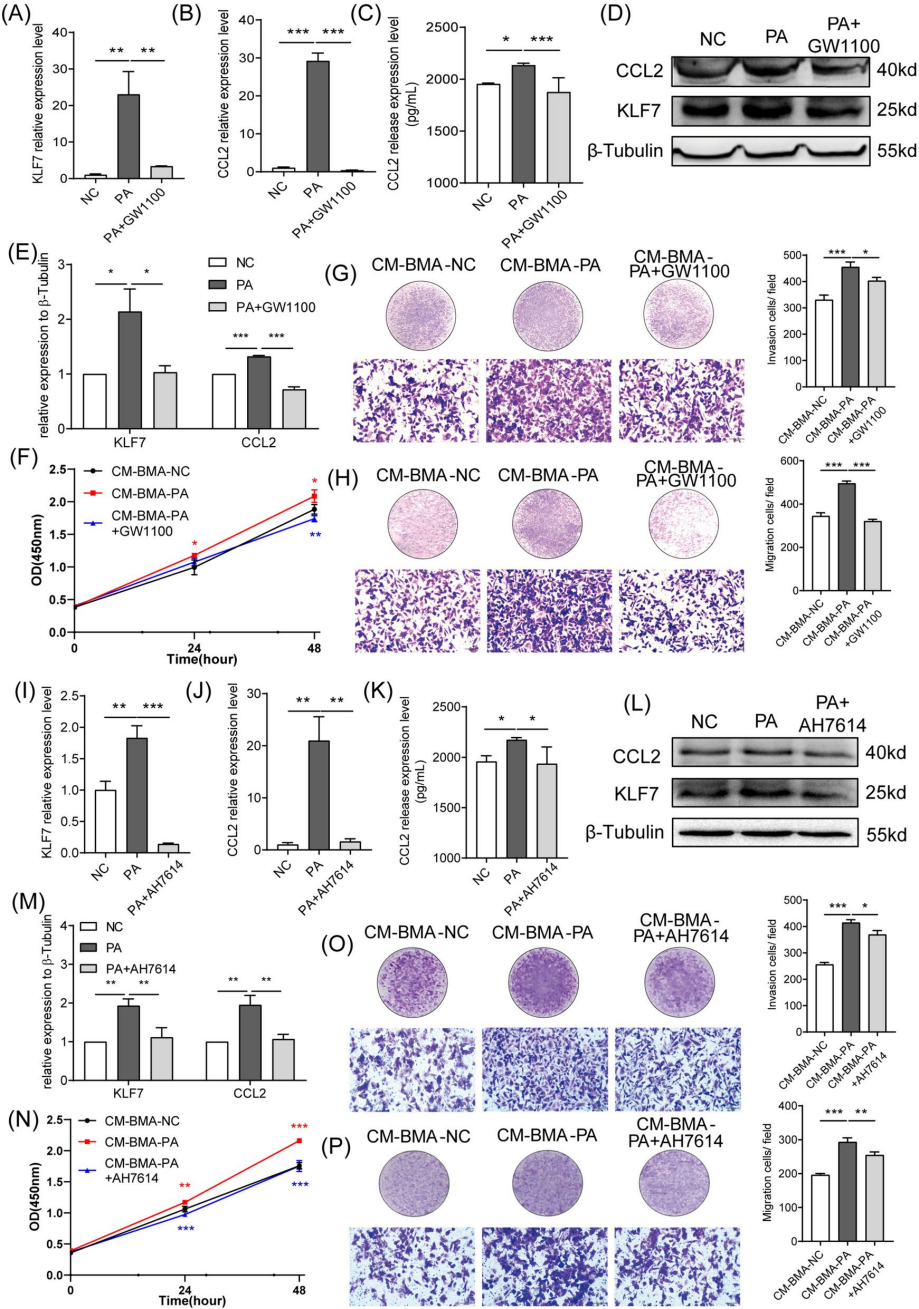
PA-induced KLF7/CCL2 pathway in BMA promoted the malignancy of PC-3 cells via GPR40/120. The GPR40 antagonist GW1100 was added to BMA cells under PA stimulation. The mRNA expression levels of KLF7/ CCL2 (**A**, **B**) and the secretion levels of CCL2 were determined (**C**).The protein expression levels of KLF7/ CCL2 were also determined (**D**, **E**). Next, the conditioned medium obtained from the BMA with PA + GW1100 (CM-BMA-PA + GW1100) was used for stimulating PC-3 cells. The proliferation (**F**), invasion (**G**), and migration (**H**) abilities of PC-3 cells were determined. In addition, The GPR120 antagonist AH7614 was added to BMA cells under PA stimulation. The mRNA expression levels of KLF7/ CCL2 (**I**, **J**) and the secretion levels of CCL2 were determined (**K**).The protein expression levels of KLF7/ CCL2 were also determined (**L**, **M**). Next, the conditioned medium obtained from the BMA with PA + AH7614 (CM-BMA-PA + AH7614) was used for stimulating PC-3 cells. The proliferation (**N**), invasion (**O**), and migration (**P**) abilities of PC-3 cells were determined. In the t-test, the differences with *P < 0.05, **P < 0.01, and ***P < 0.001 were statistically significant

GPR40 and GPR120 are two major ligands of long-chain FFAs, which play vital roles in the PA-induced KLF7/CCL2 pathway. The present study revealed that high concentrations of PA evidently increased the expressions of GPR40 and GPR120 in BMA (Fig. 2A, B). Moreover, it was noted that both GW1100 and AH7614, the antagonists of GPR40 and GPR120, respectively, could dramatically inhibit the expressions of KLF7/CCL2 and the secretion of CCL2 under PA stimulation (Fig. 6A-E, I-M). In addition, the proliferation, invasion, and migration abilities of PC-3 cells were inhibited in both CM-BMA-PA + GW1100 and CM-BMA-PA + AH7614 groups (Fig. 6F-H, N-P). Collectively, these results demonstrated that the PA-induced GPRs/KLF7/CCL2 pathway in BMA promoted the proliferation, invasion, and migration abilities of PCa cells.

### Turkish galls inhibited the PA-induced increase in the KLF7/CCL2 expression in BMA to block the proliferation, invasion, and migration abilities of PCa cells

Finally, a preliminary experiment was performed to explore the effect of Turkish galls, a traditional Chinese medicine, on the expression of KLF7/CCL2 in BMA. It was revealed that Turkish galls could significantly suppress KLF7/CCL2 expression and CCL2 secretion under PA stimulation (Supplementary Fig. 1A-F). Moreover, the proliferation, invasion, and migration abilities of PC-3 cells were inhibited in the CM-BMA-PA + Galls group (Supplementary Fig. 1G-I). Collectively, these results demonstrated that Turkish galls inhibited the PA-induced increase in the KLF7/CCL2 expression in BMA, leading to the blocking of the proliferation, invasion, and migration abilities of PCa cells.

## Discussion

Accumulating evidence suggests a strong association between obesity and bone metastasis in PCa. Therefore, several studies have indicated that the obesity-mediated changes in the bone marrow microenvironment could have a vital role in promoting bone metastasis in PCa [14, 29]. Consistent with this, the present study revealed that HFD mice exhibited a significantly higher number of BMA cells, area of single BMA, and proportion of the BMA area in the bone marrow cavity. Moreover, the HFD mice exhibited promoted tumor growth in the bone marrow cavity.

Previous studies and laboratory data have demonstrated that obesity-induced high levels of FFAs, such as arachidonic acid and caprylic acid, altered the bone marrow microenvironment, thereby creating a “fertile soil” for PCa bone metastasis to occur [27, 30]. It is reported that PA is the most abundant among all FFAs in the bone marrow supernatant fluid [31]. However, the role of PA in the crosstalk between bone marrow microenvironment and PCa bone metastasis has not been completely elucidated so far. PA could reportedly stimulate the osteoblasts in the bone marrow cavity to secrete higher levels of CCL2 [31, 32]. The increased levels of CCL2 in the bone marrow cavity created a unique inflammatory and chemotactic microenvironment in bone marrow, which could be an important reason underlying the convenient colonization of bone in PCa [17]. Certain studies have also indicated that high concentrations of PA facilitate the expressions of various cytokines in mouse adipocytes, including CCL2 [33]. In the present study, PA was observed to upregulate CCL2 in BMA to promote the proliferation, invasion, and migration abilities of PC-3 cells. It is worth noting that under the stimulation of CCL2, all CCK8 results showed that the proliferation ability of PC-3 was not significantly enhanced. This may be due to the fact that CCL2 mainly acts through chemotactic attraction, and under its stimulation, tumor cells tend to enhance their invasion and migration ability rather than metastasis ability [34]. On the contrary, an increase in only the proliferation ability was observed in the 22RV1 cells co-cultured with CM-BMA-PA and those treated with the CCL2 recombinant protein. This could be explained by the unaltered expression levels of the metastasis correlation factor MMP2.

KLF7 participates in the regulation of various physiological functions, such as cell proliferation, differentiation, and apoptosis [35]. KLF7 was also reported to exhibit a significantly positive correlation with malignancy development in glioma, gastric cancer, and oral squamous cell carcinoma [36–38]. Recent studies have suggested that high concentrations of PA stimulate KLF7 expression, while KLF7 upregulates the mRNA expression levels of CCL2 in 3T3-L1 adipocytes [19]. The present study, which was conducted on a BMA model, demonstrated that PA promoted CCL2 expression and secretion via increasing the KLF7 expression. Moreover, the PA-induced KLF7/CCL2 pathway in BMA was revealed to facilitate the proliferation, invasion, and migration abilities of PCa cells.

GPR40 and GPR120 usually serve as the receptors of PA and are involved in the regulation of downstream signaling pathways [20]. In the present study, the antagonists of both GPR40 and GPR120 dramatically inhibited the expression of KLF7/CCL2 under PA stimulation. Furthermore, the proliferation, invasion, and migration abilities of PC-3 cells were inhibited in both GPR40 and GPR120 antagonist groups. These results suggested that the PA-induced GPRs/KLF7/CCL2 pathway in BMA facilitated the proliferation, invasion, and migration abilities of PCa cells. Our recent study also found that obesity-induced palmitic acid elevation promotes inflammation and glucose metabolism disorders in mouse adipocytes through the GPRs (GPR40 and GPR120)/NF-κB/KLF7 signaling pathway, in which the active subunit p65 of NF-κB activates KLF7 expression by targeted transcription after nuclear entry [39]. This may be one of the important pathways through which PA promotes KLF7 expression through GPR40/120.

Turkish galls is a traditional Chinese medicine comprising 50%–70% gallotannin and a small amount (2%–4%) of gallic acid and ellagic acid, is an insect gall produced by the larva of Cynips gallae-tinctoriae Oliv [40]. In recent years, the role of Turkish Galls in wound healing, anti-inflammatory, antiviral, antibacterial and anti-ulcer has been gradually explored, and it has been proved to have a good potential anti-inflammatory value [41, 42]. Interestingly, Gallotannin, the main ingredient of Turkish Galls, has been reported by many articles for its good anti-cancer effect. Mun JG et al. found that Gallotannin could inhibit the migration and invasion ability of colorectal cancer cells by inhibiting the expression and activity of MMP-2 and MMP-9 [43]. Jiraporn Kantapan et al. found that Maprang Seed Extract, enriched with Gallotannin, can significantly enhanced the radiosensitivity of breast cancer cells [44]. Notably, Eunkyung Park et al. found that Gallotannin could significantly induce apoptosis of three prostate cancer cells (DU145, PC-3, and M2182) [45]. In the present study, the effects of Turkish galls on the expression of KLF7/CCL2 in BMA were explored. The results demonstrated that Turkish galls inhibited the PA-induced increase in the KLF7/CCL2 expression in BMA to block the proliferation, migration, and invasion abilities of PCa cells. Combined with the study of Eunkyung Park et al., Turkish Galls may have therapeutic effects on both PCa cells and their surrounding microenvironment, which is expected to become a new and low-cost treatment drug for PCa. However, detailed research is required to elucidate the pharmacokinetics and safety of Turkish galls.

## Conclusion

In conclusion, the present study demonstrated that the PA-activated GPRs/KLF7/CCL2 pathway in BMA facilitates the proliferation, invasion, and migration abilities of PCa cells. The findings of the present study would provide a theoretical basis and novel therapeutic targets for the prevention and treatment of obesity-induced bone metastases in PCa.

### Supplementary Information

**Additional file 1: Supplementary Table 1.** The primer sequences. **Supplementary Table 2. **Blood lipids and glucose levels in mice. **Supplementary Table 3. **The weight of each mice changed along the weeks.

**Additional file 2: Supplementary Figure 1.** Turkish galls inhibited the PA-induced increase in the KLF7/CCL2 expression in BMA to block the proliferation, migration, and invasion abilities of PC-3 cells.

**Additional file 3.** ARRIVE checklist.

**Additional file 4.** WB original images

## Data Availability

The main data involved in this study has been provided in the supplementary materials, and other data that supports the findings of this study are available from the corresponding author on reasonable requests.

## Abbreviations

BMA: Bone marrow adipocytes
CCL2: C–C Chemokine Ligand 2
FFAs: Free fatty acids
GPR40/120: G Protein-coupled receptor 40/120
HFD: High fat diet
Ki67: Nuclear-associated antigen
KLF7: Krüppel-Like Factor 7
MMP2: Matrix Metalloproteinase 2
ND: Normal diet
PA: Palmitic acid
PCa: Prostate cancer
