# Supplementary material for: Palmitic acid-activated GPRs/KLF7/CCL2 pathway is involved in the crosstalk between bone marrow adipocytes and prostate cancer
Source: BMC Cancer. 2024 Jan 15;24:75. doi: 10.1186/s12885-024-11826-5 (PMC10789002; doi:10.1186/s12885-024-11826-5)
Supplement: Supplementary file 1 — Additional file 1: Supplementary Table 1. The primer sequences. Supplementary Table 2. Blood lipids and glucose levels in mice. Supplementary Table 3. The weight of each mice changed along the weeks. [file 12885_2024_11826_MOESM1_ESM.docx]

**Supplementary Table 1. The primer sequences**

| Species | Primer | Sequences(5’→3’) | Size |
| --- | --- | --- | --- |
| **Mouse primer** | Mouse-*KLF7*–F | TCCACGACACCGGCTACTT | 202bp |
|  | Mouse-*KLF7*–R | GGGAGCAGCAAGGGGTCTA |  |
|  | Mouse-*CCL2*–F | GAGGCAGAGGCAGGCAACTTC | 265bp |
|  | Mouse-*CCL2*–R | TGGTCCTTAGCAGCATTGTGATGG |  |
|  | Mouse-*HSL*–F | GATTTACGCACGATGACACAGT | 114bp |
|  | Mouse-*HSL*–R | ACCTGCAAAGACATTAGACAGC |  |
|  | Mouse-*ATGL*–F | GGGTGCGCTATGTGGATGG | 91bp |
|  | Mouse-*ATGL*–R | CTCTCGCCTGAGAATGGGG |  |
|  | Mouse-*β-actin*–F | CATTGCTGACAGGATGCAGA | 150bp |
|  | Mouse-*β-actin*–R | CTGATCCACATCTGCTGGAA |  |
| **Human primer** | *Human-GPR40–F* | GCTGCTCTGCGTAGGACCCTA | 125bp |
|  | *Human-GPR40–R* | CCAGCGGATTAAGCACCACACT |  |
|  | *Human-GPR120–F* | ACCTCGGAACACCTCCTGGATG | 110bp |
|  | *Human-GPR120–R* | ATCTGGTGGCTCTCCGAGTAGG |  |
|  | *Human-KLF7-F* | CTCAATGGTGGTGCTTGCTT | 233bp |
|  | *Human-KLF7-R* | TGGAAAACCTGCTCGCTCTA |  |
|  | *Human-CCL2–F* | GGCACCGCAAGGCTCAGAAG | 339bp |
|  | *Human-CCL2–R* | GCATAGCAGGCAGCAGTGGAC |  |
|  | *Human-Ki67–F* | GAAAGAGTGGCAACCTGCCTTC | 151bp |
|  | *Human- Ki67–R* | GCACCAAGTTTTACTACATCTGCC |  |
|  | *Human-MMP2-F* | AGCGAGTGGATGCCGCCTTTAA | 138bp |
|  | *Human-MMP2-R* | CATTCCAGGCATCTGCGATGAG |  |
|  | *Human- HSL-F* | TCAGTGTCTAGGTCAGACTGG | 135bp |
|  | *Human-HSL-R* | AGGCTTCTGTTGGGTATTGGA |  |
|  | *Human- ATGL-F* | GGCTTCCTCGGCGTCTACTA | 230bp |
|  | *Human-ATGL-R* | TTTACCAGGTTGAAGGAGGGG |  |
|  | *Human- GAPDH-F* | GGTGGTCTCCTCTGACTTCAA | 211bp |
|  | *Human-GAPDH-R* | TCTTCCTCTTGTGCTCTTGCT |  |
| **Human small interfering RNA** | KLF7 (si-1)  KLF7-Homo-555 | GCCUUGAAUUGGAACGCUATT |  |
|  |  | UAGCGUUCCAAUUCAAGGCTT |  |
|  | KLF7 (si-2)  KLF7-Homo-2007 | GGUGAGGACUUGGACUGUUTT |  |
|  |  | AACAGUCCAAGUCCUCACCTT |  |

**Supplementary Table 2. Blood lipids and glucose levels in mice**

| **indexes** | **NC(n=4)** | **HFD(n=8)** | ***P*** |
| --- | --- | --- | --- |
| **FFA(mmol/mL)** | 0.56±0.37 | 1.78±0.36 | 0.001 |
| **TG(mmol/mL)** | 0.49±0.17 | 0.78±0.22 | 0.038 |
| **TC(mmol/mL)** | 9.08±1.77 | 10.33±1.96 | 0.310 |
| **HDL(mmol/mL)** | 1.64±0.57 | 2.98±0.56 | 0.003 |
| **LDL(mmol/mL)** | 1.13±0.42 | 1.26±0.29 | 0.538 |
| **GLU(mmol/mL)** | 5.84±4.64 | 12.77±4.56 | 0.033 |

*t* test, *P*<0.05, the difference was statistically significant.

**Supplementary Table 3. The weight of each mice changed along the weeks**

| **Weeks**  **Groups** | **ND(g)** | | | | **HFD(g)** | | | | | | | |
| --- | --- | --- | --- | --- | --- | --- | --- | --- | --- | --- | --- | --- |
|  | **ND1** | **ND2** | **ND3** | **ND4** | **HFD1** | **HFD2** | **HFD3** | **HFD4** | **HFD5** | **HFD6** | **HFD7** | **HFD8** |
| 1 | 14.70 | 15.50 | 16.80 | 16.00 | 16.30 | 15.20 | 16.00 | 15.20 | 15.70 | 14.90 | 16.60 | 15.90 |
| 2 | 15.53 | 16.91 | 17.57 | 17.85 | 17.35 | 16.56 | 18.58 | 16.84 | 16.73 | 16.35 | 18.00 | 17.16 |
| 3 | 17.30 | 17.70 | 19.50 | 18.30 | 19.40 | 20.70 | 19.90 | 20.10 | 19.30 | 20.30 | 22.00 | 19.20 |
| 4 | 16.90 | 17.20 | 18.20 | 18.30 | 19.90 | 21.20 | 20.60 | 20.20 | 19.80 | 20.60 | 22.00 | 19.50 |
| 5 | 17.20 | 18.00 | 18.90 | 18.90 | 20.30 | 22.40 | 21.70 | 21.50 | 21.10 | 21.20 | 23.10 | 21.20 |
| 6 | 18.80 | 17.10 | 20.50 | 21.70 | 20.20 | 22.40 | 21.90 | 21.70 | 20.90 | 21.50 | 23.60 | 21.10 |
| 7 | 18.70 | 16.50 | 19.10 | 20.70 | 20.20 | 22.90 | 21.70 | 21.70 | 21.10 | 21.20 | 23.90 | 21.20 |
| 8 | 19.70 | 16.60 | 19.20 | 22.00 | 21.60 | 24.40 | 23.00 | 22.40 | 22.70 | 22.40 | 24.50 | 22.70 |
