# Supplementary material for: Palmitic acid-activated GPRs/KLF7/CCL2 pathway is involved in the crosstalk between bone marrow adipocytes and prostate cancer
Source: BMC Cancer. 2024 Jan 15;24:75. doi: 10.1186/s12885-024-11826-5 (PMC10789002; doi:10.1186/s12885-024-11826-5)
Supplement: Supplementary file 2 — Additional file 2: Supplementary Figure 1. Turkish galls inhibited the PA-induced increase in the KLF7/CCL2 expression in BMA to block the proliferation, migration, and invasion abilities of PC-3 cells. [file 12885_2024_11826_MOESM2_ESM.docx]

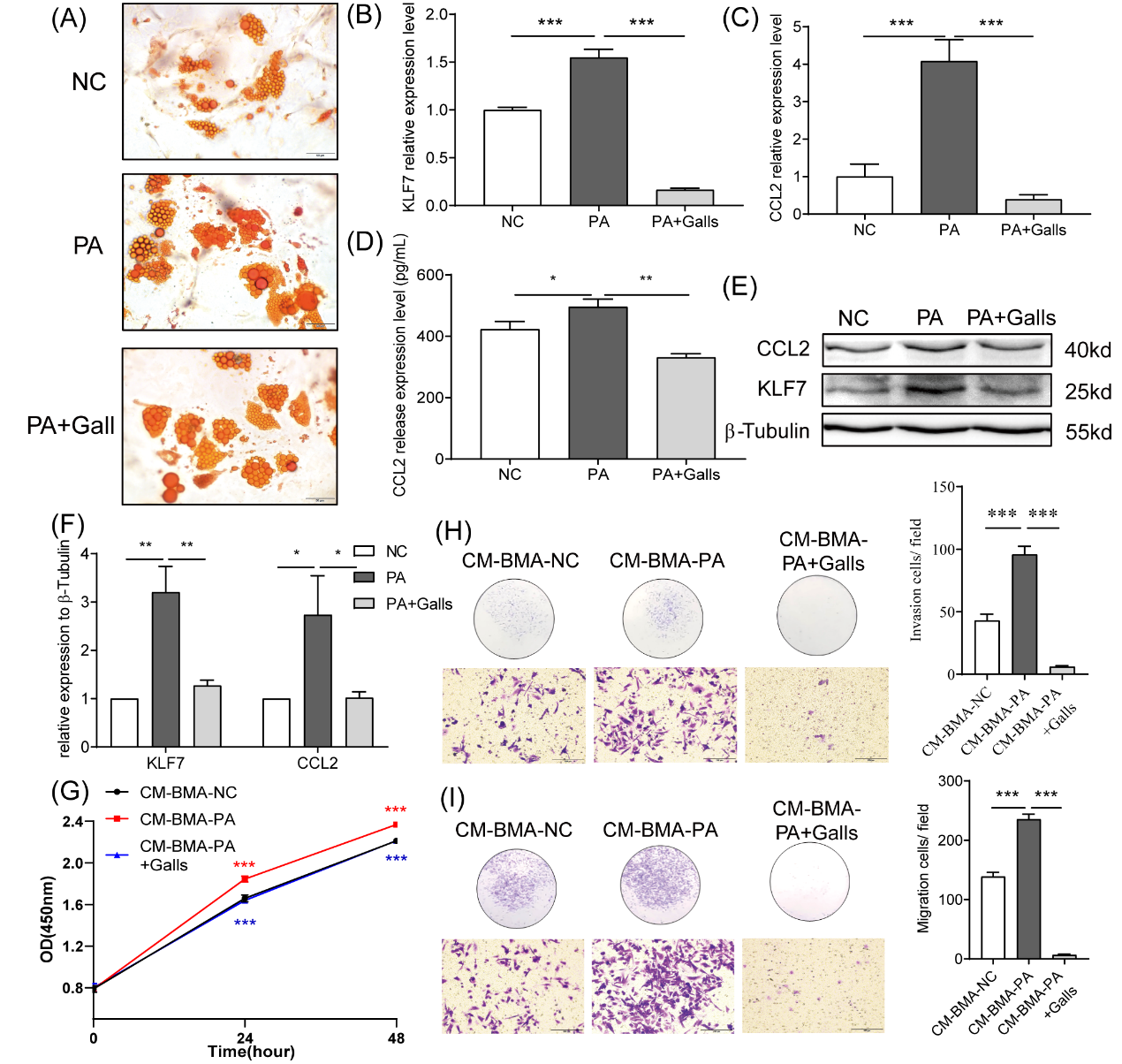


**Supplementary Figure 1. Turkish galls inhibited the PA-induced increase in the KLF7/CCL2 expression in BMA to block the proliferation, migration, and invasion abilities of PC-3 cells.**

Turkish galls were added to BMA cells under PA stimulation. (A) The oil red O staining was performed. The mRNA and protein expression levels of KLF7/CCL2 and the secretion levels of CCL2 were determined (B-F). Next, the conditioned medium obtained from the BMA with PA+ Galls (CM-BMA- PA+Galls) was used for stimulating PC-3 cells. The proliferation (G), invasion (H), and migration (I) abilities of PC-3 cells were determined. In the one-way ANOVA, the differences with **P* < 0.05, ***P* < 0.01, and ****P* < 0.001 were statistically significant.
