# Supplementary material for: Palmitic acid-activated GPRs/KLF7/CCL2 pathway is involved in the crosstalk between bone marrow adipocytes and prostate cancer
Source: BMC Cancer. 2024 Jan 15;24:75. doi: 10.1186/s12885-024-11826-5 (PMC10789002; doi:10.1186/s12885-024-11826-5)
Supplement: Supplementary file 3 — Additional file 3. ARRIVE checklist. [file 12885_2024_11826_MOESM3_ESM.docx]

**ARRIVE checklist**

**1. Study Design (Page 5, Methods- 1. Animals)**

To investigate whether the high expression of KLF7/CCL2 in BMA is related to obesity-induced PCa bone metastasis, male BALB/ C nude mice were fed with high-fat diet (HFD, 60% fat Kcal%) to construct a mice obesity model.

Test group: Fed High-Fat Diet (HFD, 60% fat Kcal%, Medicine, Jiangsu, China).

Control group: Normal Diet (ND, 10% fat Kcal%, Medicine, Jiangsu, China).

After the two groups of mouse showed significant differences in body weight and Lee`s, 5×10^5^ PC-3-Luc cells were injected into the femur of the left leg of each mouse [1].

**2. Sample size (Page 5, Methods- 1. Animals)**

In order to reduce the number of animal sacrifices and ensure the success rate of operation in experimental mice, we adopted a small sample size design.

Test group: n=8

Control group: n=4

**3. Inclusion and exclusion criteria (Page 5, Methods- 1. Animals)**

Mice were included in the study if they survived surgery after bone injection of PC-3 cells into the femur of the left leg. Mice that died or were unable to eat or move normally after surgery were excluded prematurely.

**4. Randomisation (Page 5, Methods- 1. Animals)**

All male mice were randomly assigned to Test group and Control group before group feeding, and then fed in groups of 4 in a cage. Among them, 8 mouse in Test group were randomly assigned to 2 cages and fed with high fat.

To ensure the normal survival of mouse, mouse in both groups ate freely.

**5. Blinding (Page 5, Methods- 1. Animals)**

Four researchers participated in the animal study. The first researcher was responsible for group feeding and numbering of the mice, the second researcher was responsible for in-bone injection surgery (the group of mice was unknown), the third researcher was responsible for vivo imaging and surgical dissection of tissue from the mice (the group of mice was unknown), the fourth researcher was responsible for analyzing the expression levels of various factors in the tissue according to the number (the group of mice was unknown), and sent the data to the first researcher for analysis.

**6. Outcome measures (Page 10, Result 1)**

In this experiment, the body weight and body length of mice were dynamically detected throughout the whole process. Finally, the tumor formation of PCa cells in mouse bone marrow was observed, FFA/TG/TC/HDL/LDL/GLU/PA/CCL2 in serum were detected, and protein and mRNA expression levels of various factors in bone marrow were detected.

**7. Statistical methods (Page 10, Methods- 13. Statistical analysis)**

SPSS (v. 17.0) computer software was used for all statistical analysis. Mean and standard deviation were determined as the main parameters, and the average of data between the experimental and control groups were compared using *Non-parametric rank sum* test. Values of *P* <0.05 as a standard of significant difference.

**8. Experimental animals (Page 5, Methods- 1. Animals)**

Male BALB/ C nude mice was used in this study and all mouse were 4 weeks old (Vital River, Beijing, China).

**9. Experimental procedures (Page 5, Methods- 1. Animals)**

Twelve 4-week-old male mouse were raised in the specific pathogen free animal room. All food and drinking water are strictly sterilized and free to ingest. After a week of adaptive feeding, the mice were fed High-Fat Diet and Normal Diet. After the two groups of mice showed significant differences in body weight and Lee`s, PC-3-Luc cells were injected at a density of 5×10^5^ into the femur of the left leg of each mouse. The femoral wounds were sealed using bone wax. HFD feeding continued for 3 weeks after surgery, they were given inhalation anesthetized using isoflurane, inject D-luc substrate enzyme into the abdominal cavity and observe the PCa tumor formation within 30 minutes using a small animal in vivo imaging instrument, cervical dislocation method made mice die and surgically felt the femur tissues.

**10. Results (Page 10, Result 1)**

The body length of mice were measured with vernier calipers.

The weight of mice were weighed by electronic scale.

In vivo small animal imager was used to observe the tumor formation of PCa cells in mice.

Serum FFA/TG/TC/HDL/LDL/GLUPA/CCL2 in mice were detected by the kit.

Immunohistochemistry was used to detect the protein expression levels of various factors in bone marrow.

qRT-PCR was used to detect the mRNA expression levels of each factor in bone marrow.

References

1. Dai J, Hensel J, Wang N, Kruithof-de Julio M, Shiozawa Y: **Mouse models for studying prostate cancer bone metastasis**. *Bonekey Rep* 2016, **5**:777.
