# Supplementary material for: Palmitic acid-activated GPRs/KLF7/CCL2 pathway is involved in the crosstalk between bone marrow adipocytes and prostate cancer
Source: BMC Cancer. 2024 Jan 15;24:75. doi: 10.1186/s12885-024-11826-5 (PMC10789002; doi:10.1186/s12885-024-11826-5)

Figure 1 M

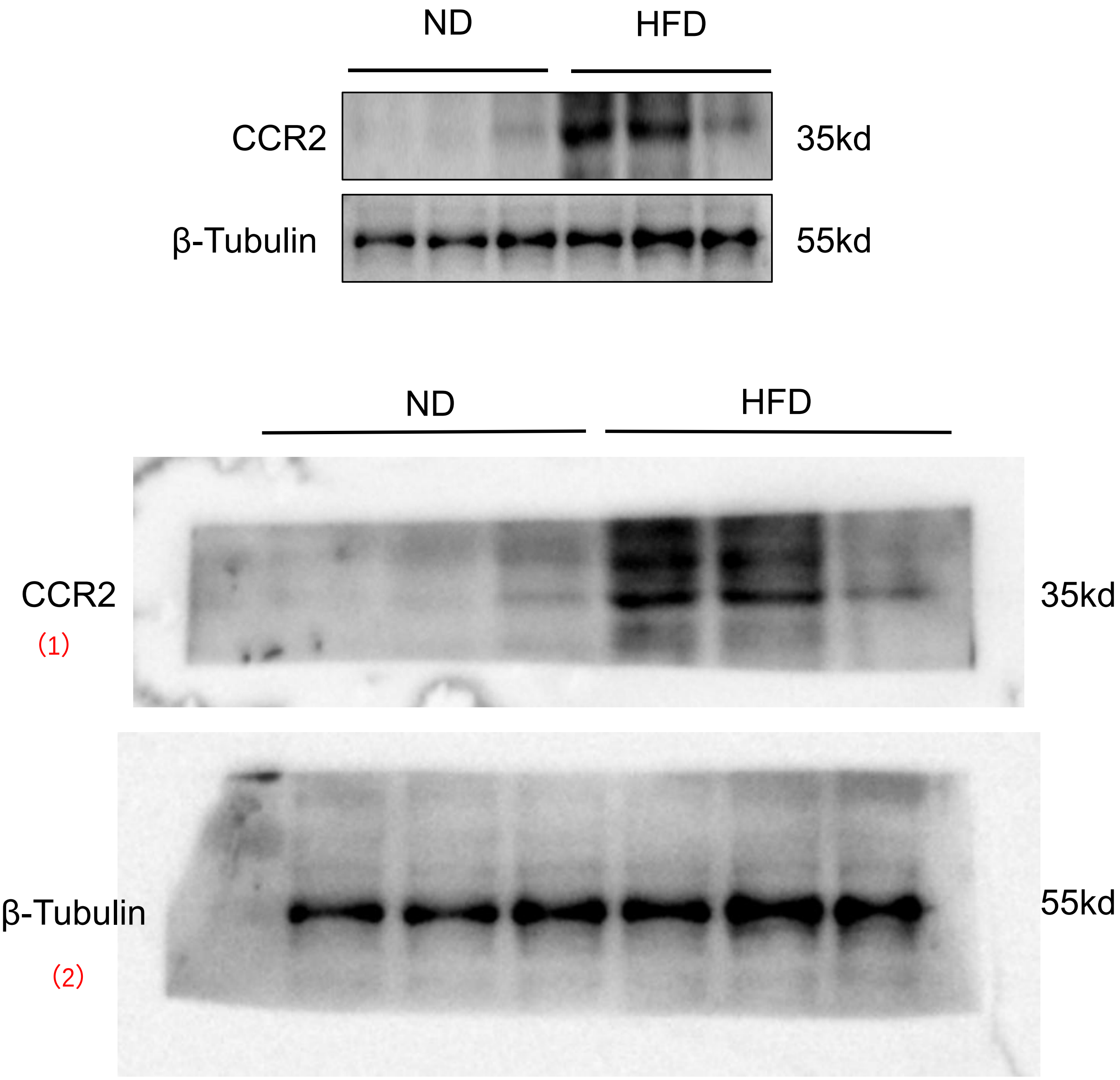
ND
HFD
CCR2
35kd
β-Tubulin
55kd
ND
HFD
CCR2
(1)
35kd
β-Tubulin
(2)
55kd

Original membrane for (1)

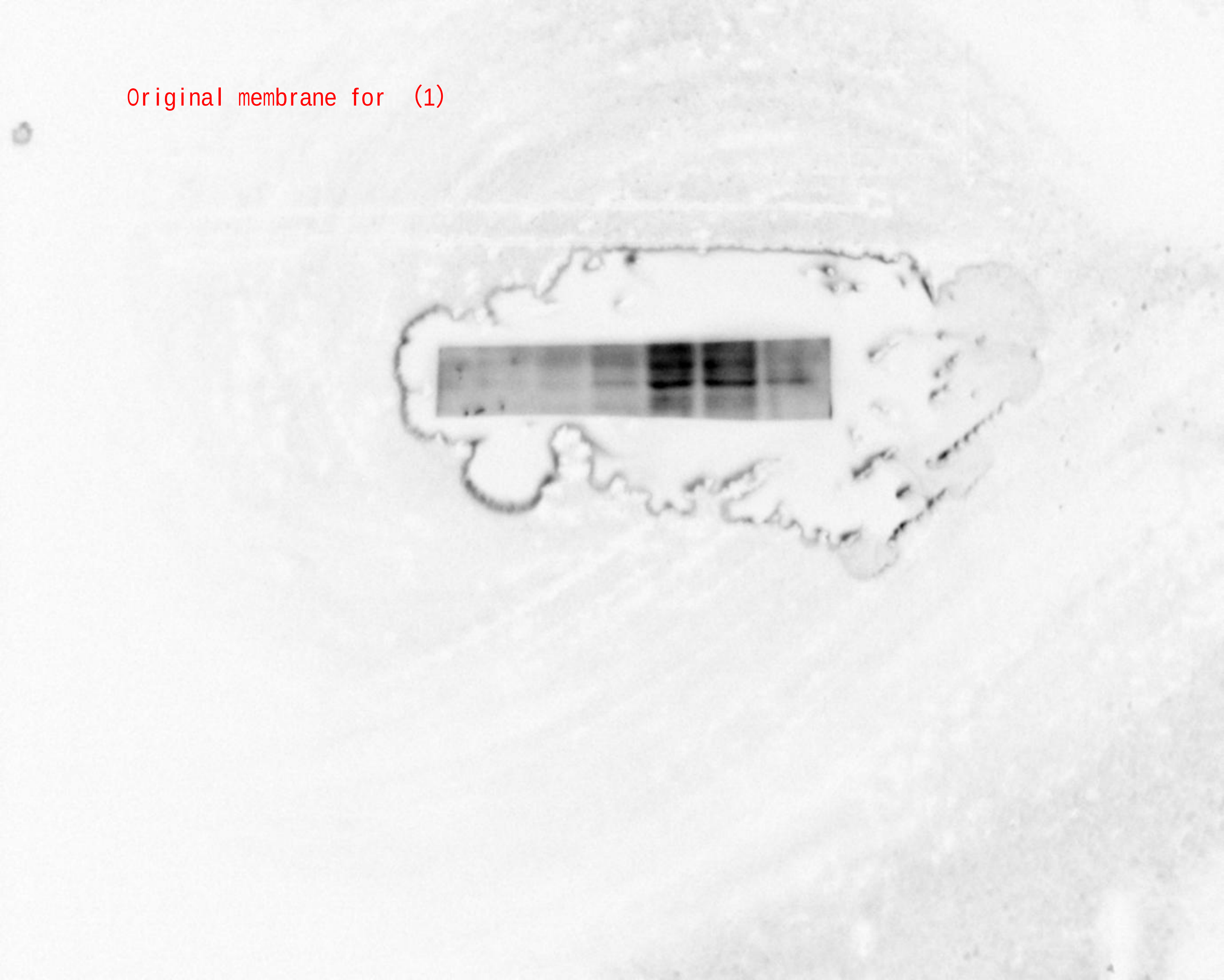

Original membrane for (2)

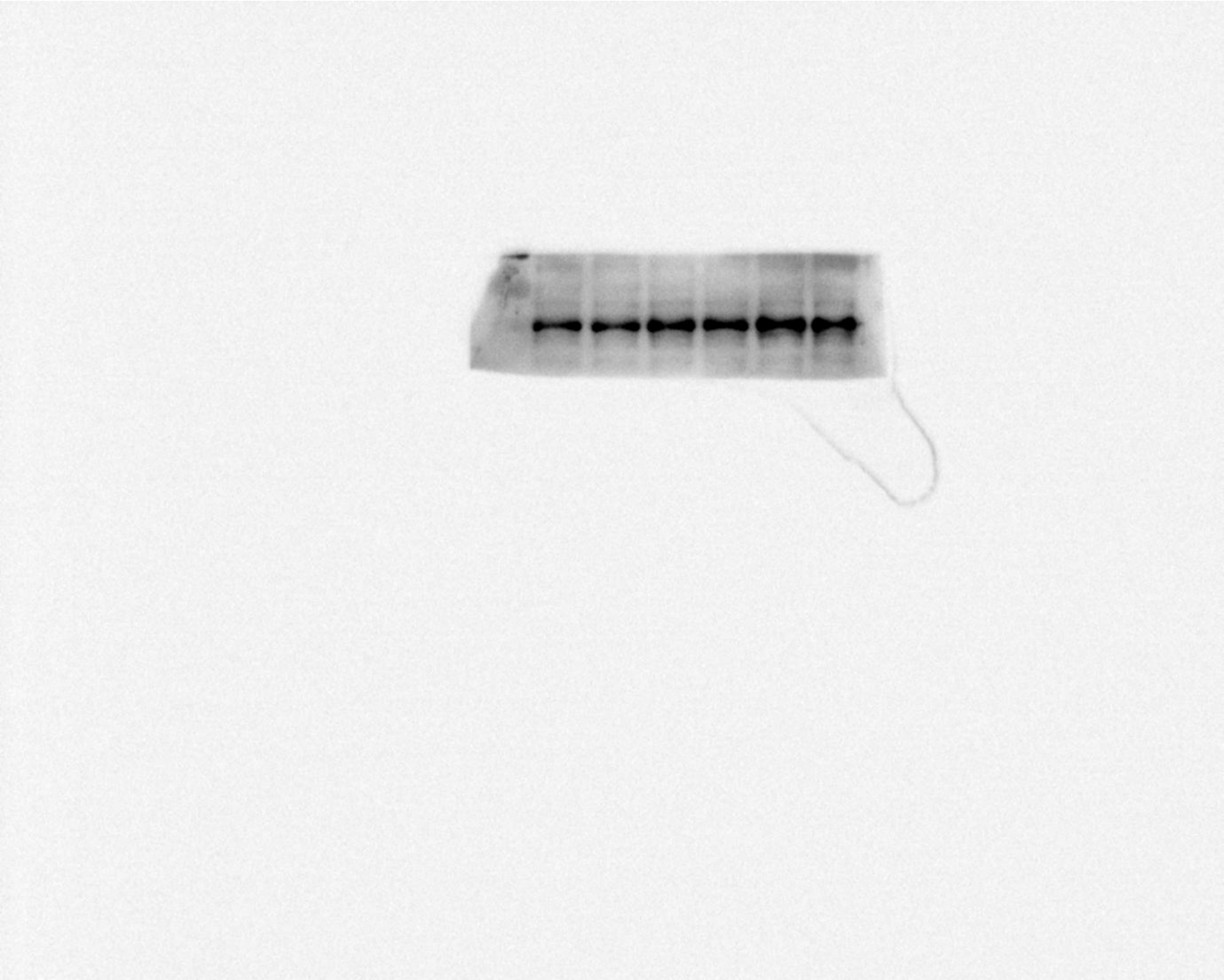

Figure 2 B

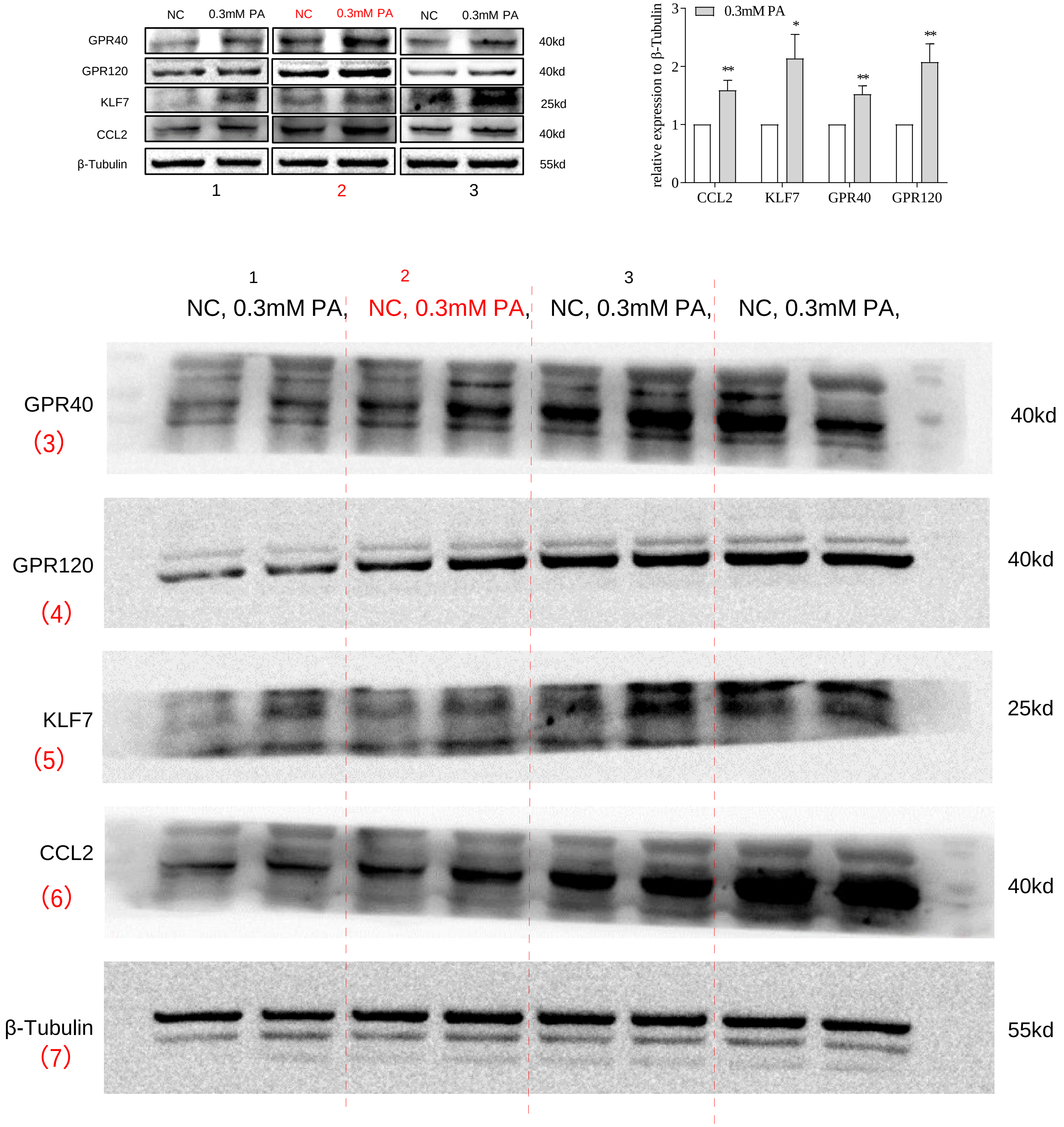
NC
0.3mM PA
GPR40
GPR120
KLF7
CCL2
β-Tubulin
40kd
40kd
25kd
40kd
55kd
1
2
3
relative expression to β-Tubulin
0
1
2
3
CCL2
KLF7
GPR40
GPR120
**
*
**
**
NC, 0.3mM PA,
NC, 0.3mM PA,
NC, 0.3mM PA,
NC, 0.3mM PA,
(3)
(4)
(5)
(6)
(7)

Original membrane for (3)

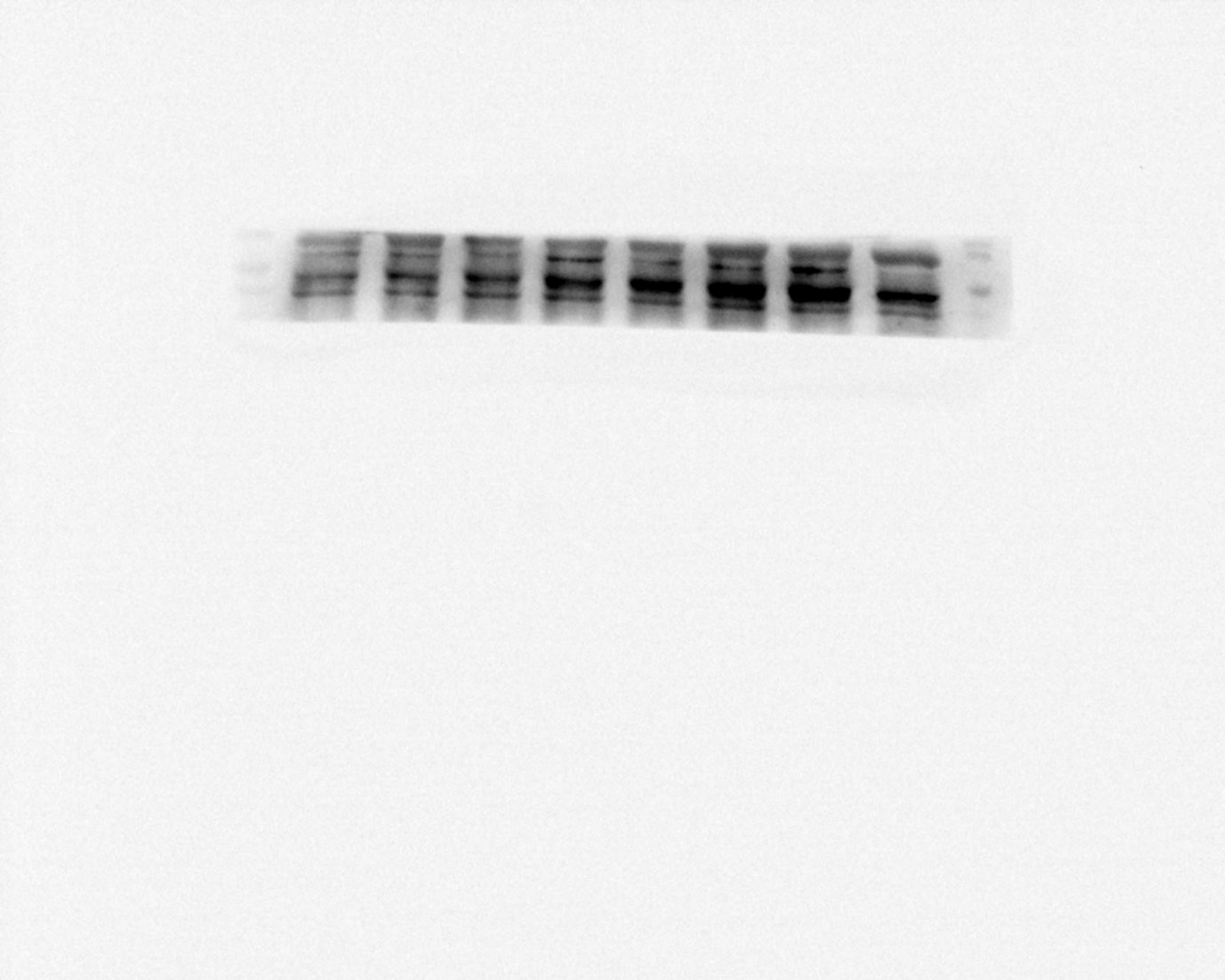

Original membrane for (4)

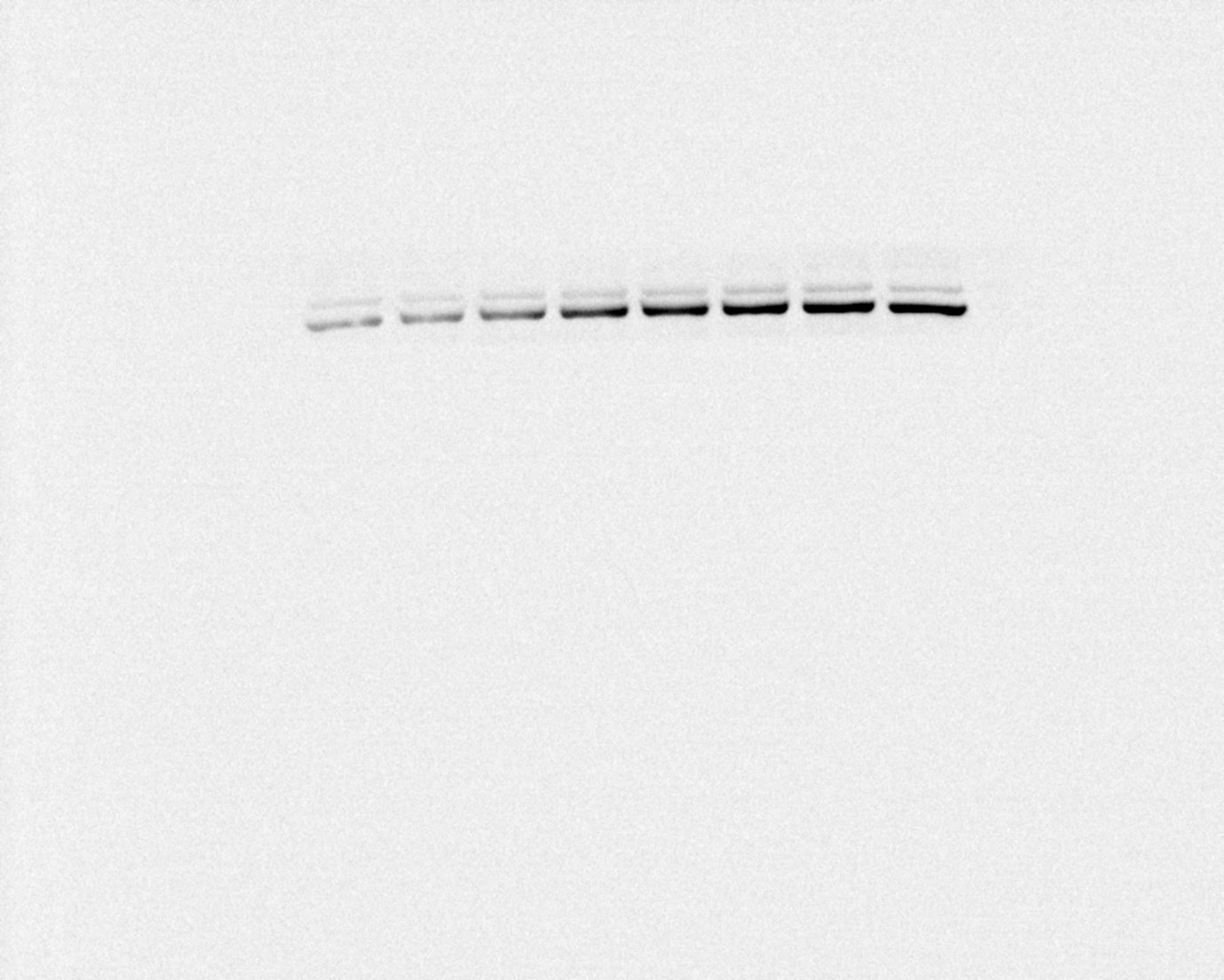

Original membrane for (5)

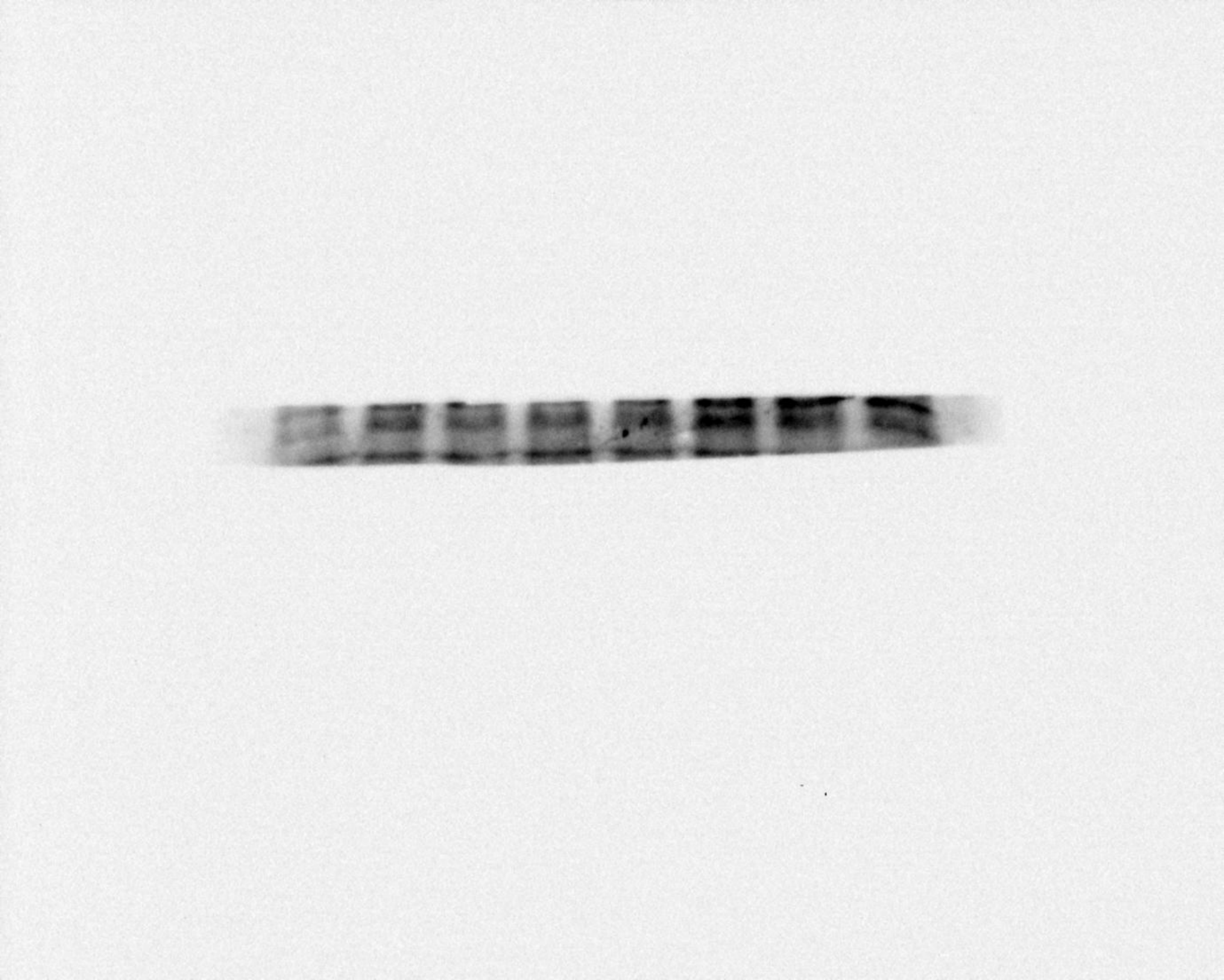

Original membrane for (6)

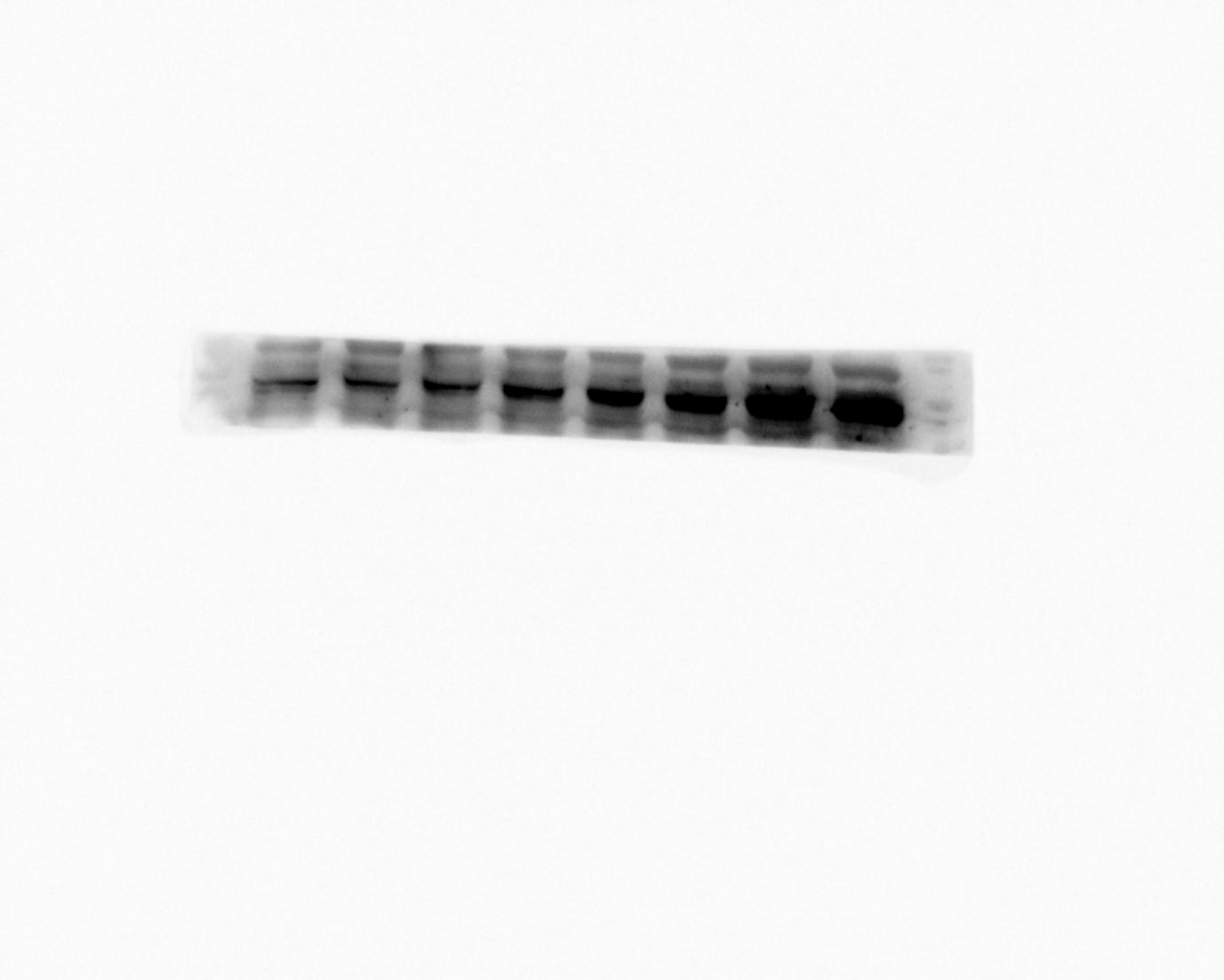

Original membrane for (7)

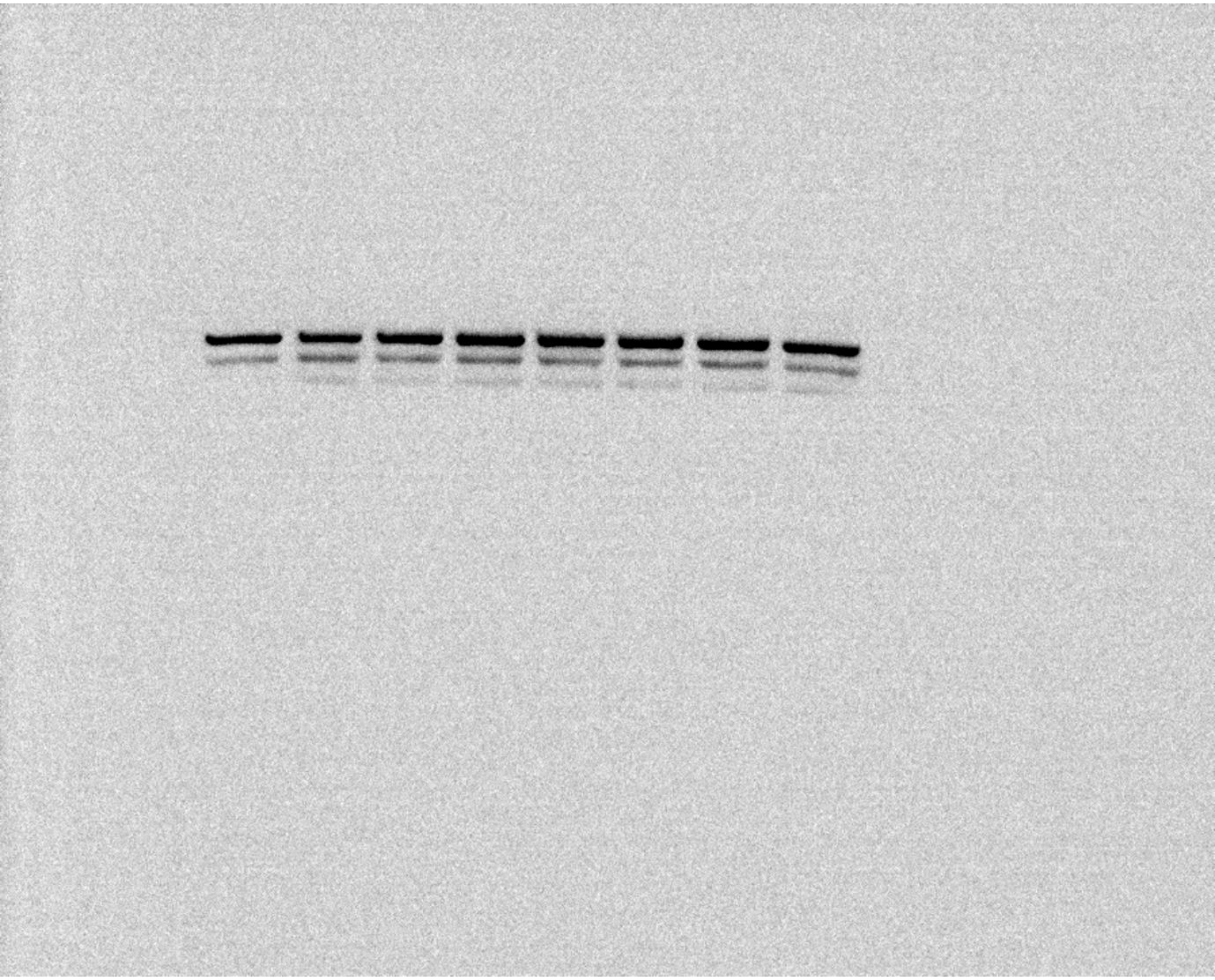

Figure 3 B

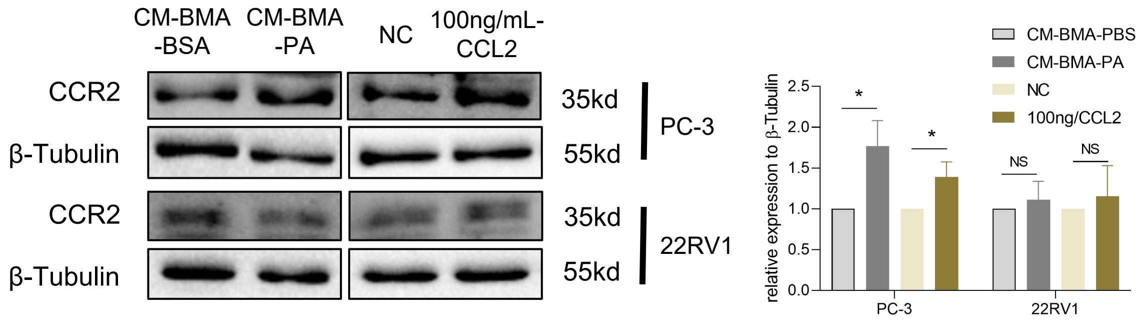

PC-3 cells

CM-BMA-BSA *vs.* CM-BMA-PA

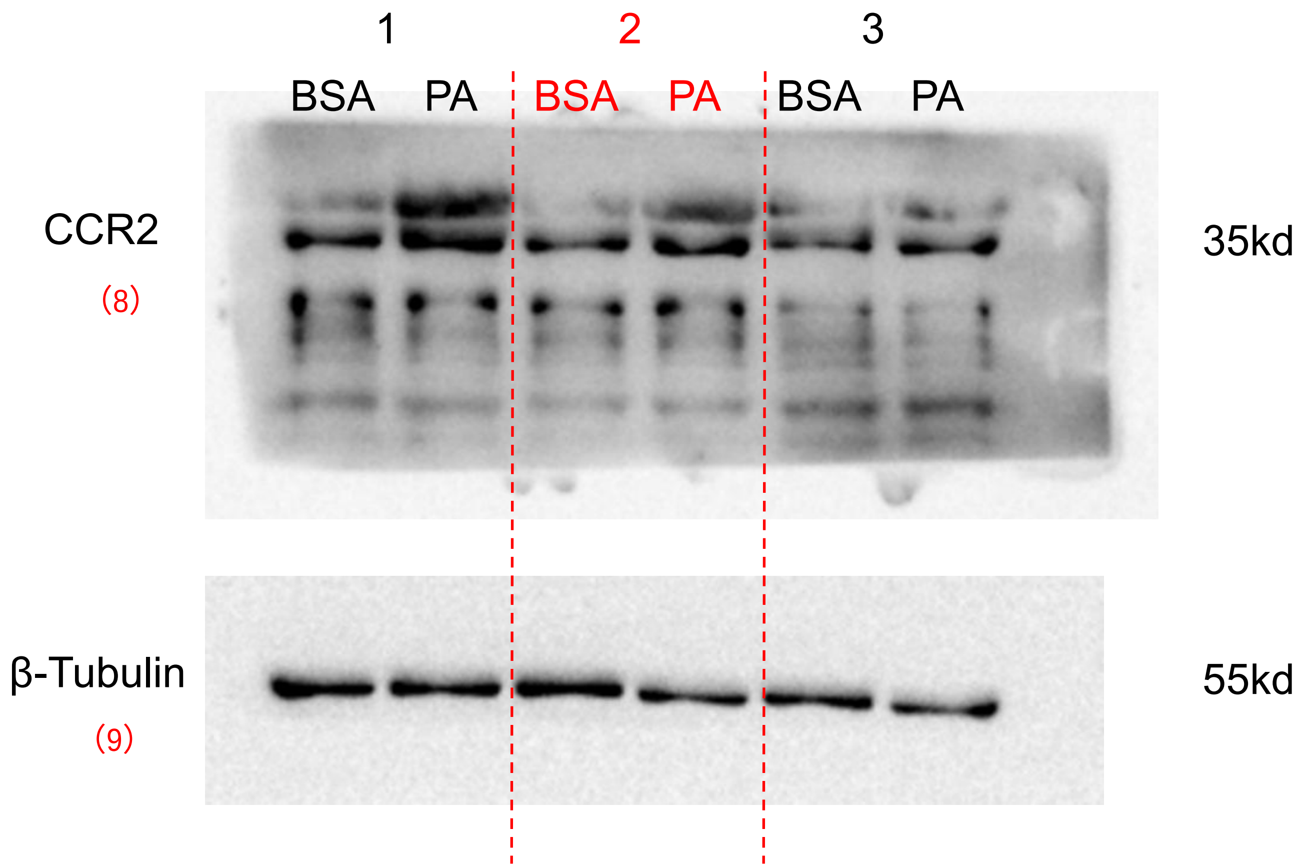

PC-3 cells

NC *vs.* 100ng/mL CCL2

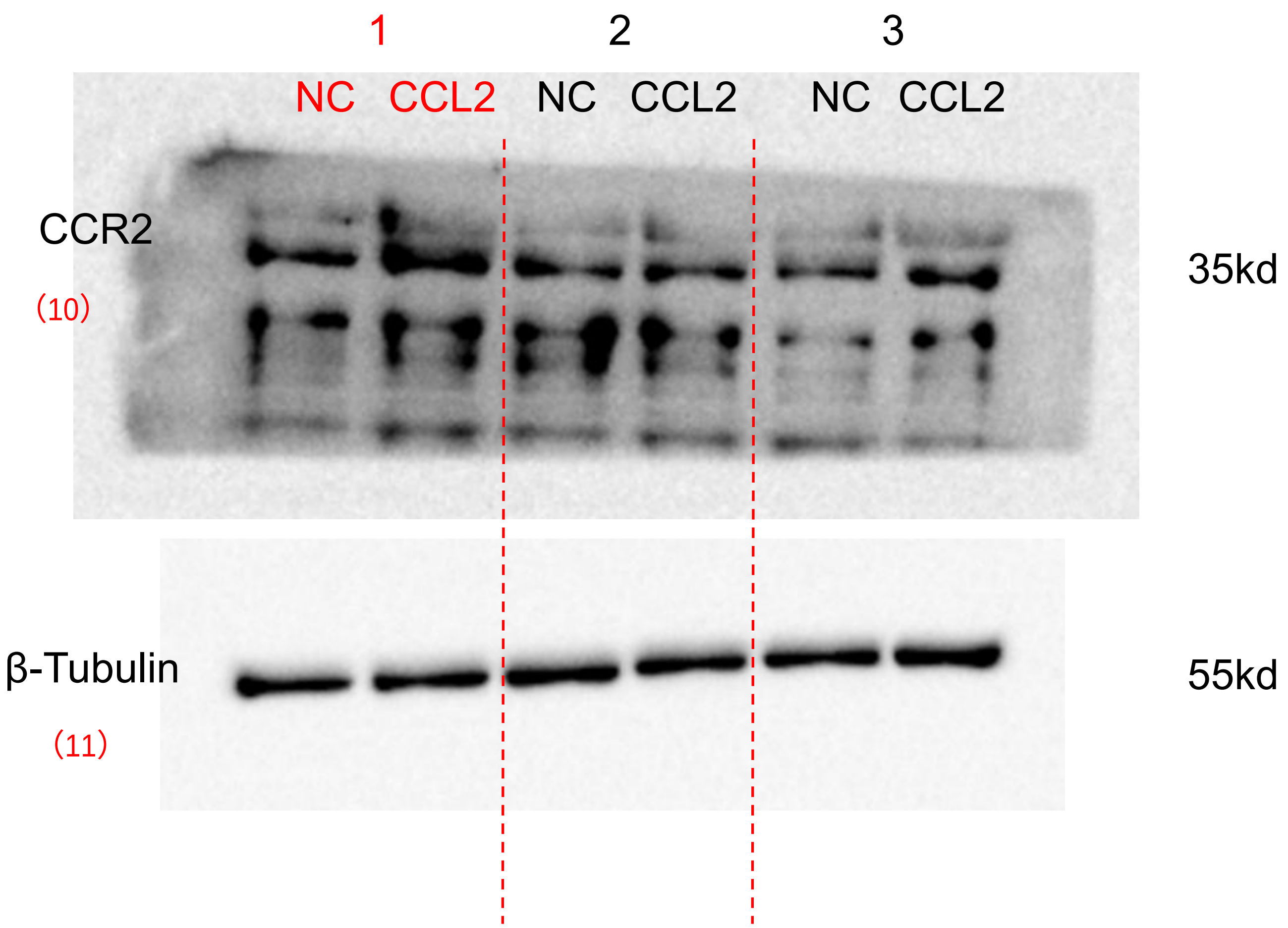

22RV1 cells

CM-BMA-BSA *vs.* CM-BMA-PA

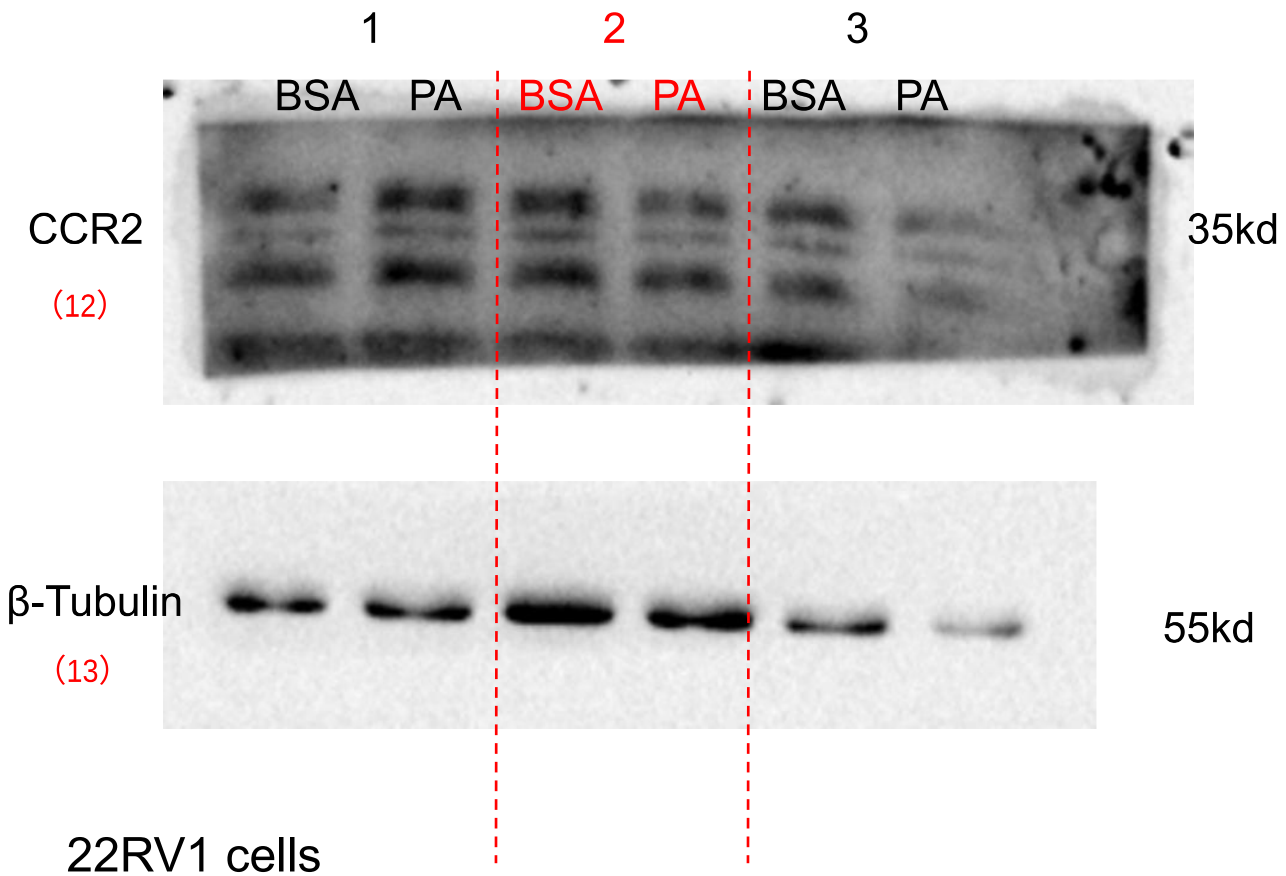

22RV1 cells

NC *vs.* 100ng/mL CCL2

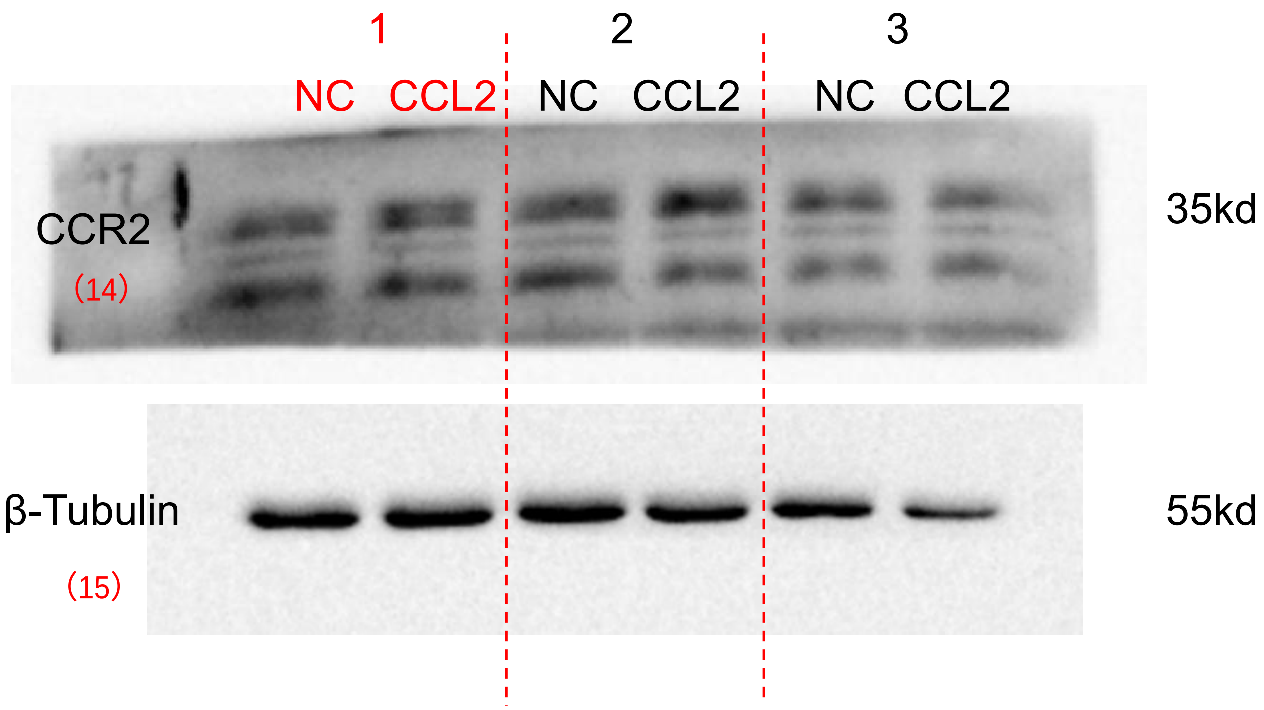

Original membrane for (8)

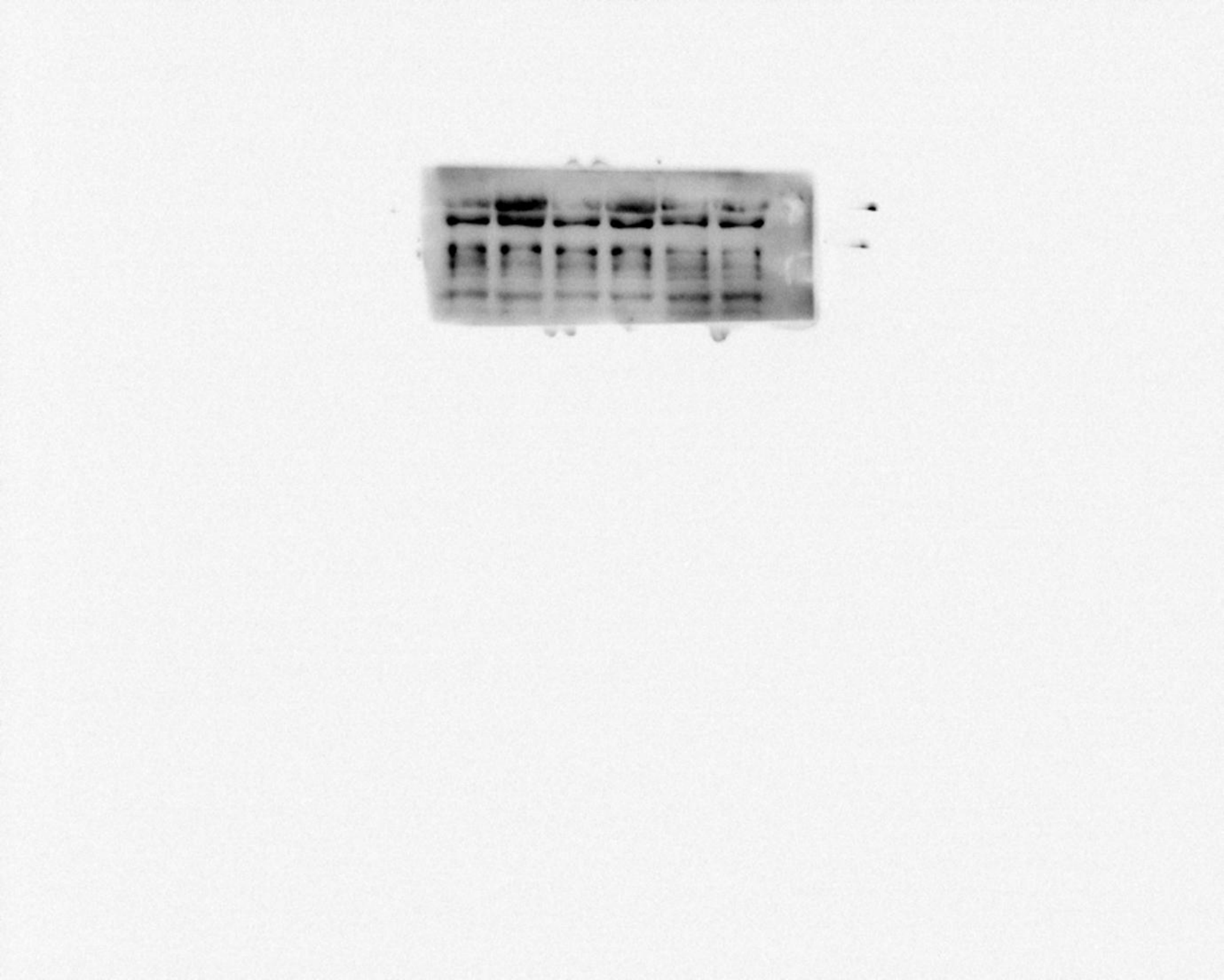

Original membrane for (9)

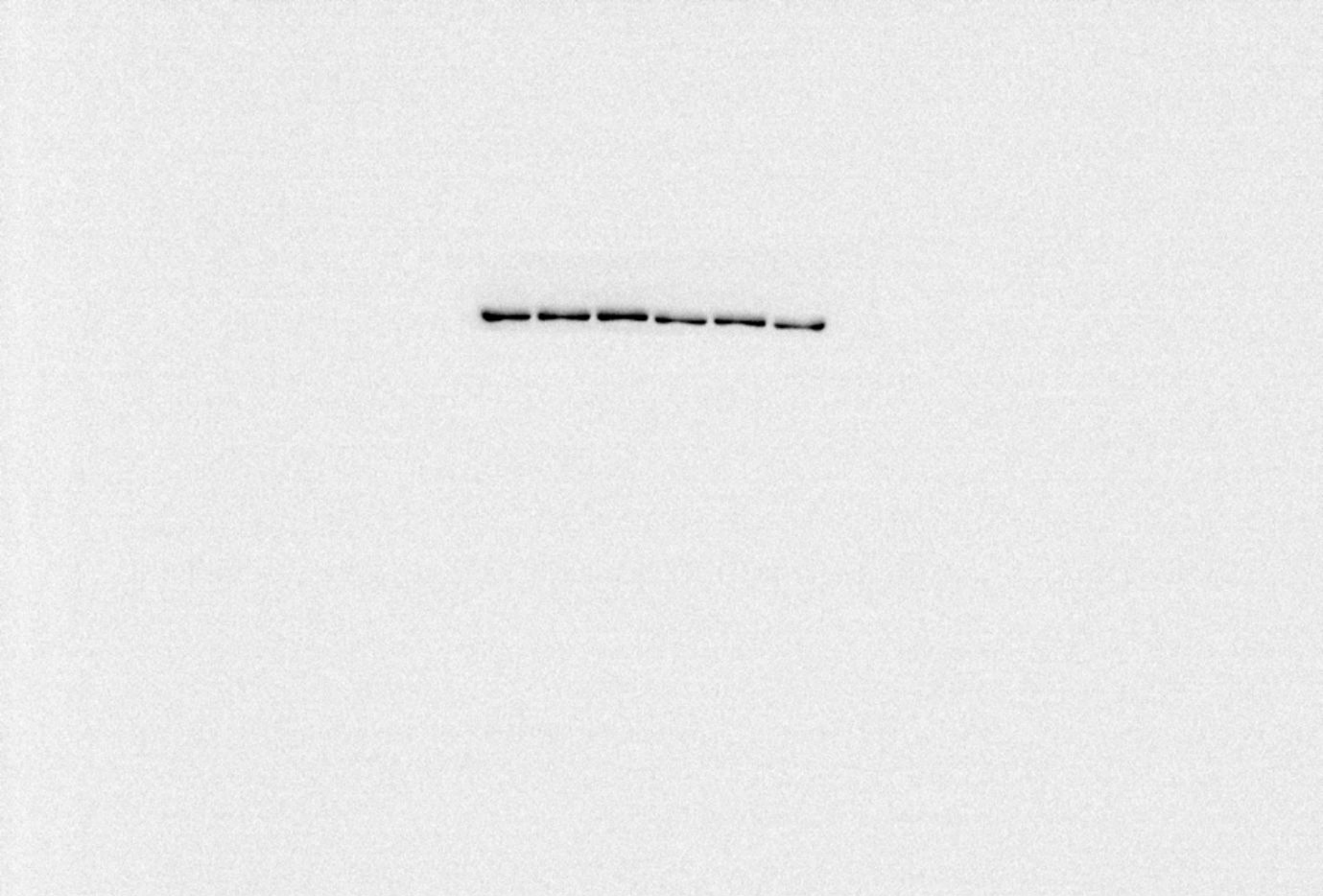

Original membrane for (10)

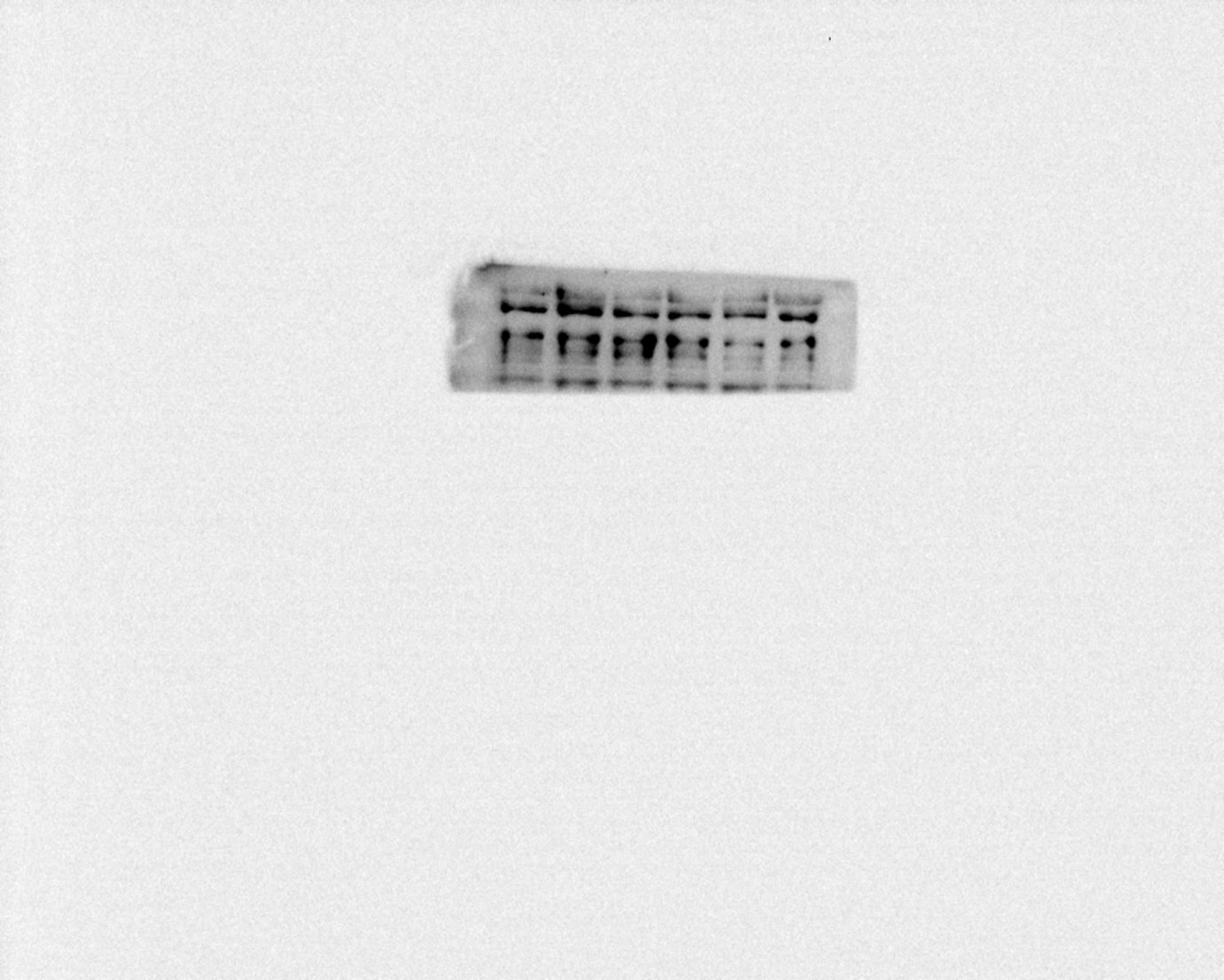

Original membrane for (11)

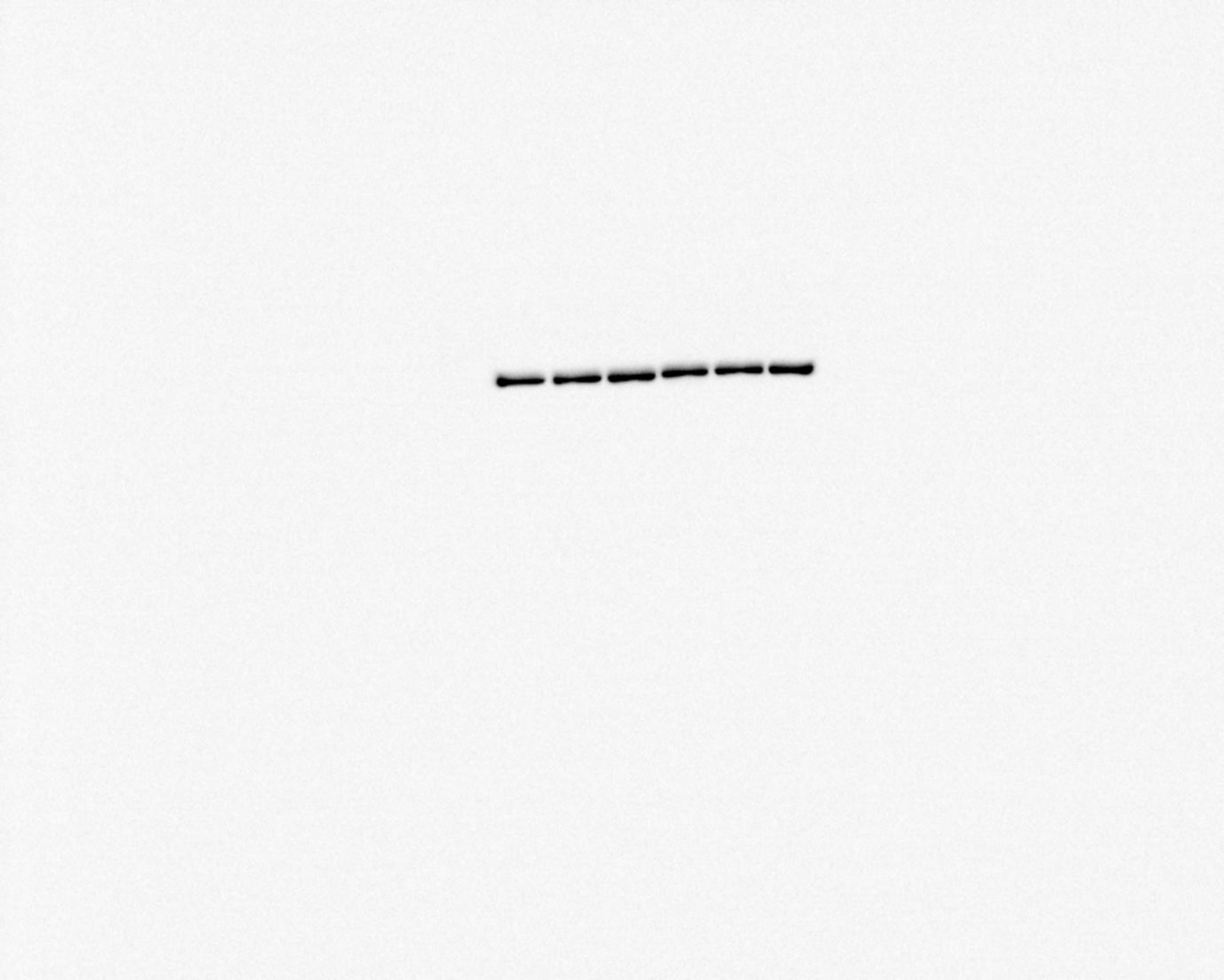

Original membrane for (12)

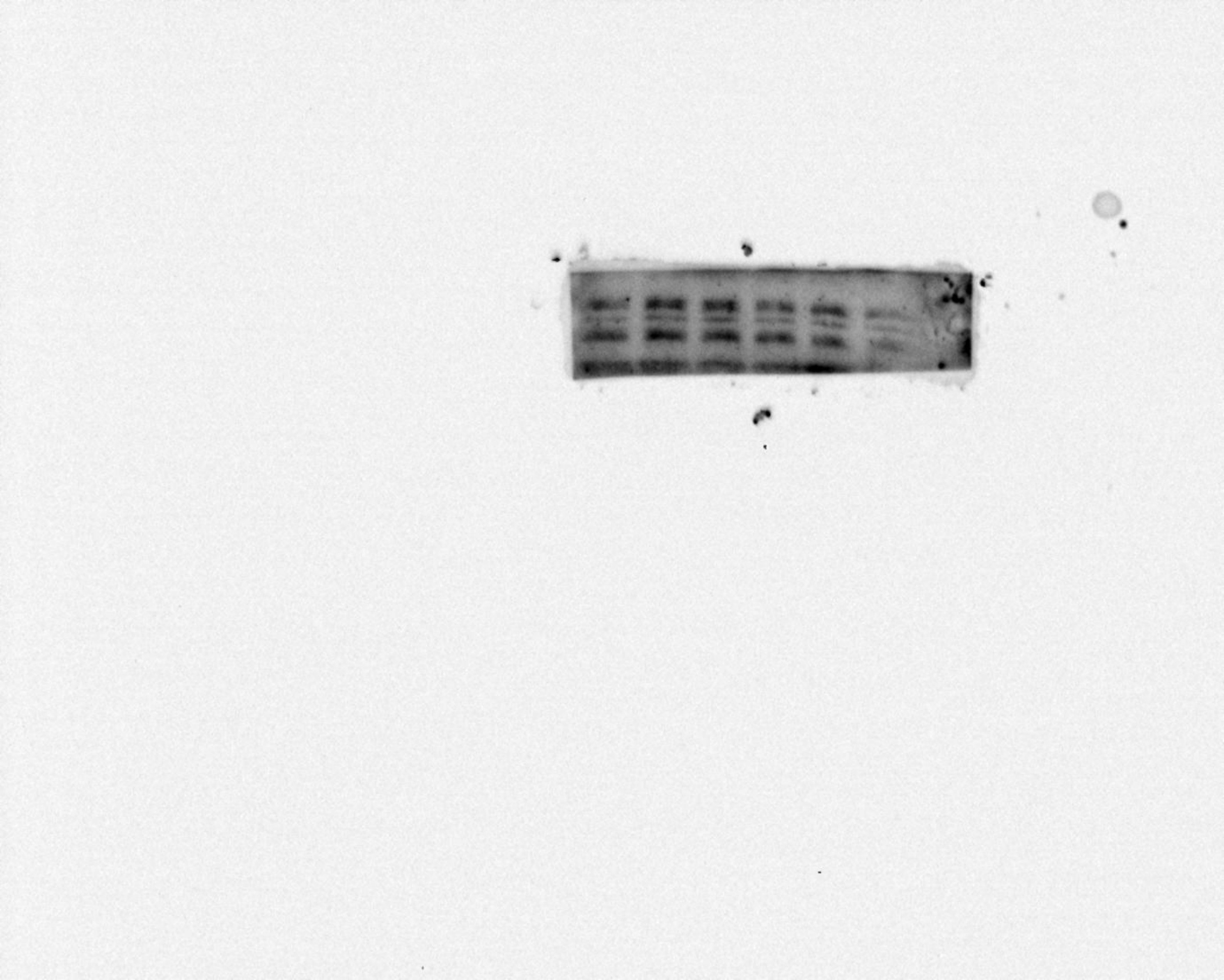

Original membrane for (13)

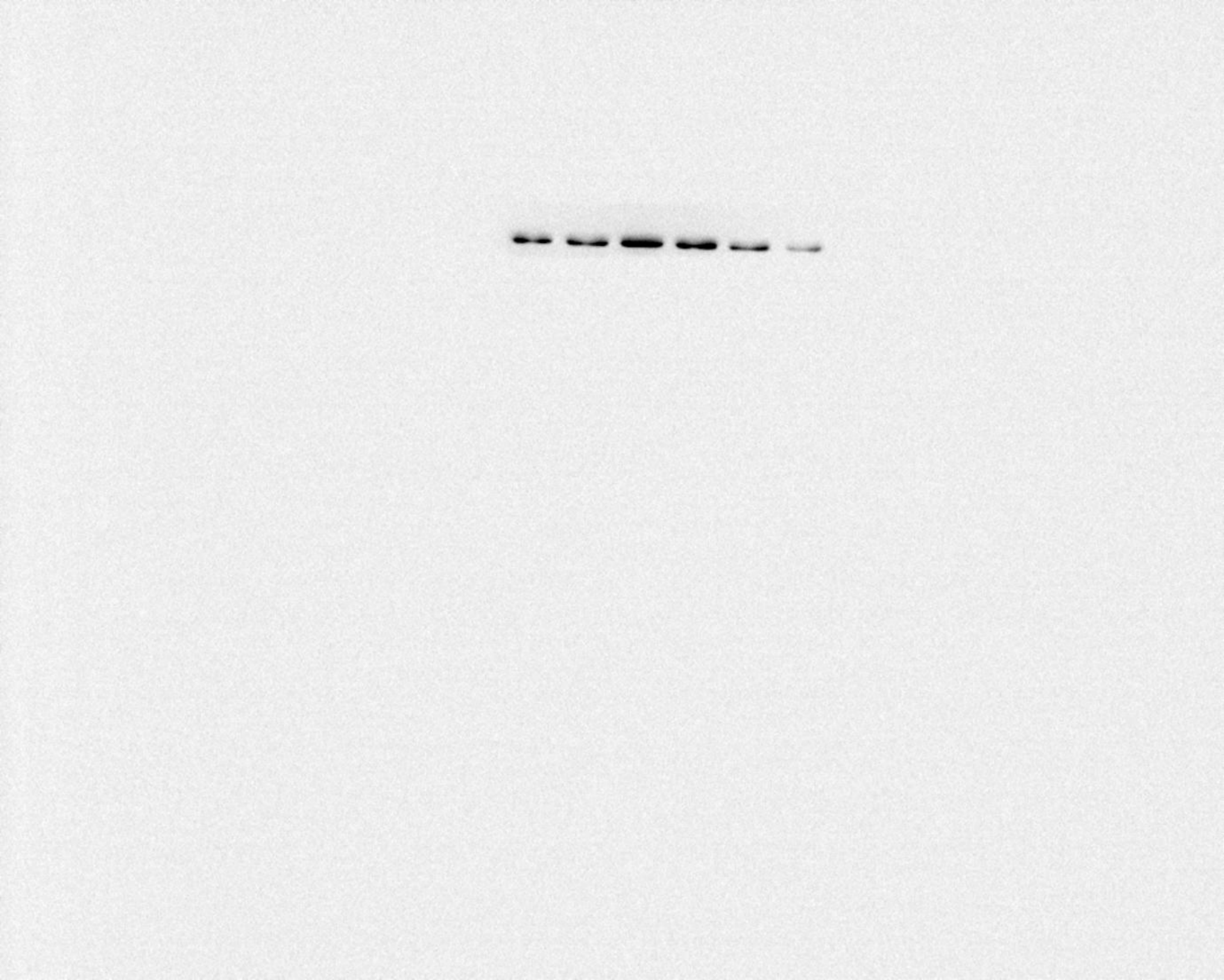

Original membrane for (14)

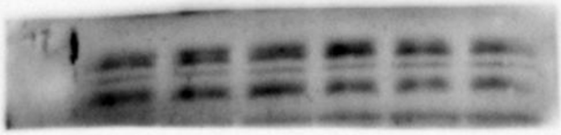

Original membrane for (15)

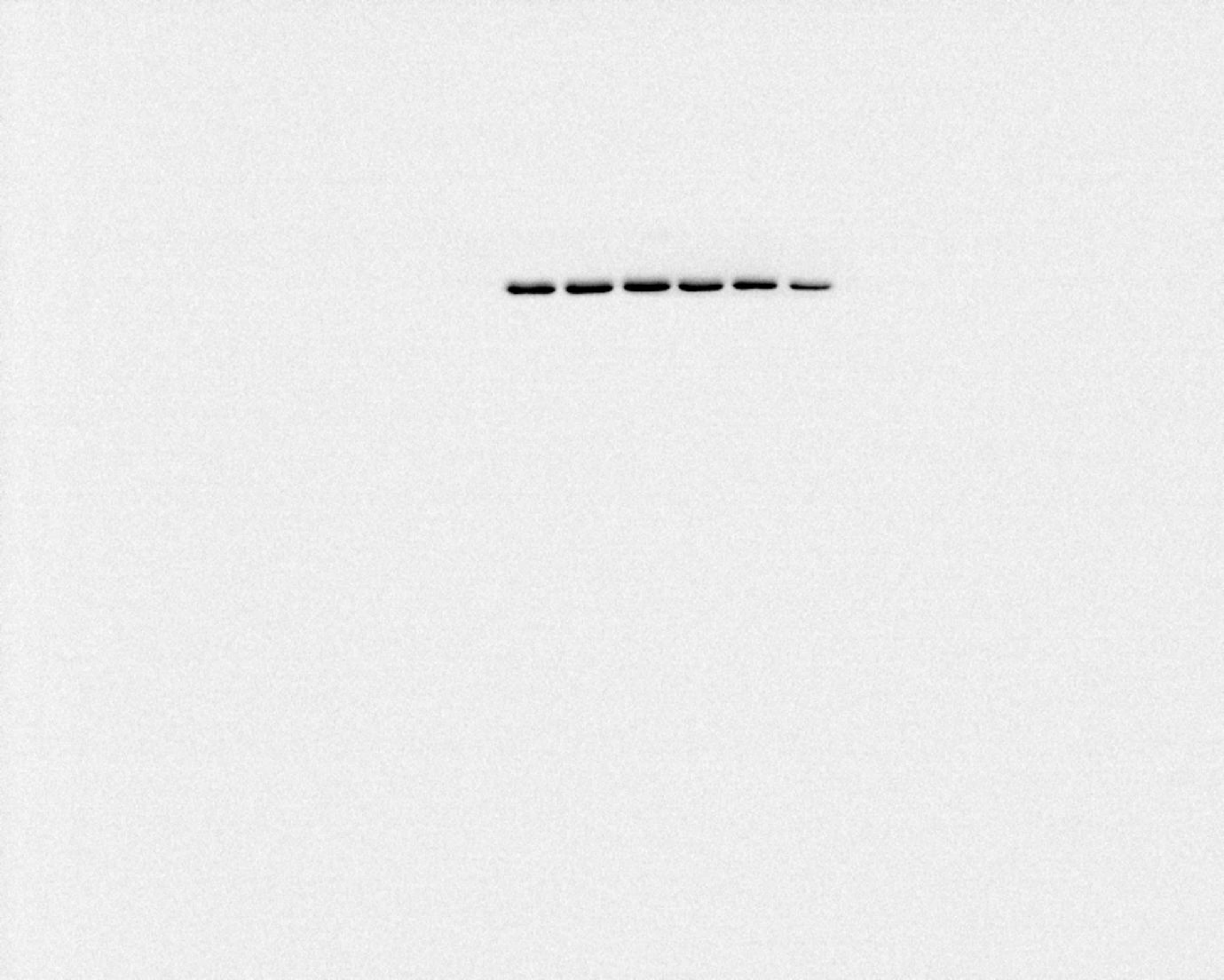

Figure 3 H

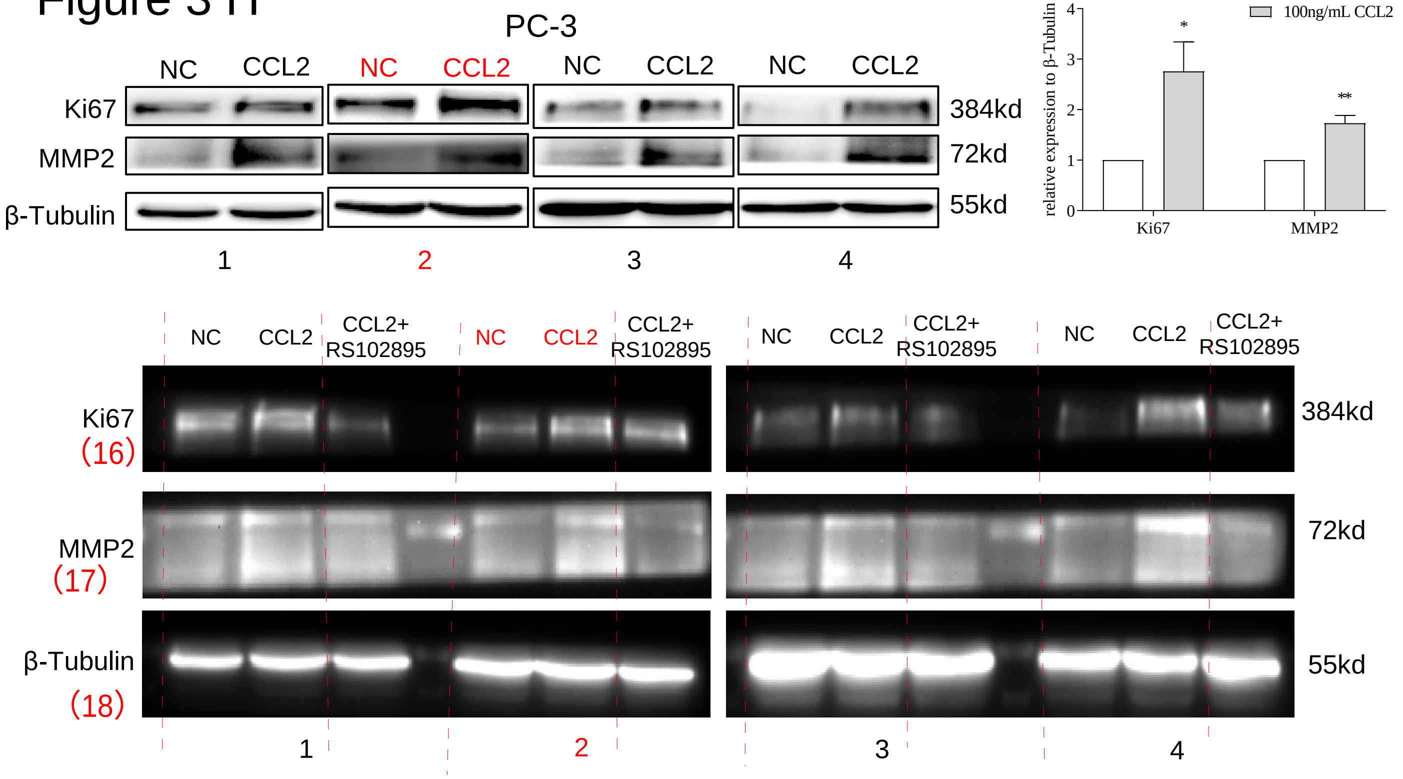

Figure 3 N

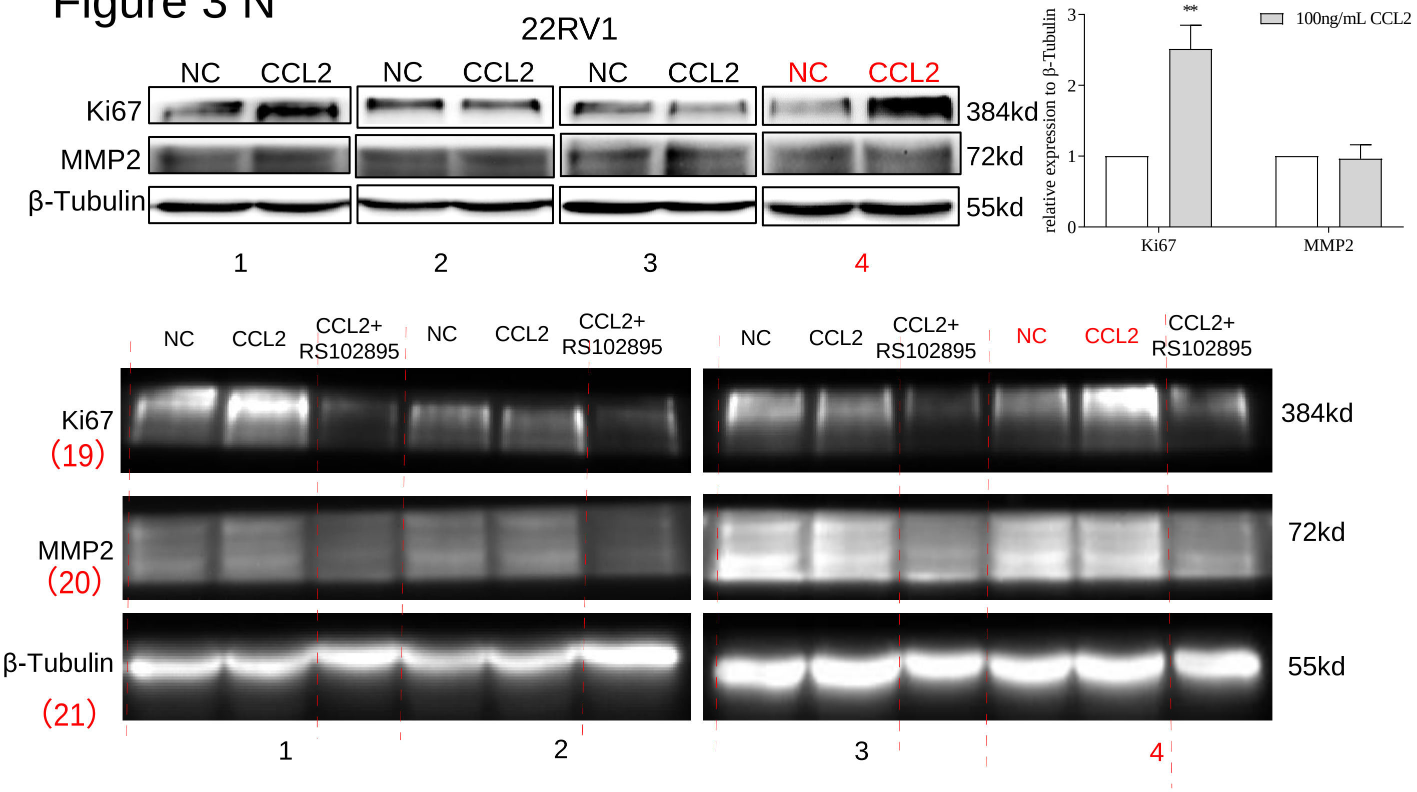

Original membrane for (16)

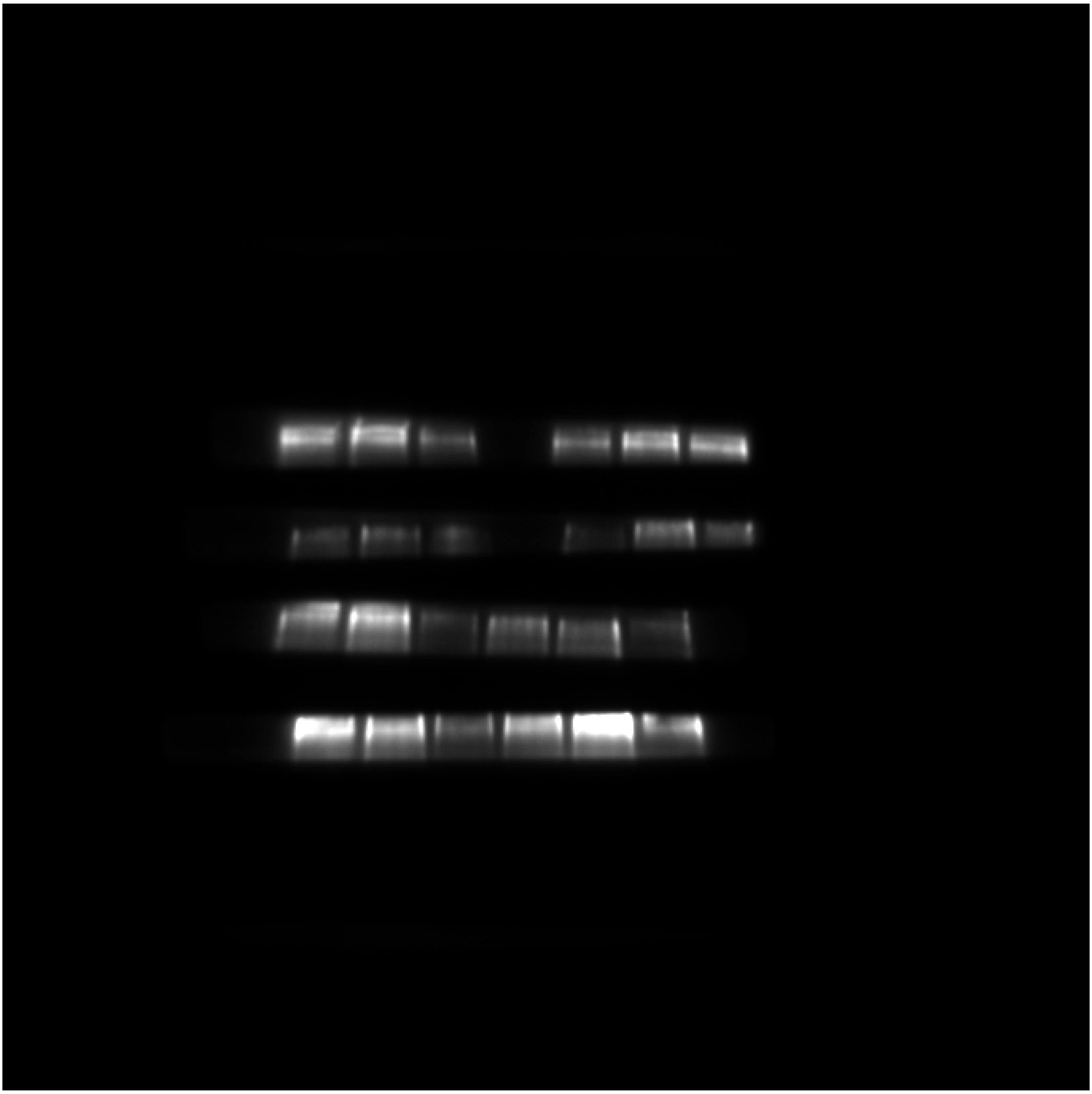

Original membrane for (17)

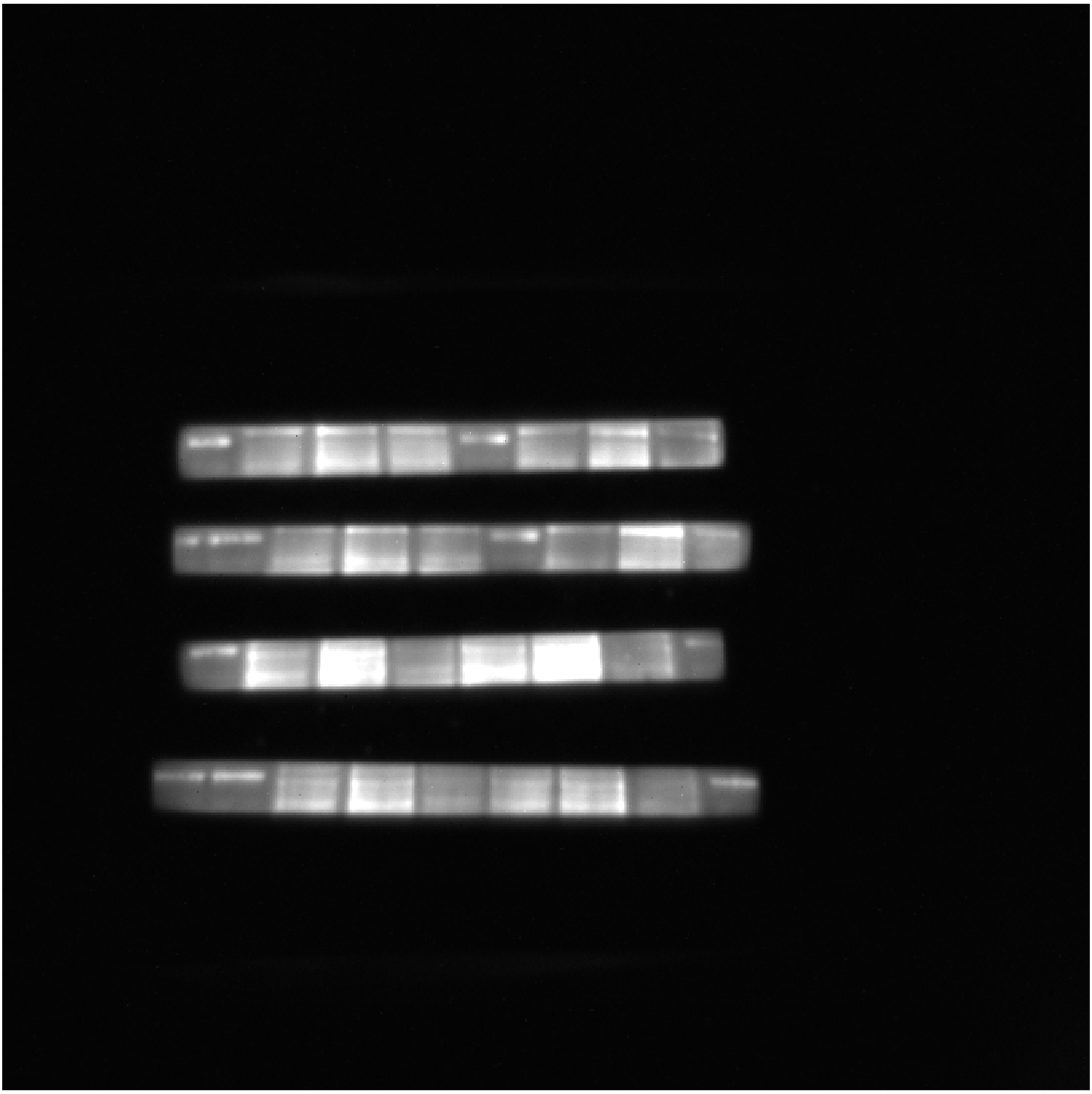

Original membrane for (18)

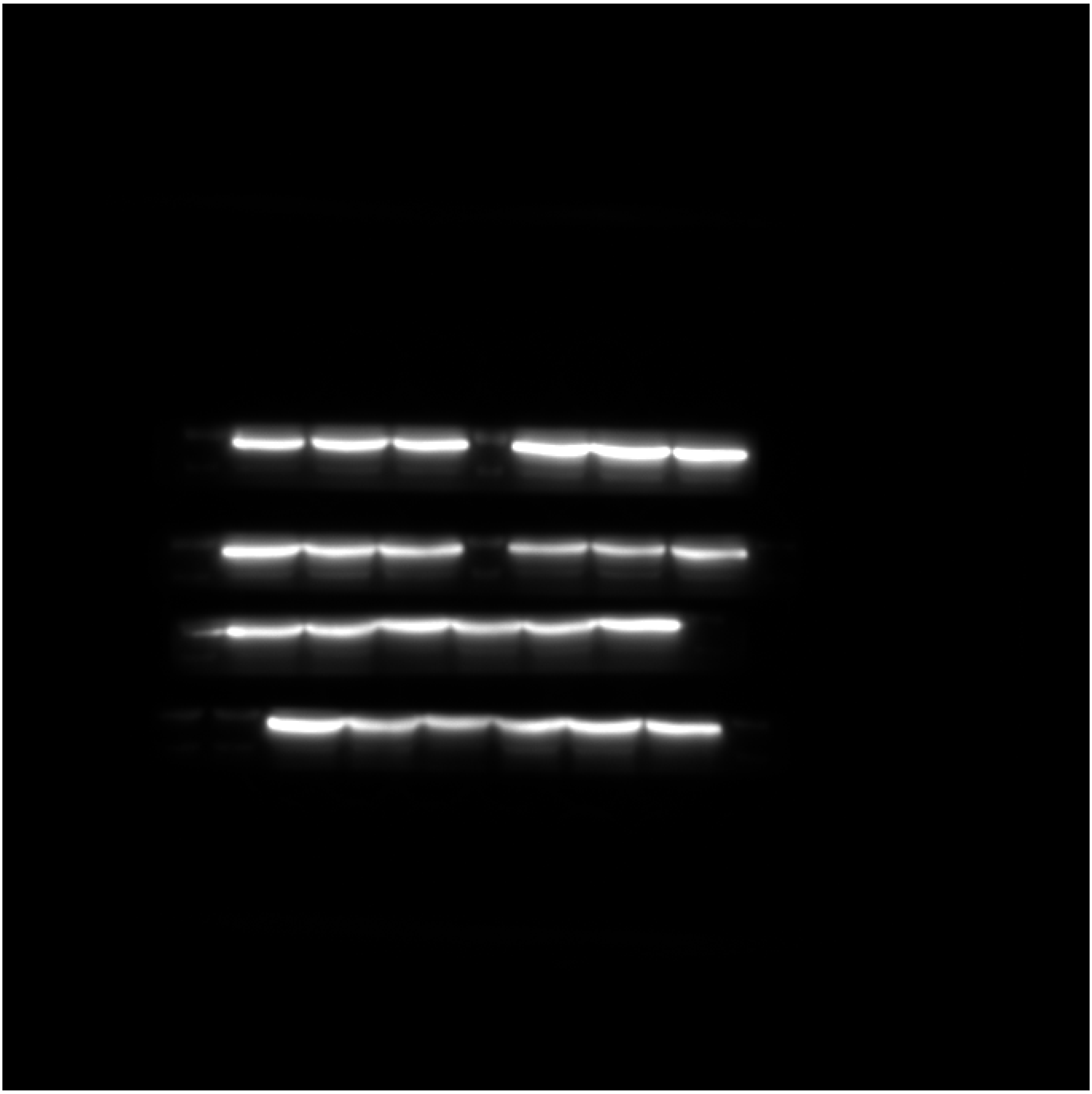

Original membrane for (18)

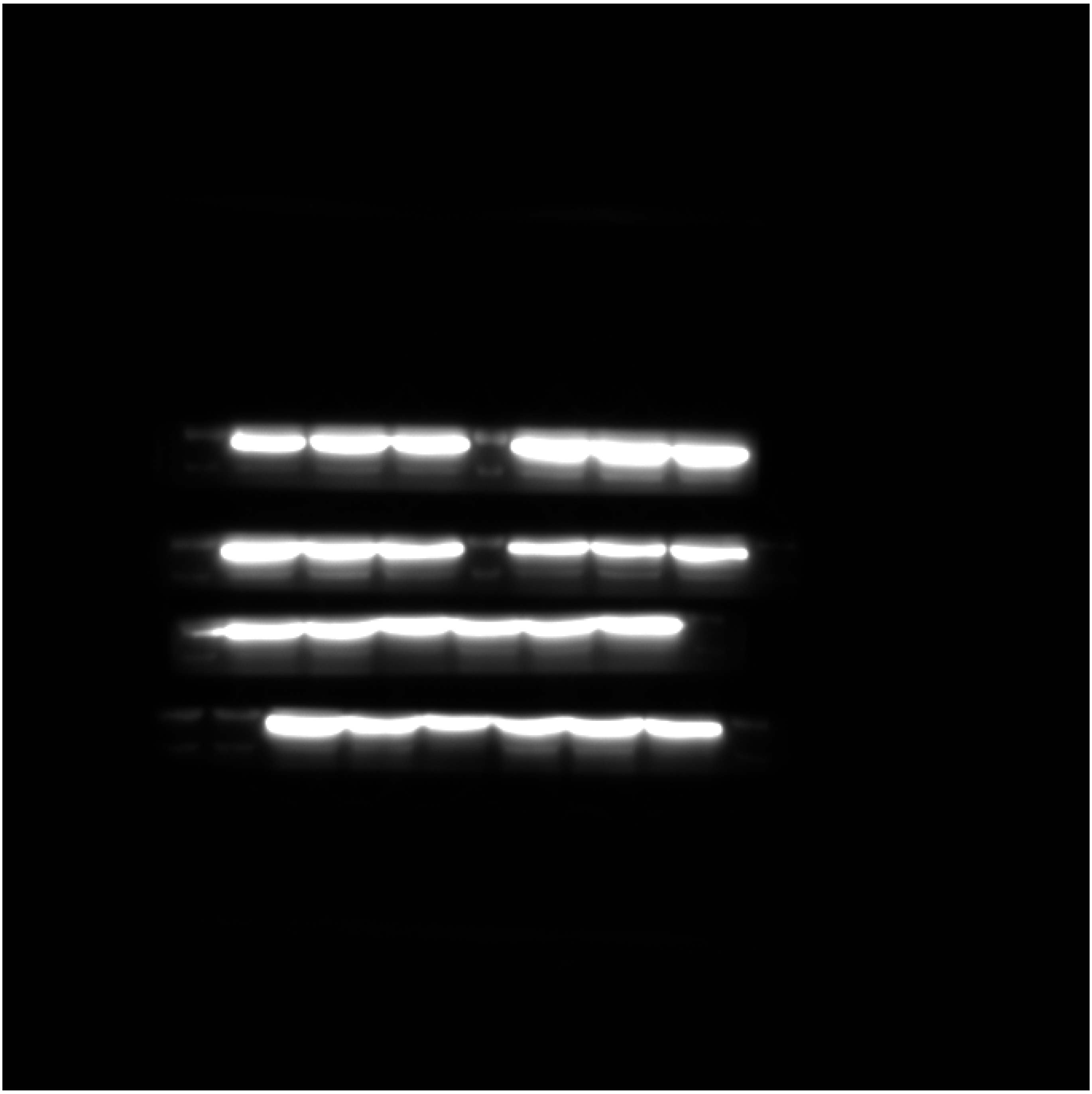

Original membrane for (19)

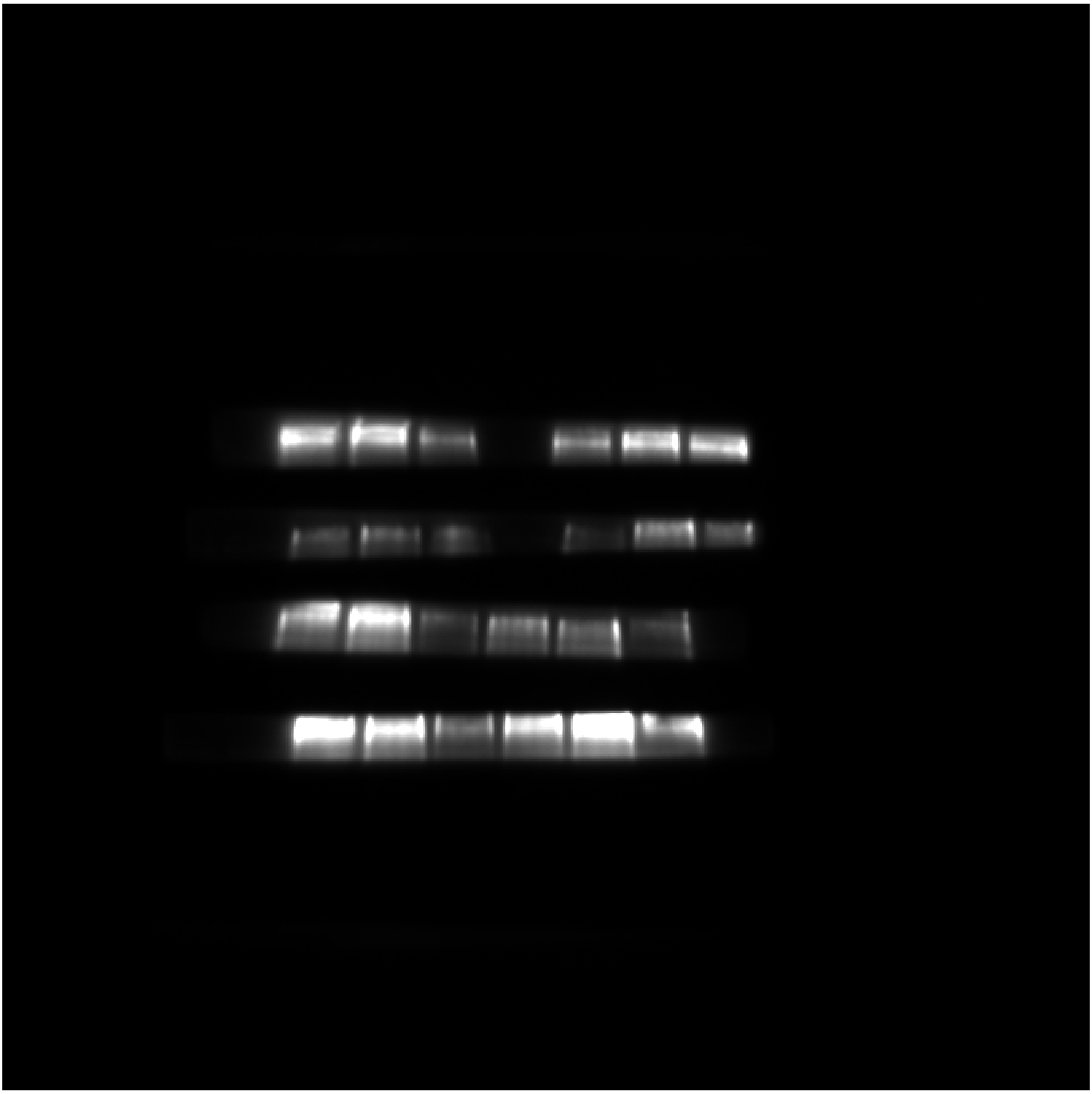

Original membrane for (19)

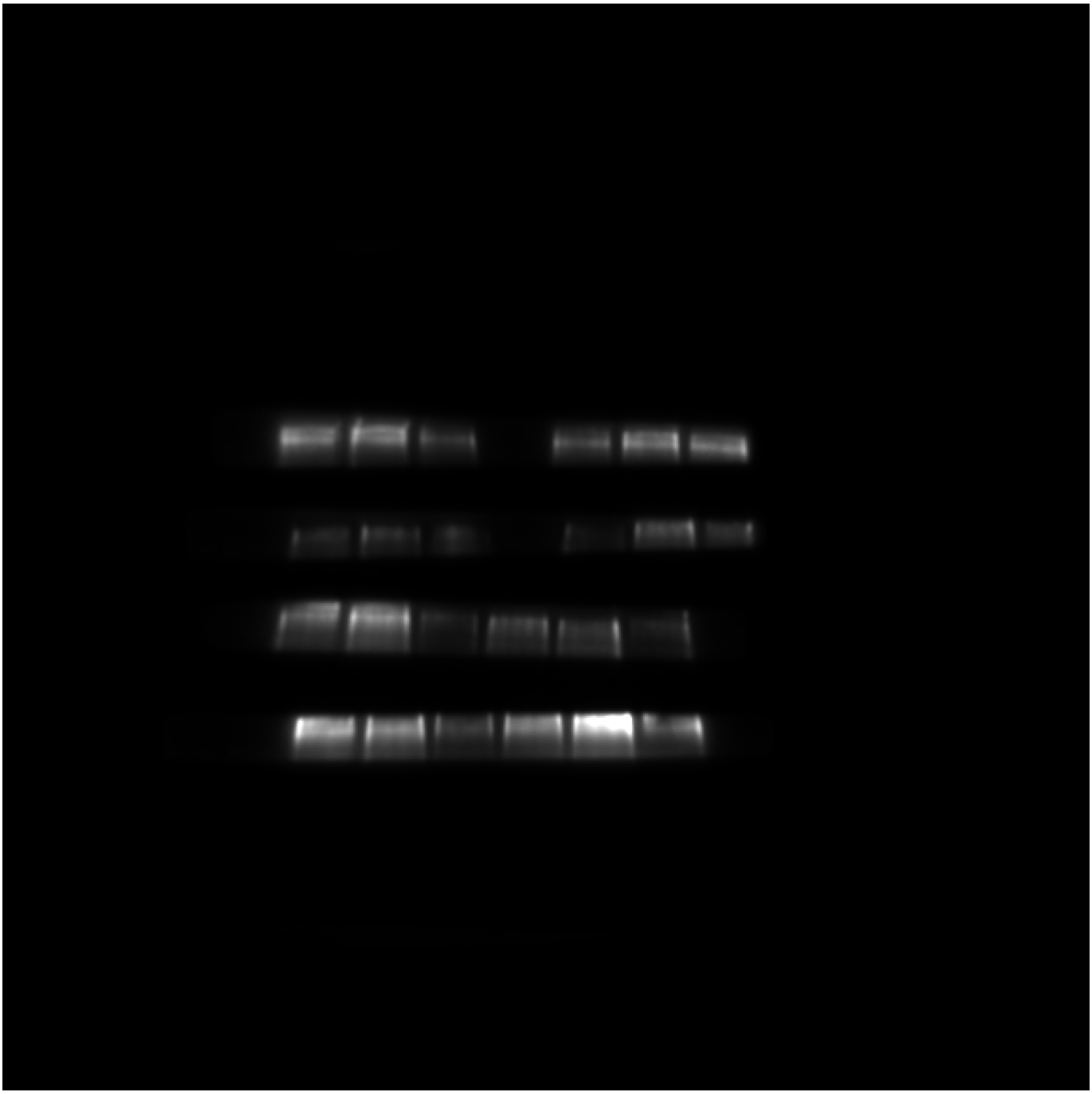

Original membrane for (20)

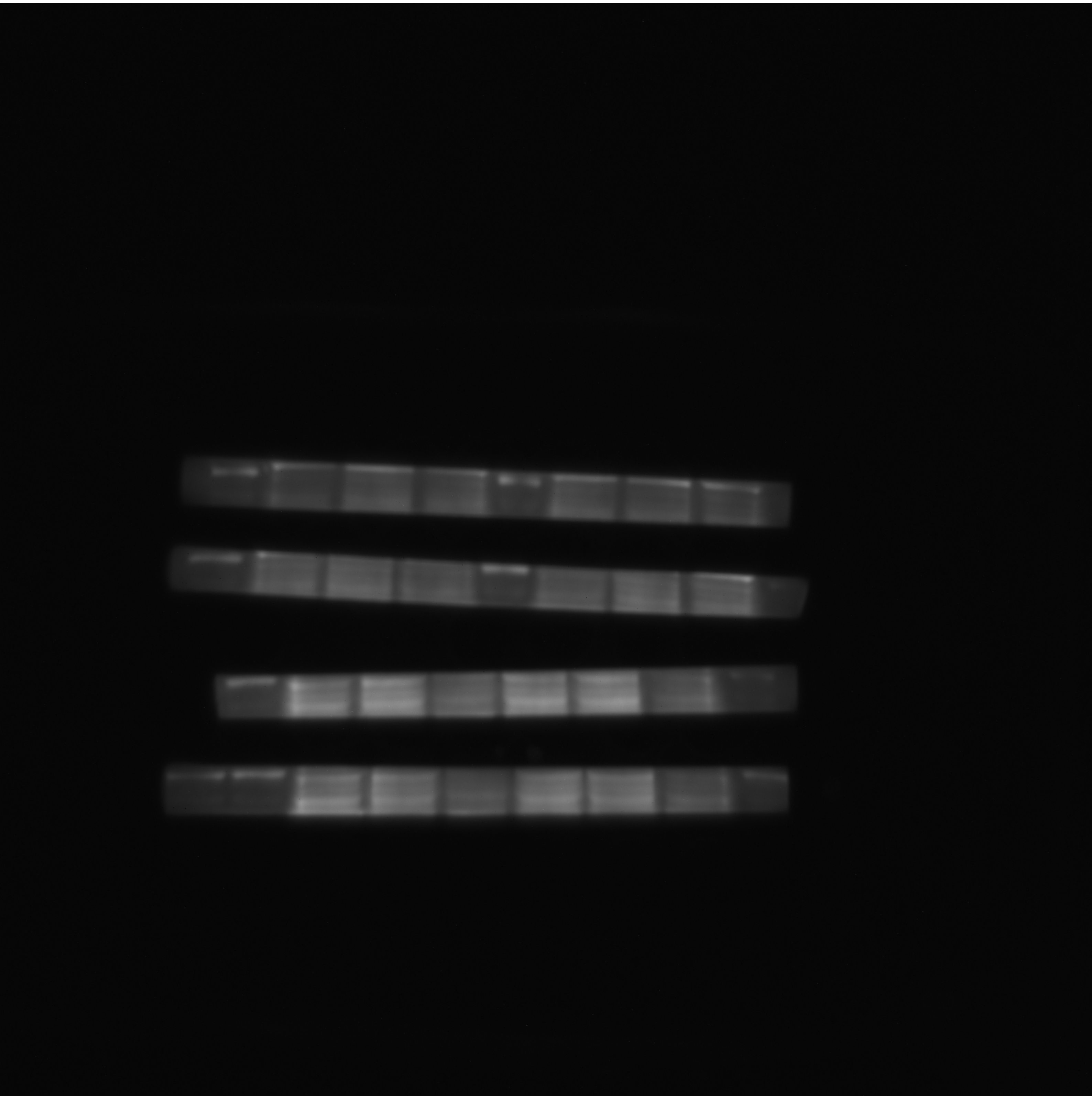

Original membrane for (20)

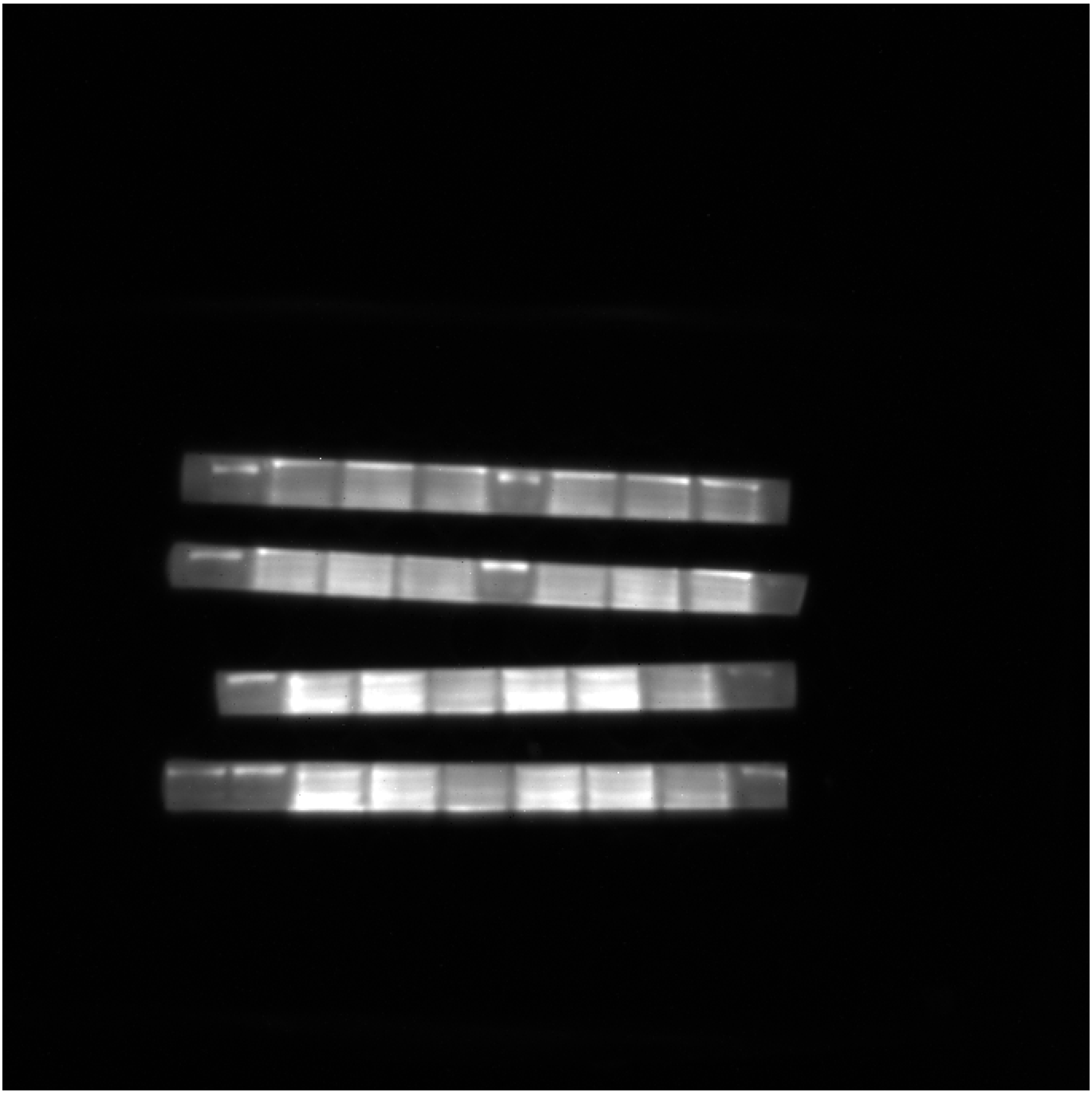

Original membrane for (21)

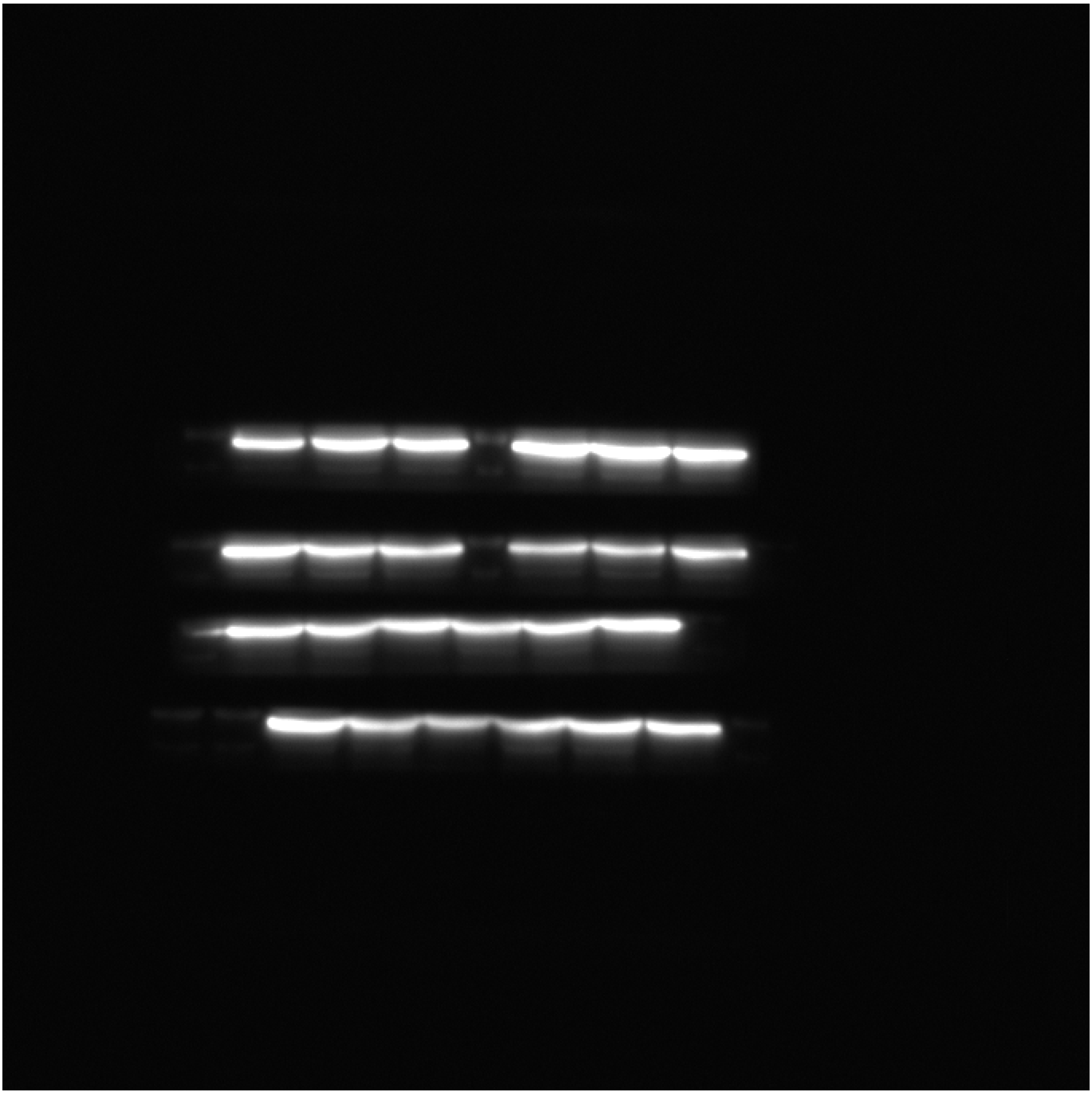

Original membrane for (21)

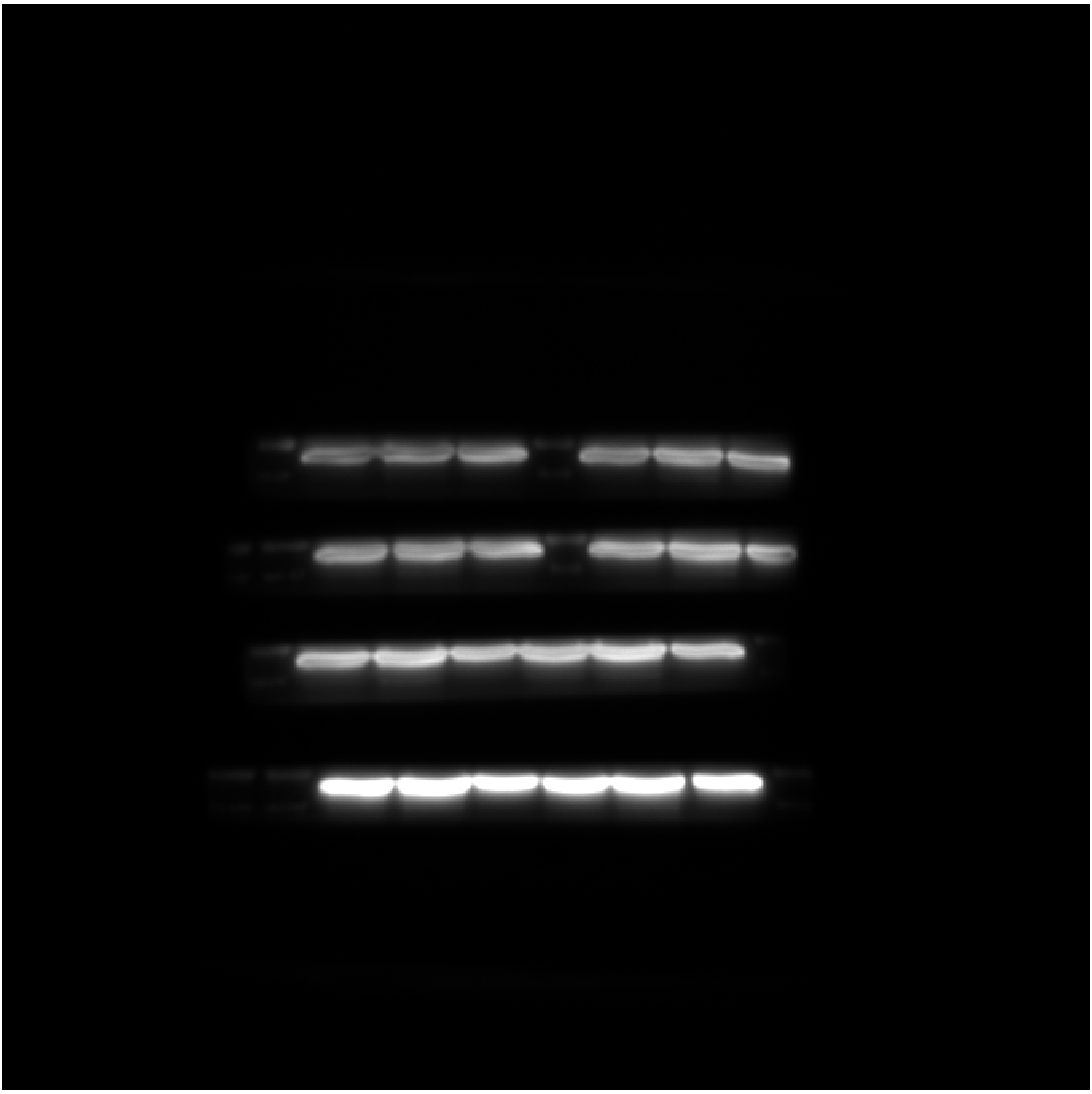

Figure 5 C

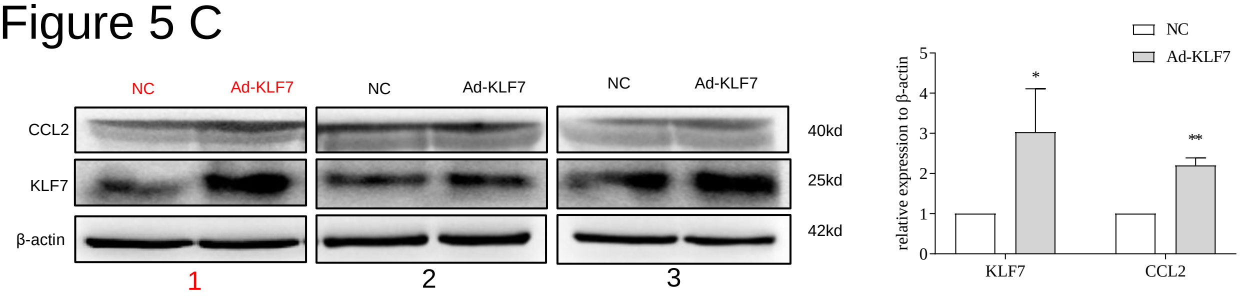
NC
Ad-KLF7
NC
Ad-KLF7
NC
Ad-KLF7
CCL2
KLF7
β-actin
40kd
25kd
42kd
1
2
3
relative expression to β-actin
NC
Ad-KLF7
KLF7
CCL2
*
**

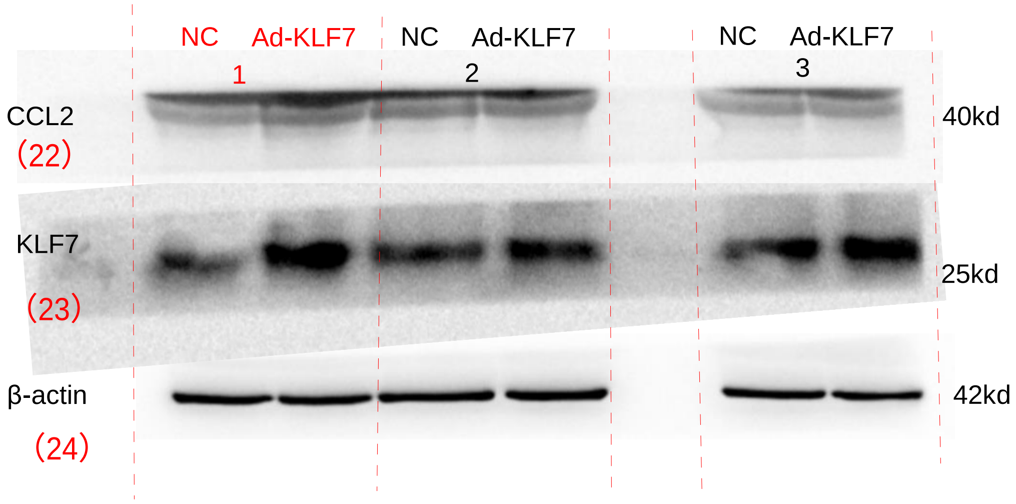
NC
Ad-KLF7
NC
Ad-KLF7
NC
Ad-KLF7
1
2
3
CCL2
(22)
KLF7
(23)
β-actin
(24)
40kd
25kd
42kd

Original membrane for (22)

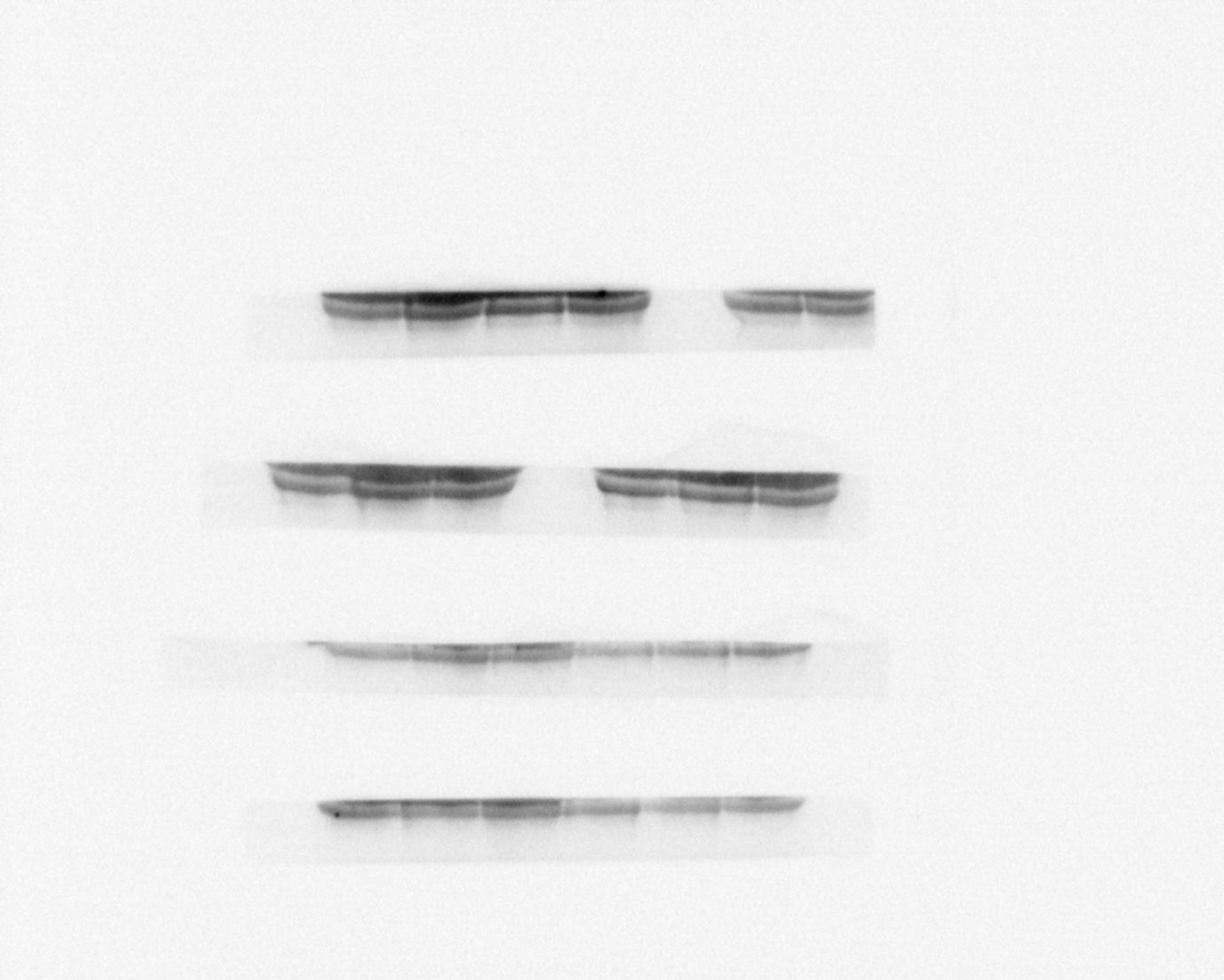

Original membrane for (23)

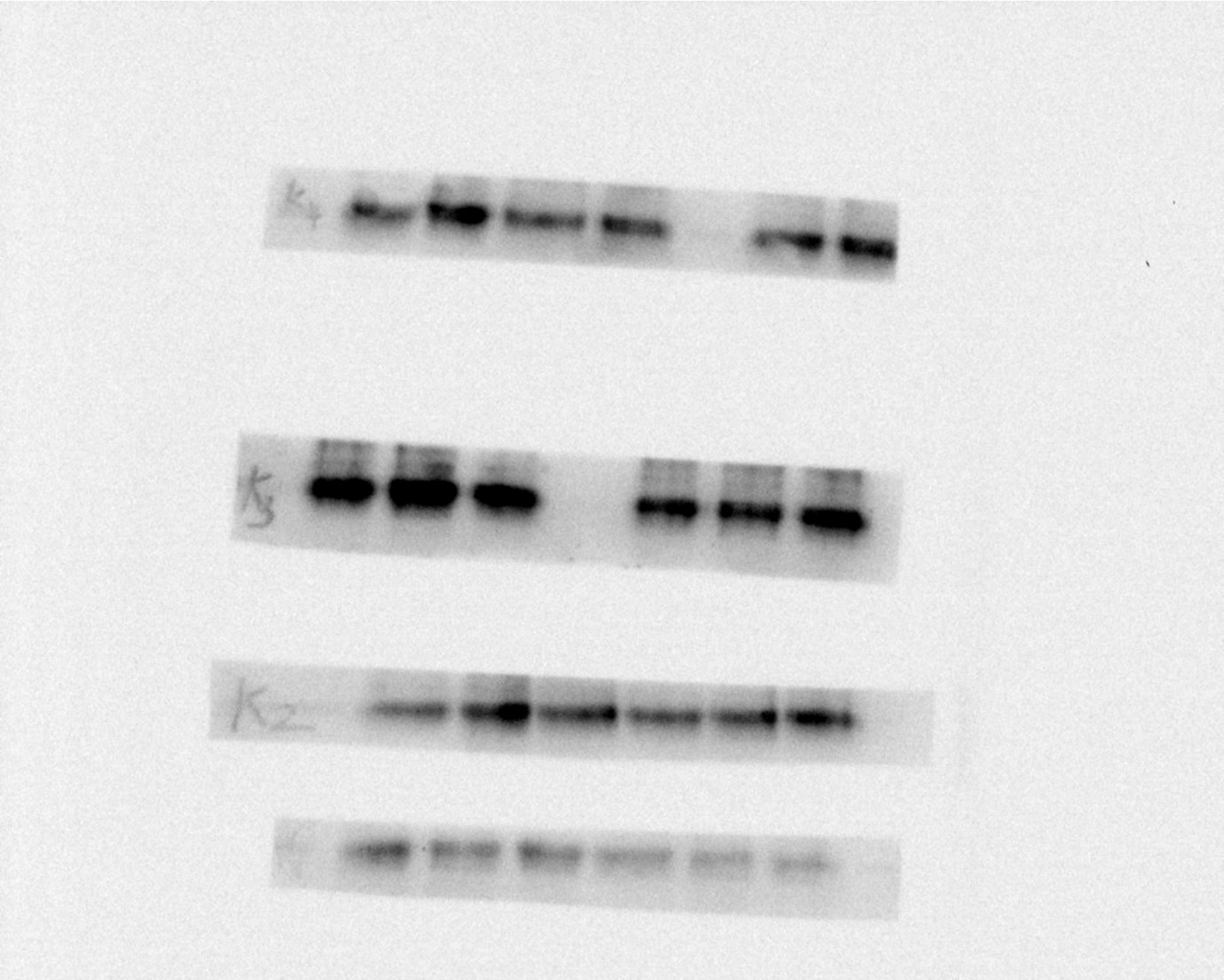

Original membrane for (24)

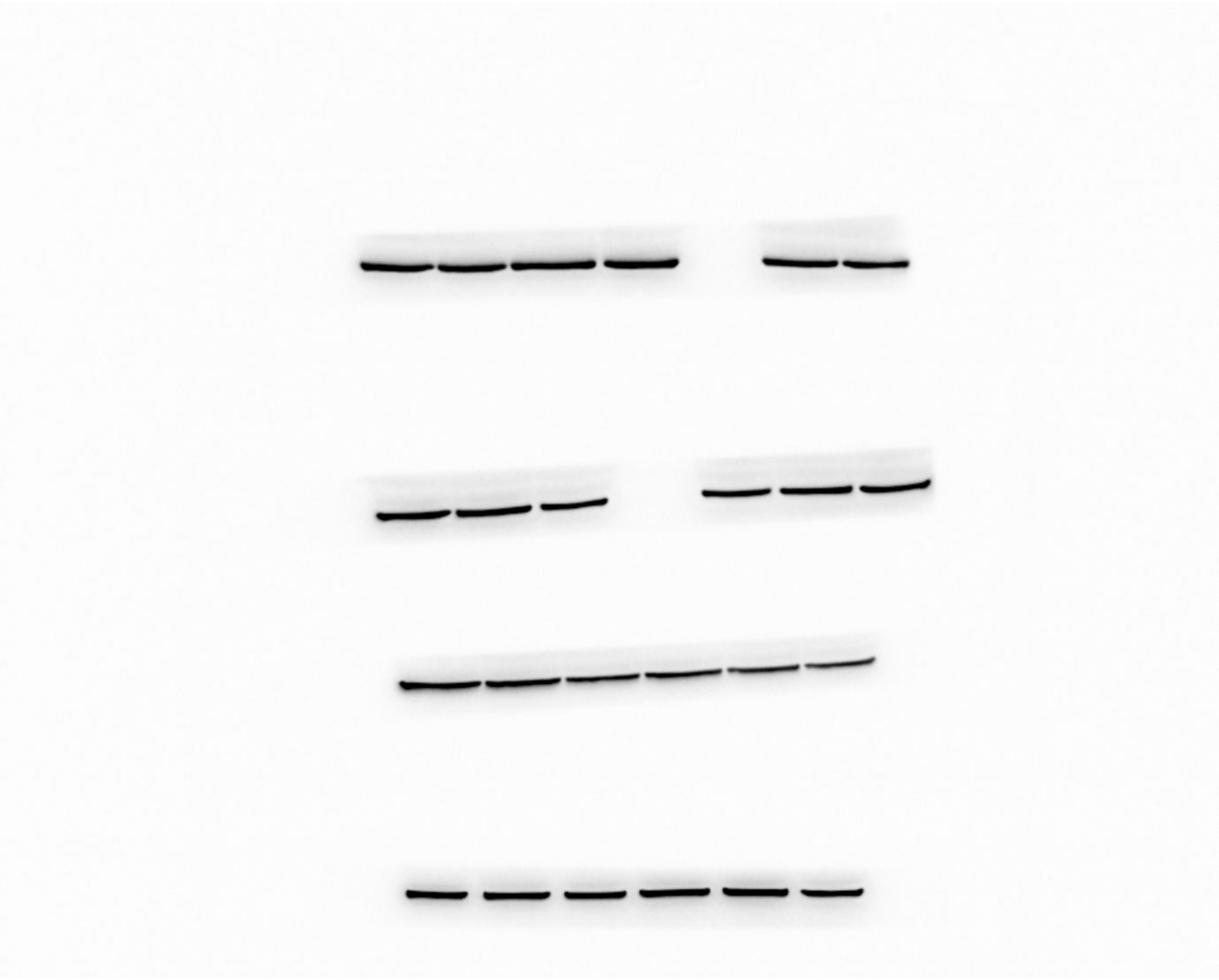

Figure 5 I

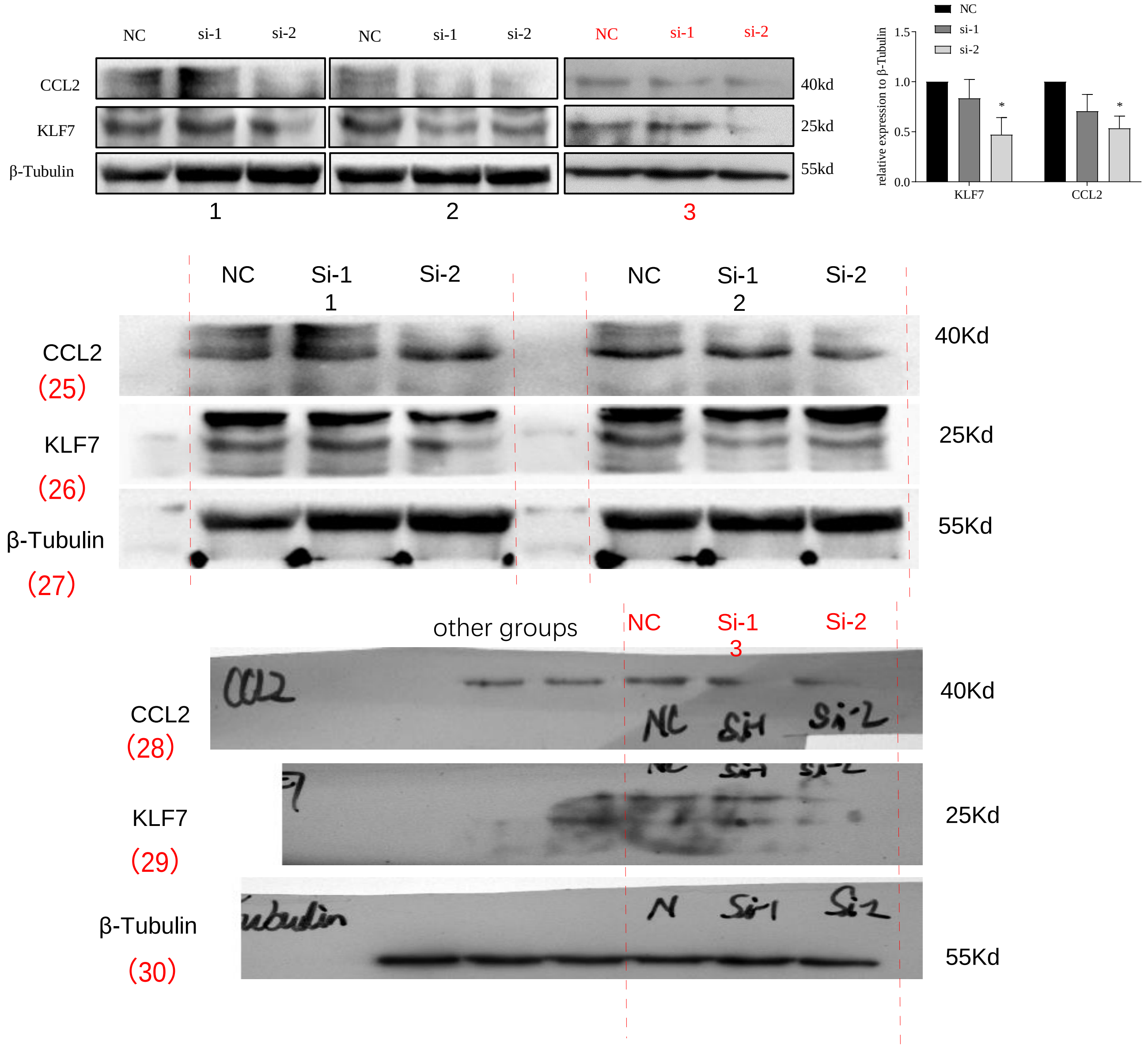

NC
si-1
si-2
NC
si-1
si-2
NC
si-1
si-2
CCL2
KLF7
β-Tubulin
40kd
25kd
55kd
1
2
3
NC
si-1
si-2
relative expression to β-Tubulin
1.5
1.0
0.5
0.0
KLF7
CCL2
NC
Si-1
Si-2
1
NC
Si-1
Si-2
2
CCL2
(25)
KLF7
(26)
β-Tubulin
(27)
40Kd
25Kd
55Kd
other groups
NC
Si-1
Si-2
3
CCL2
(28)
KLF7
(29)
β-Tubulin
(30)
40Kd
25Kd
55Kd

Original membrane for (25)

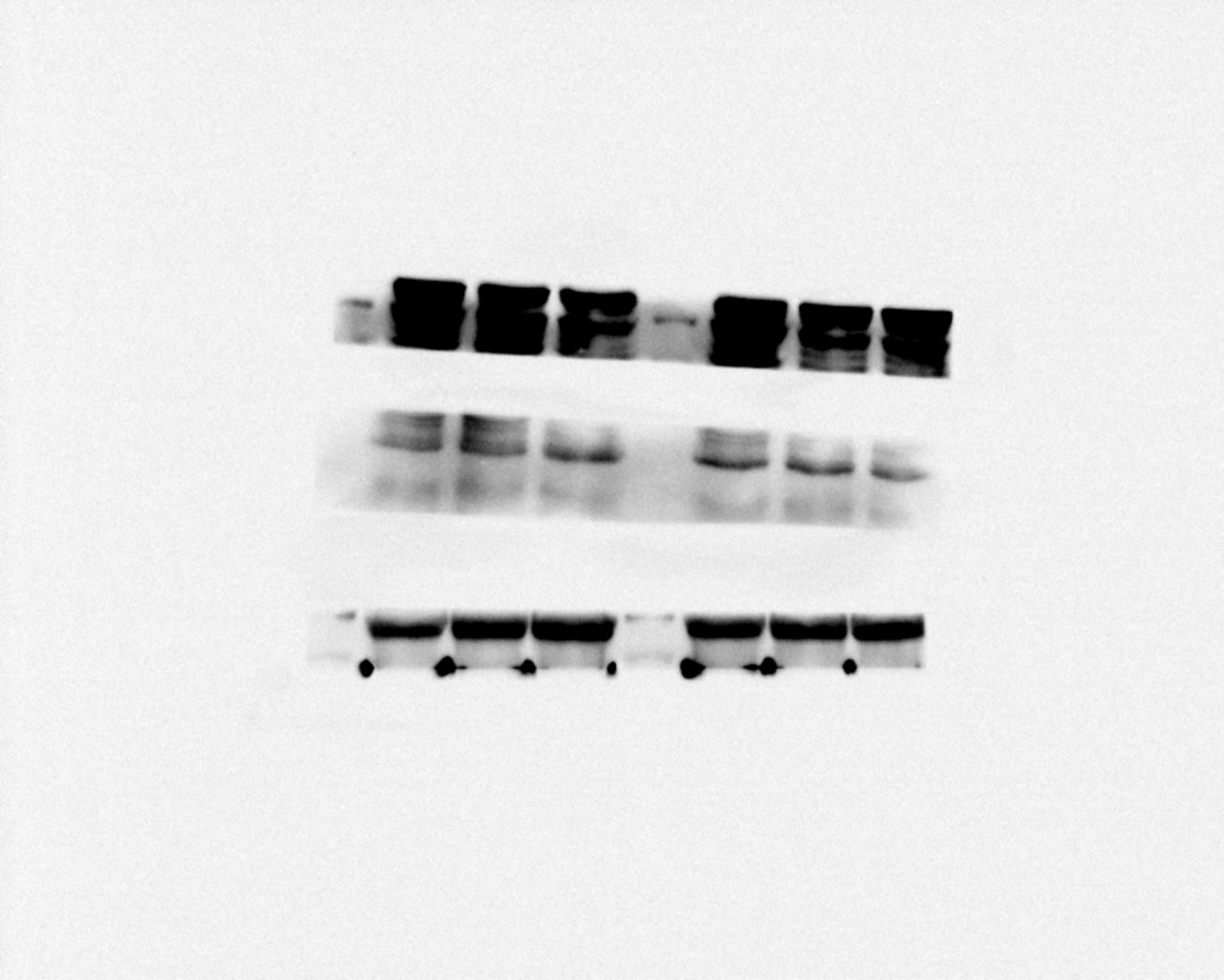

Original membrane for (26)

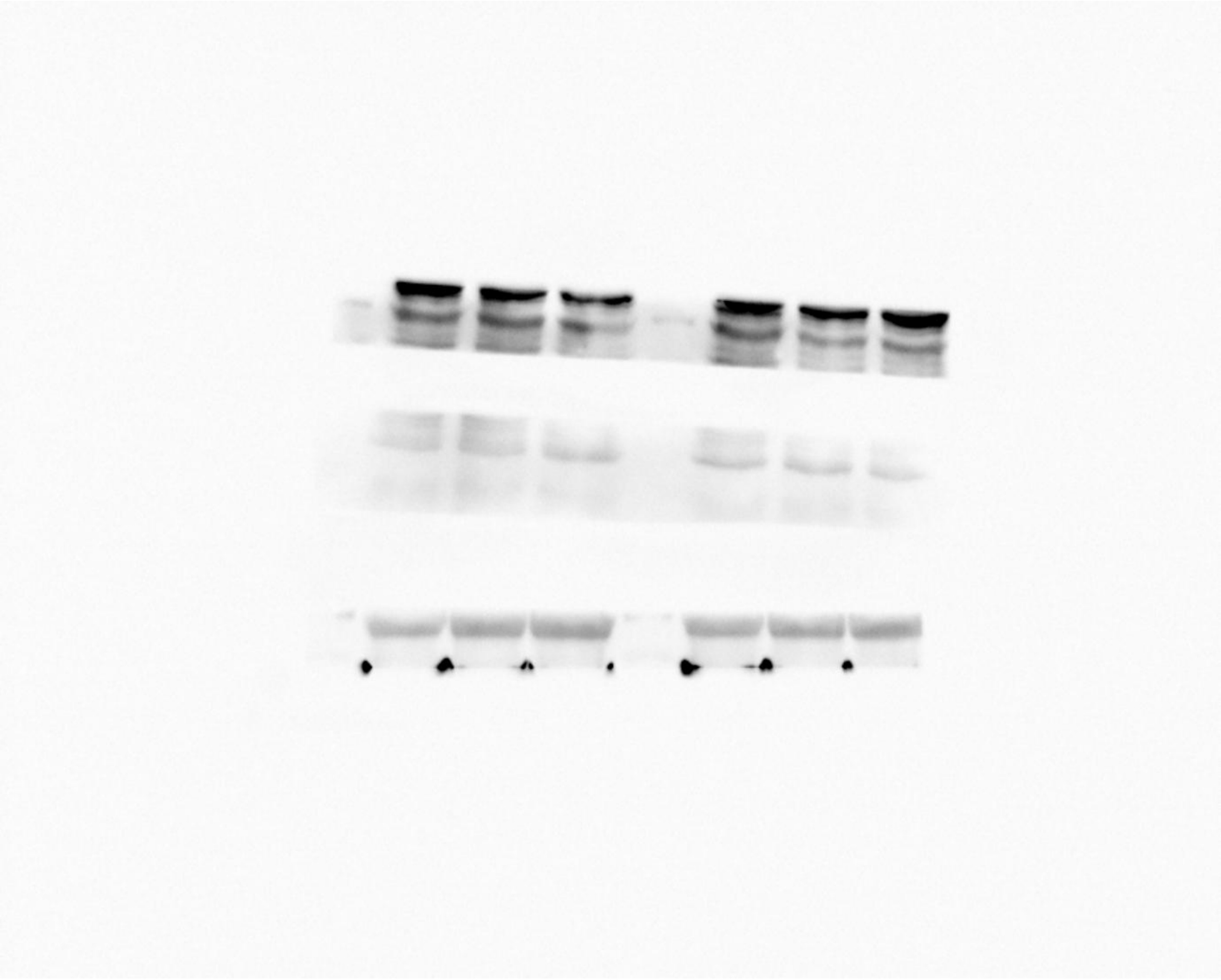

Original membrane for (27)

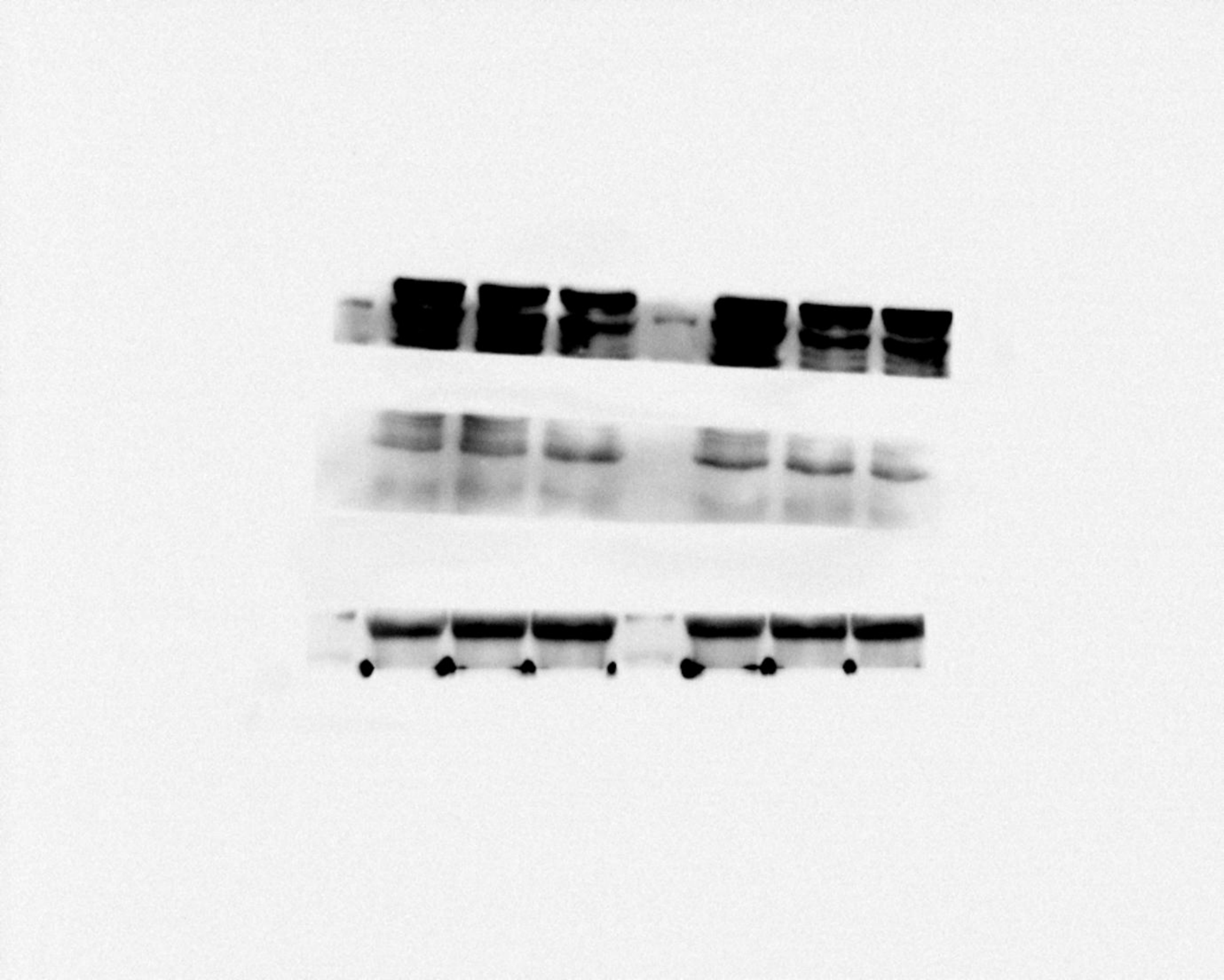

Original membrane for (28)

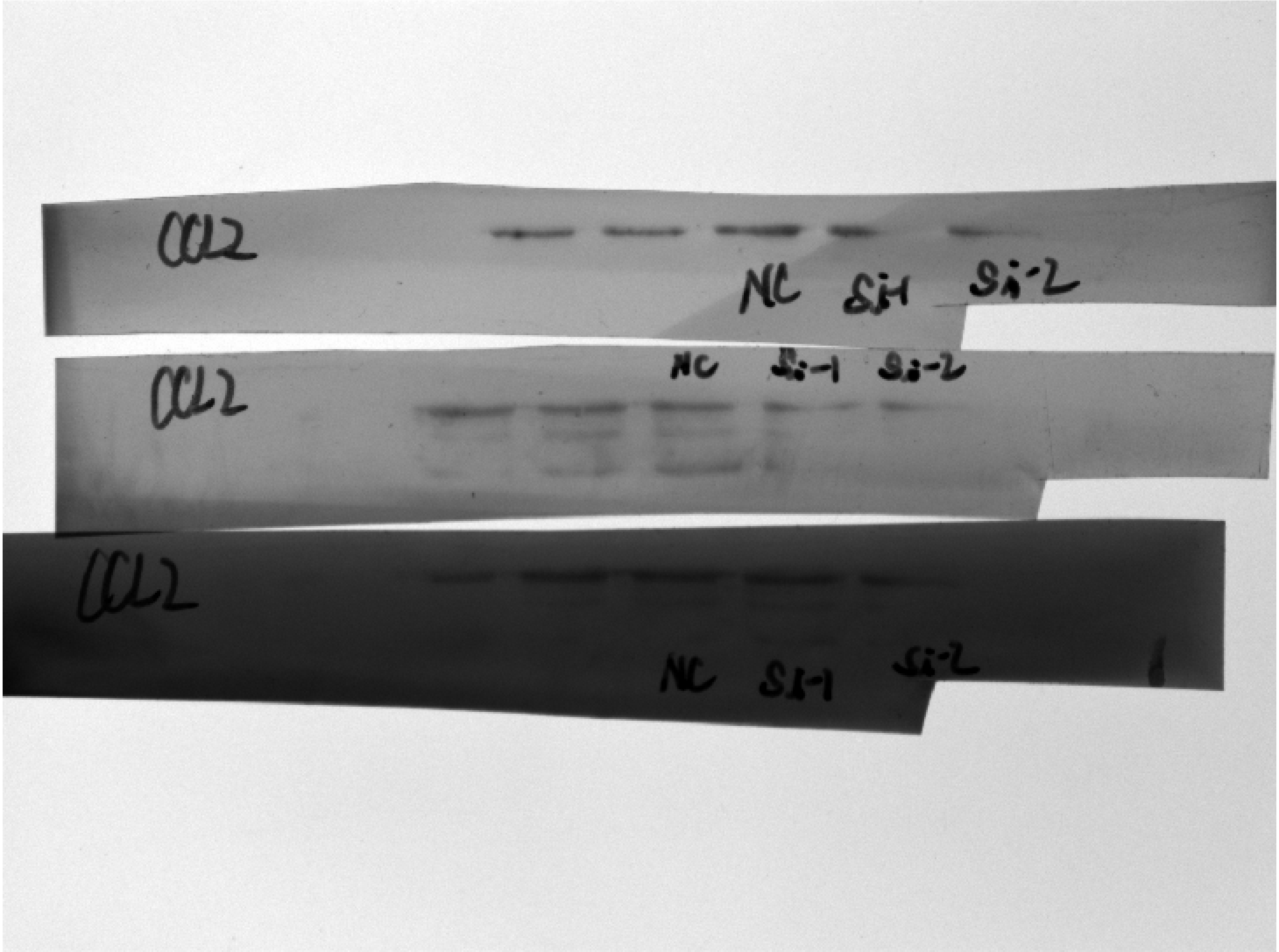

Original membrane for (29)

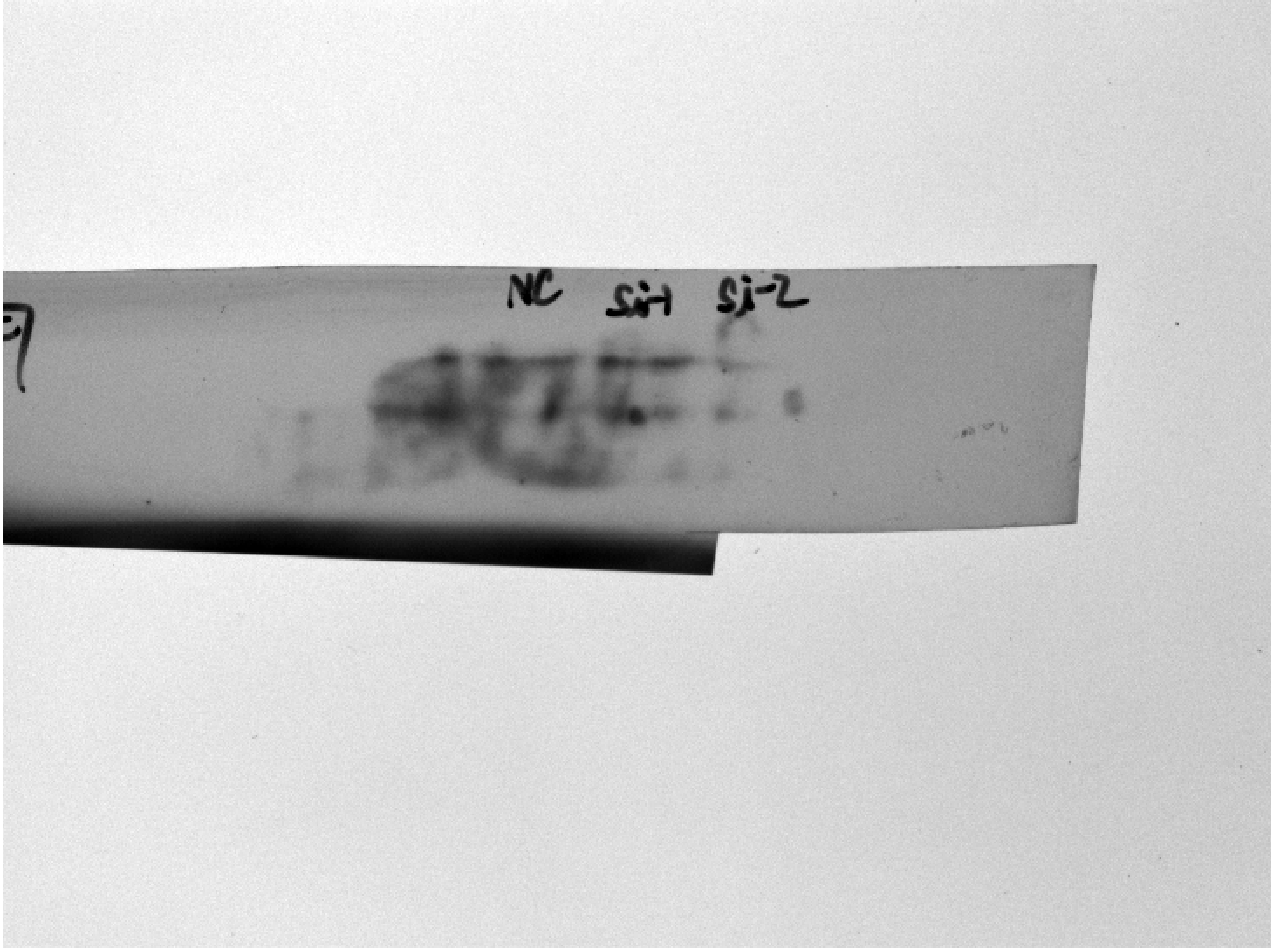

Original membrane for (30)

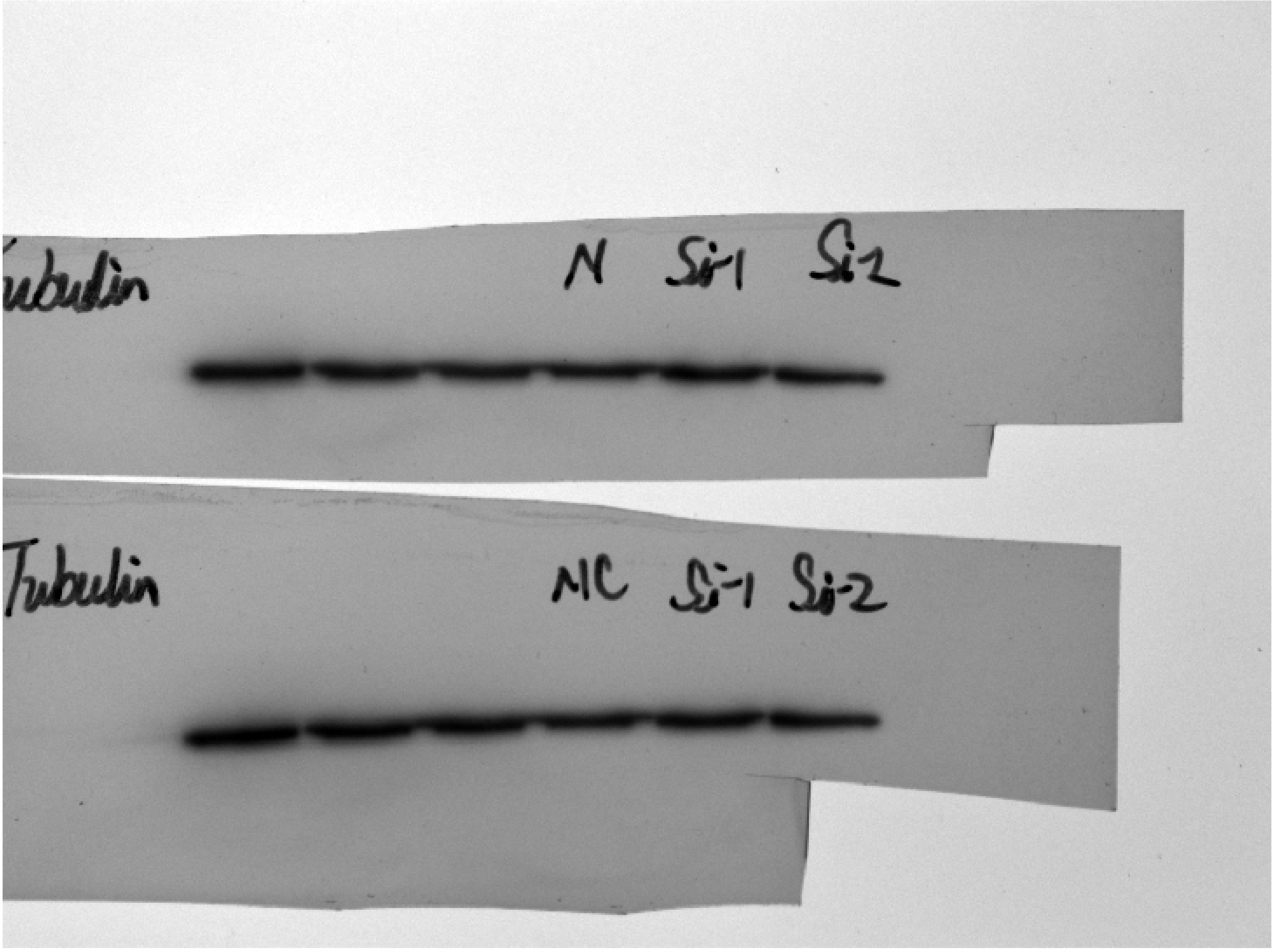

Figure 5 O

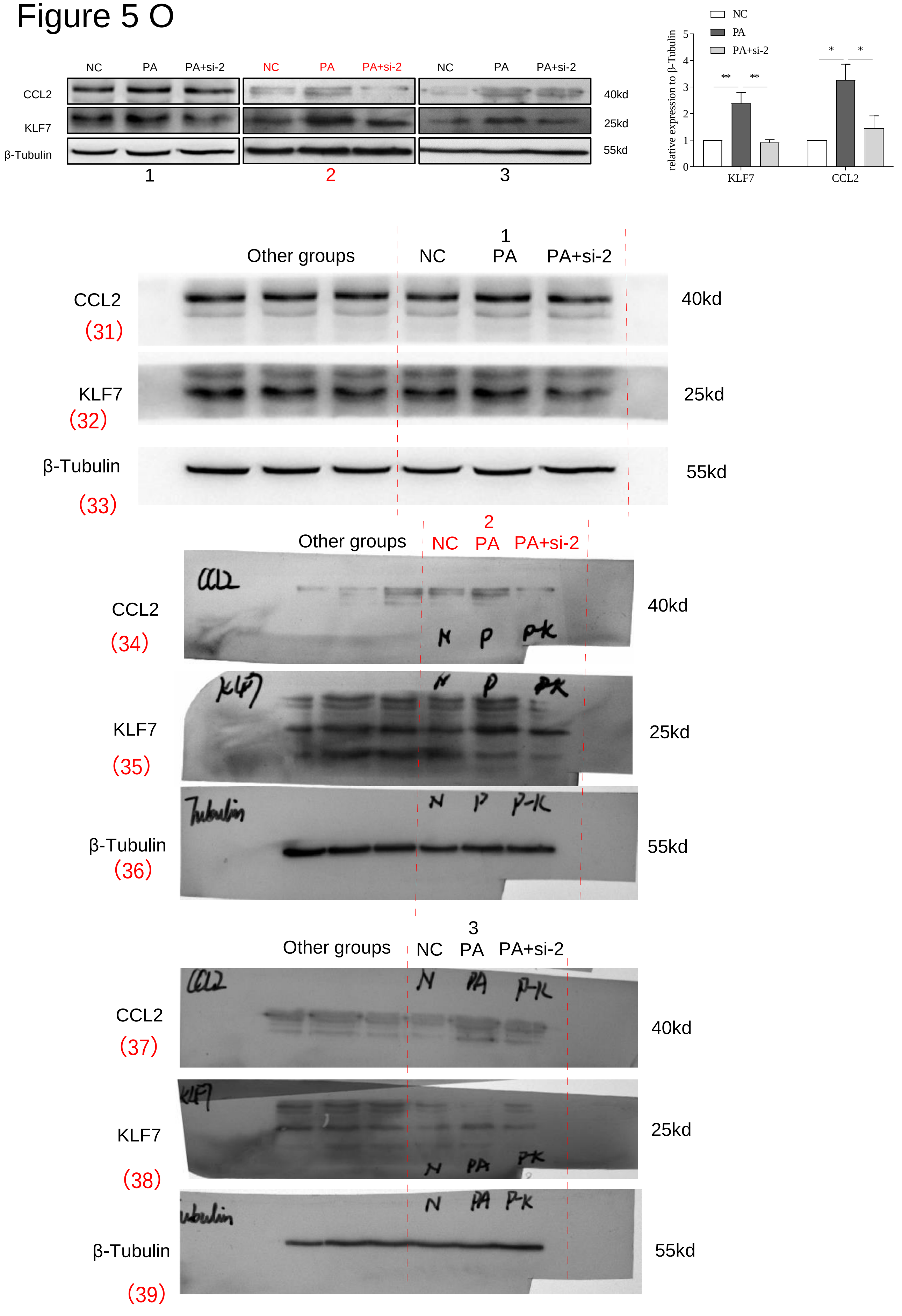

NC PA PA+si-2 NC PA PA+si-2 NC PA PA+si-2
CCL2
KLF7
β-Tubulin
40kd
25kd
55kd
1 2 3
relative expression to β-Tubulin
NC
PA
PA+si-2
KLF7
CCL2
Other groups
1
NC PA PA+si-2
CCL2 (31)
KLF7 (32)
β-Tubulin (33)
2
CCL2 (34)
KLF7 (35)
β-Tubulin (36)
3
CCL2 (37)
KLF7 (38)
β-Tubulin (39)

Original membrane for (31)

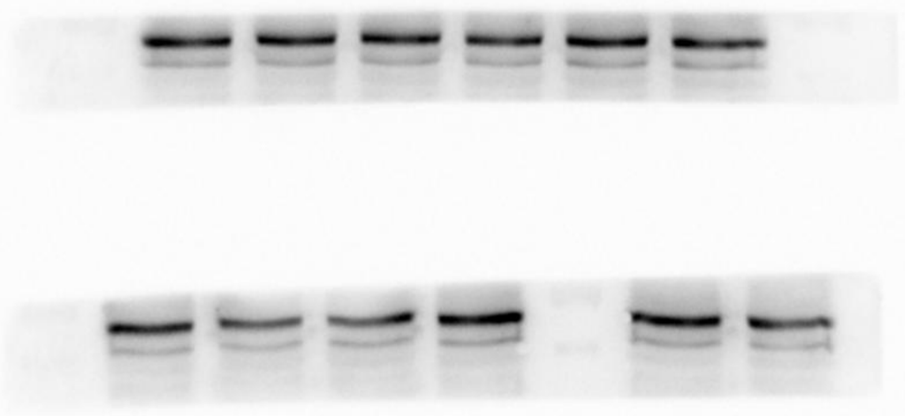

Original membrane for (32)

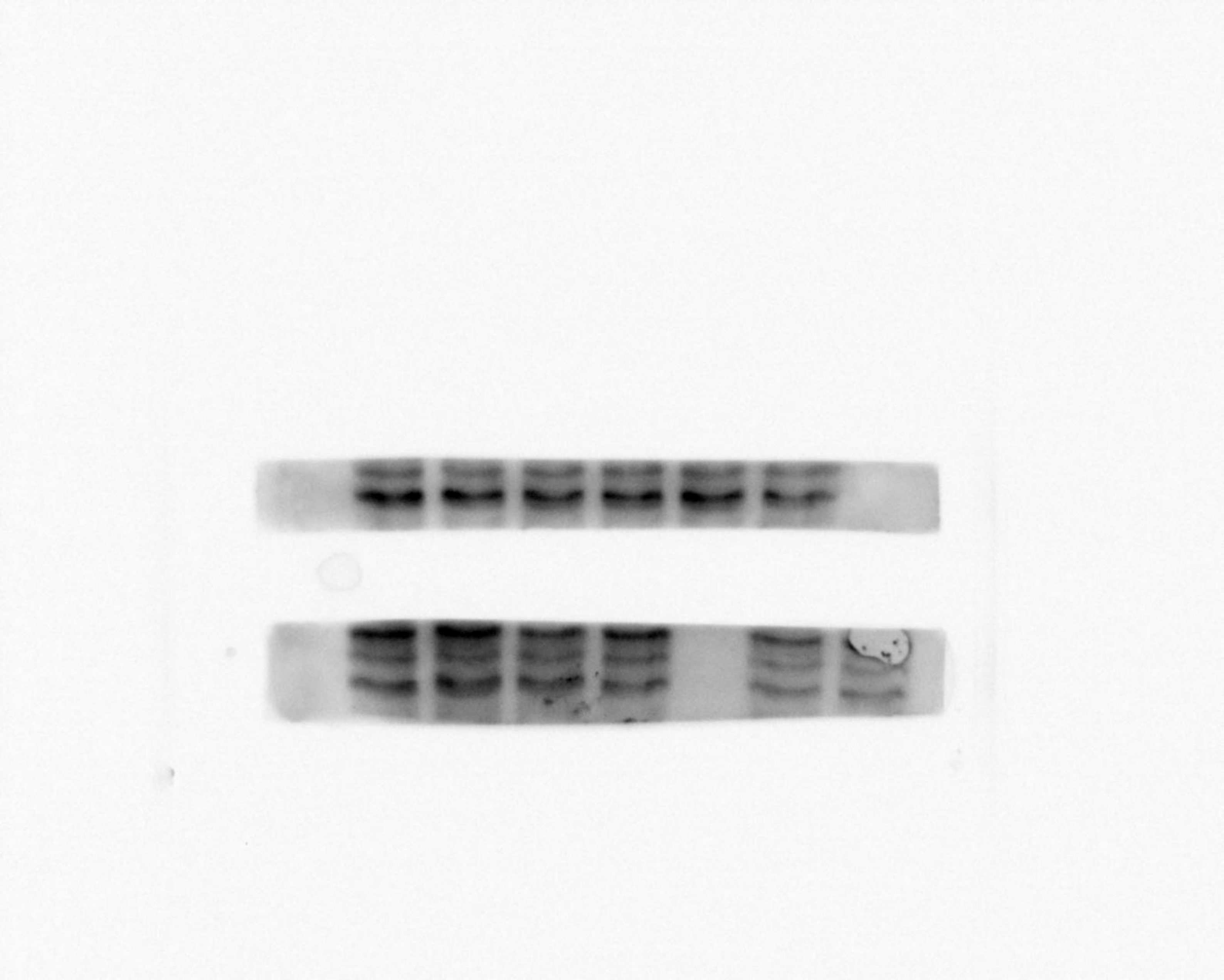

Original membrane for (33)

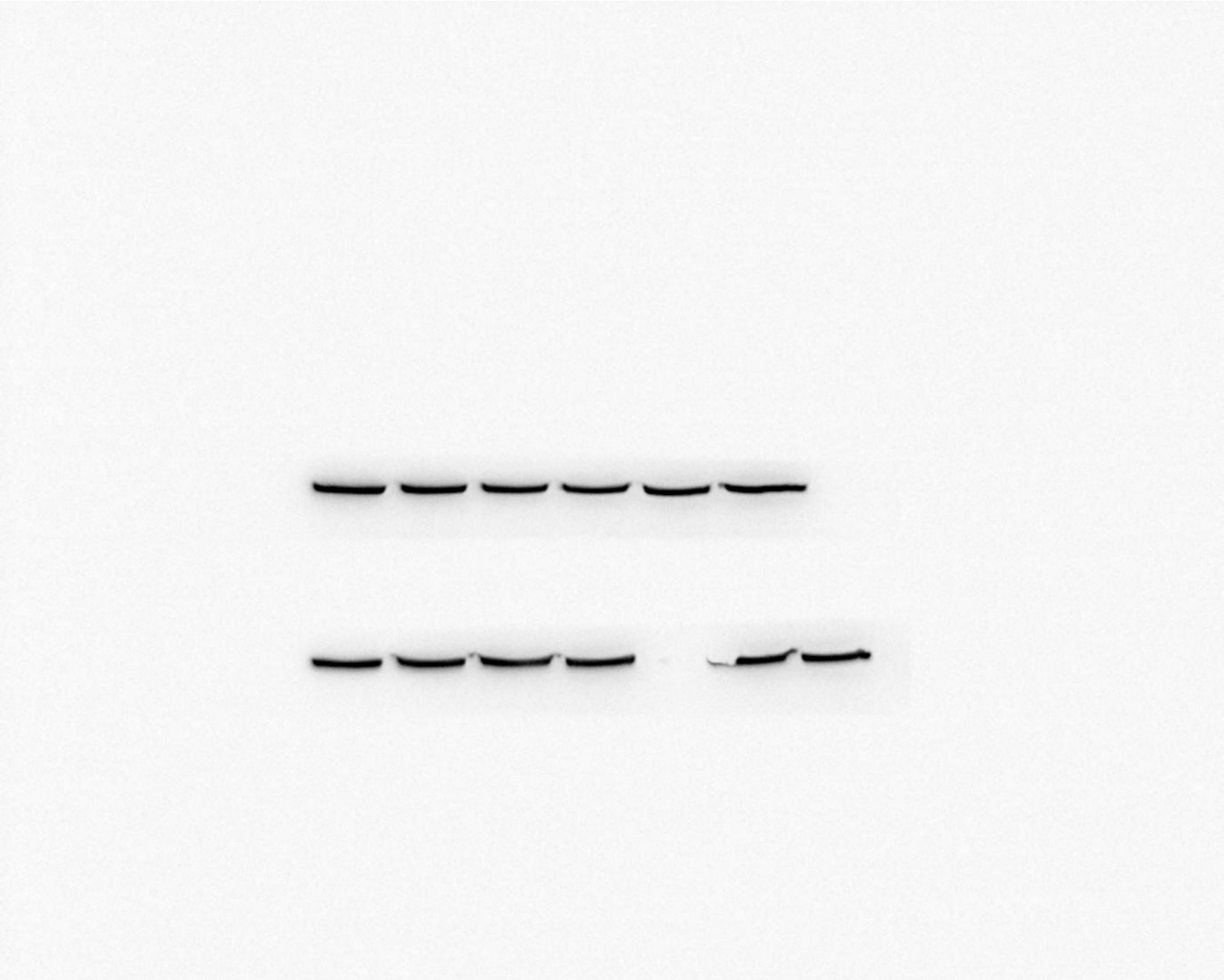

Original membrane for (34)

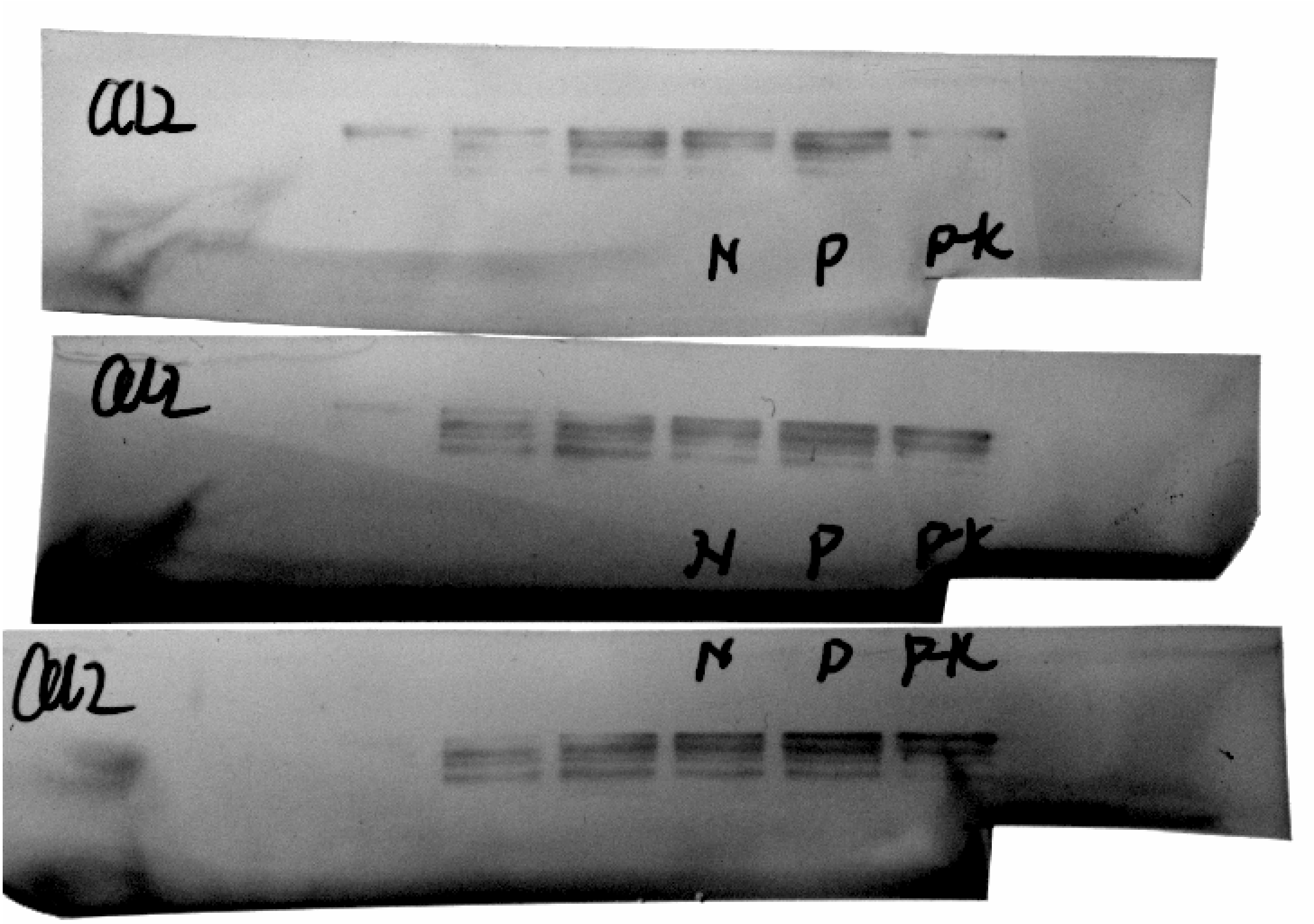

Original membrane for (35)

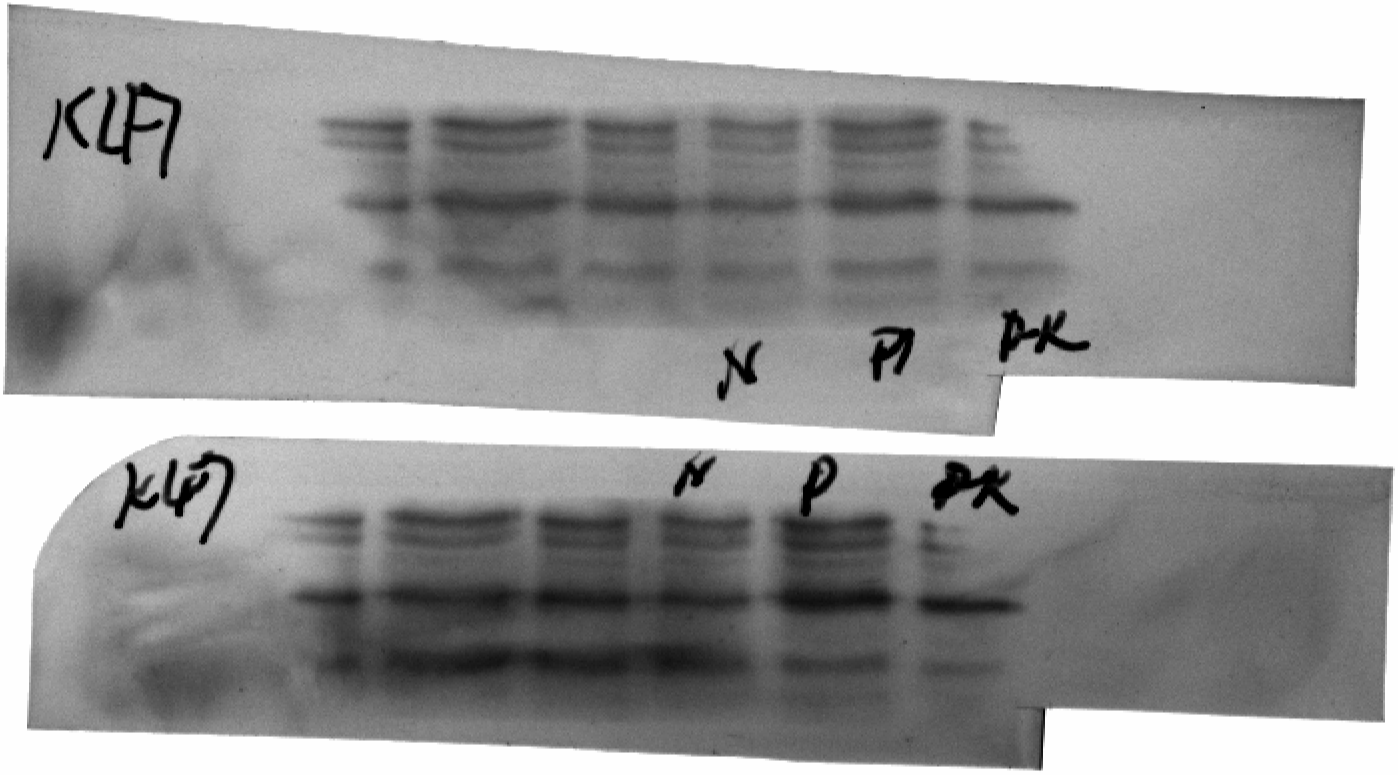

Original membrane for (36)

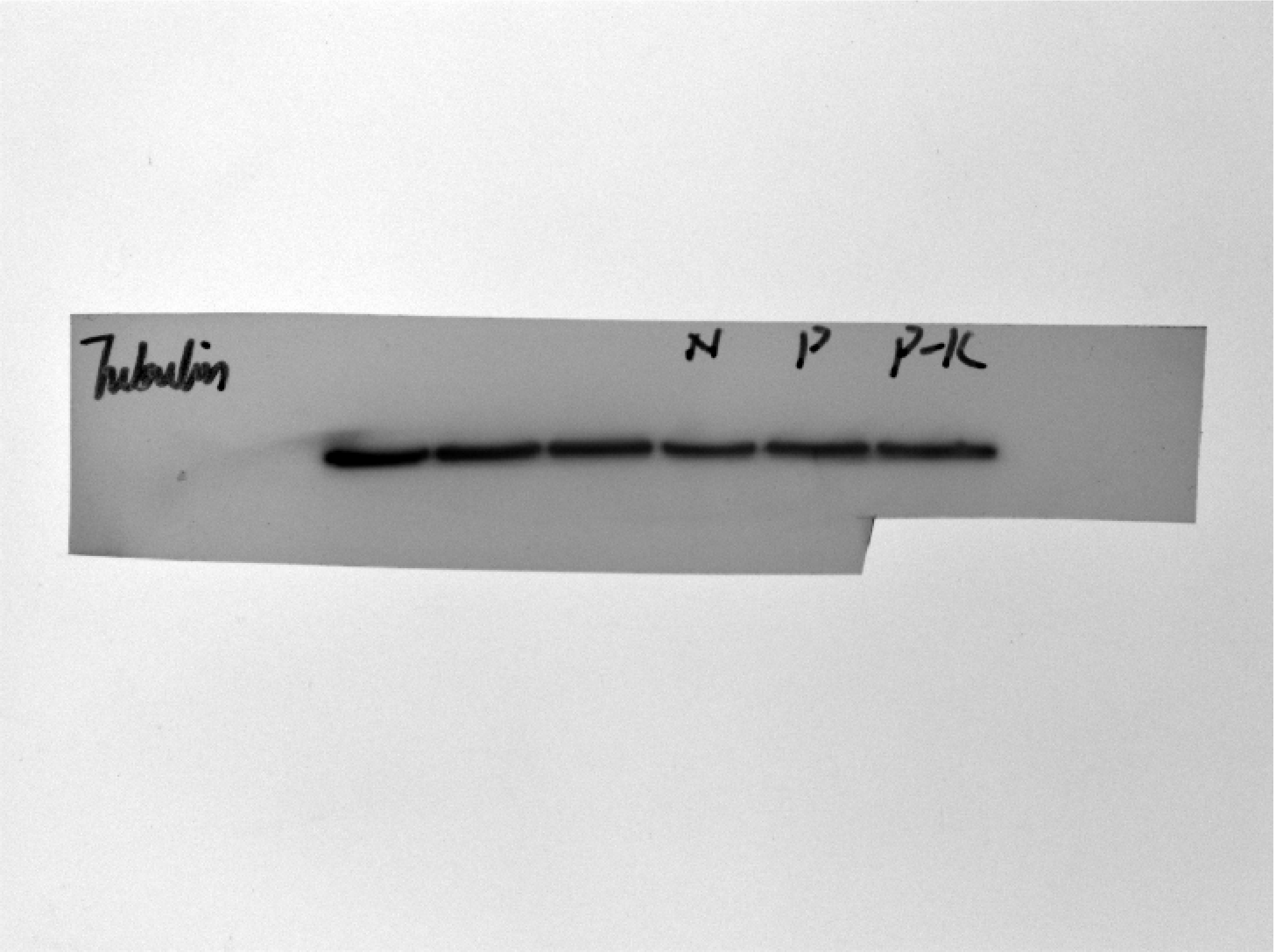

Original membrane for (37)

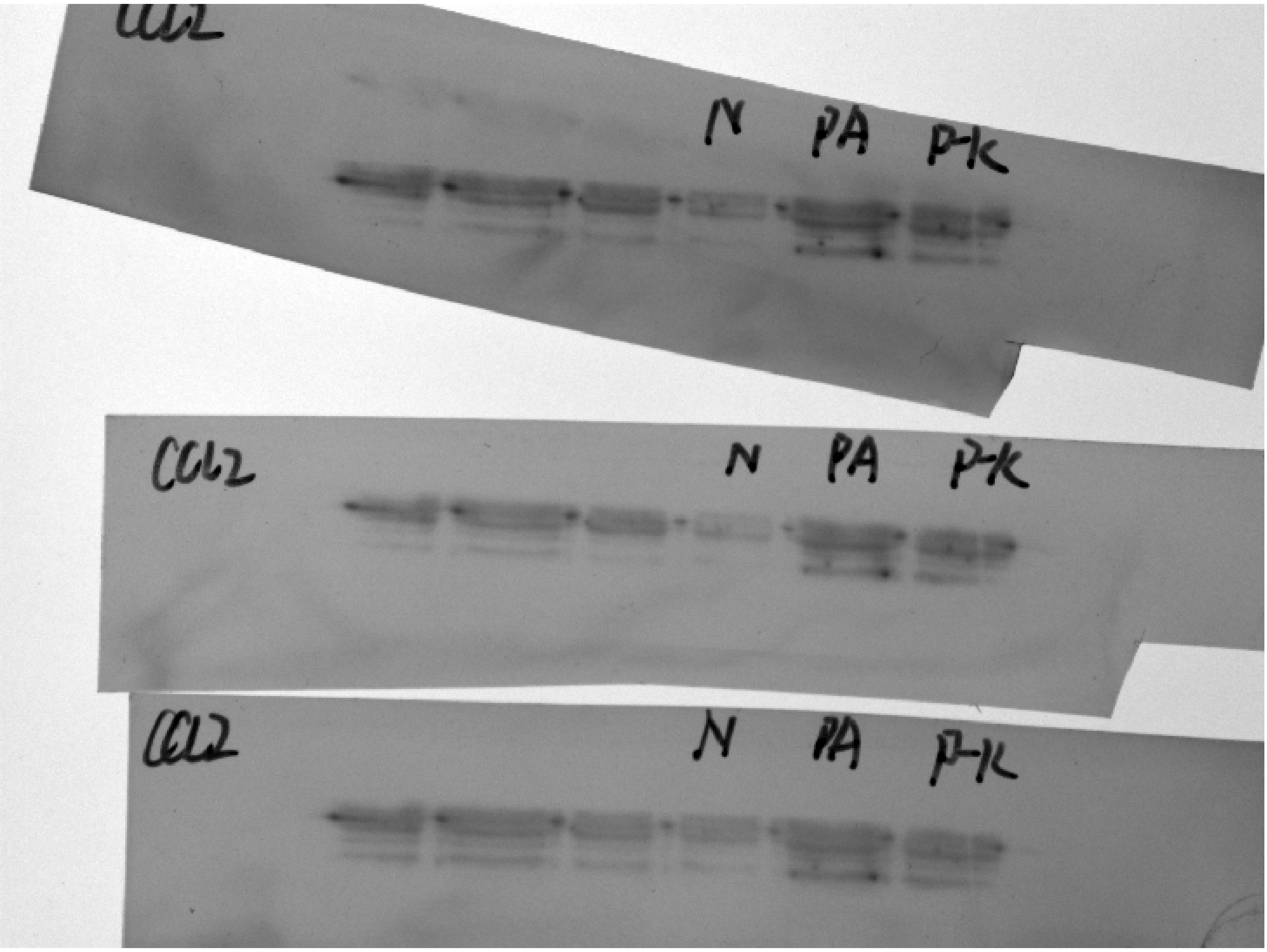

Original membrane for (38)

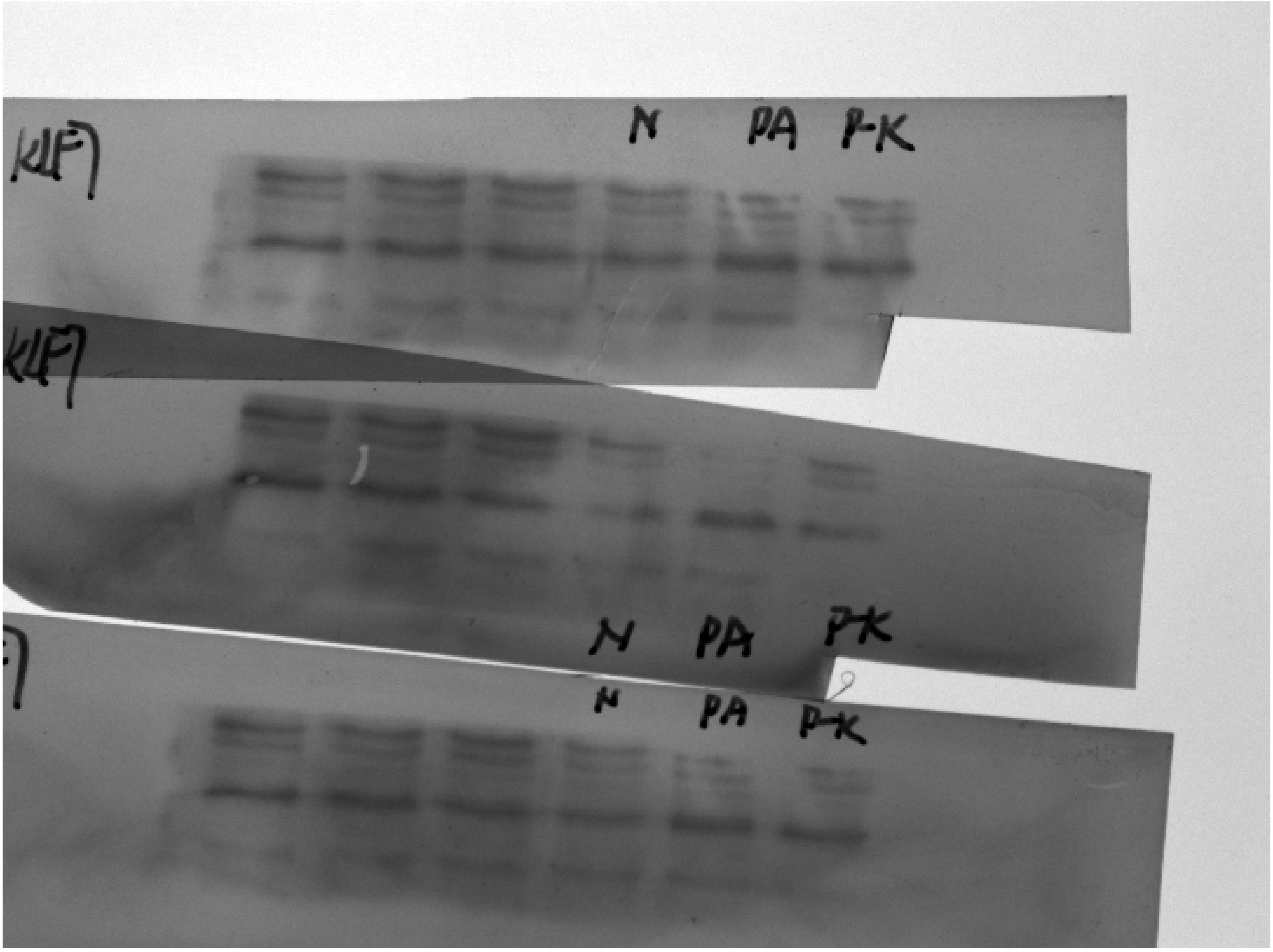

Original membrane for (39)

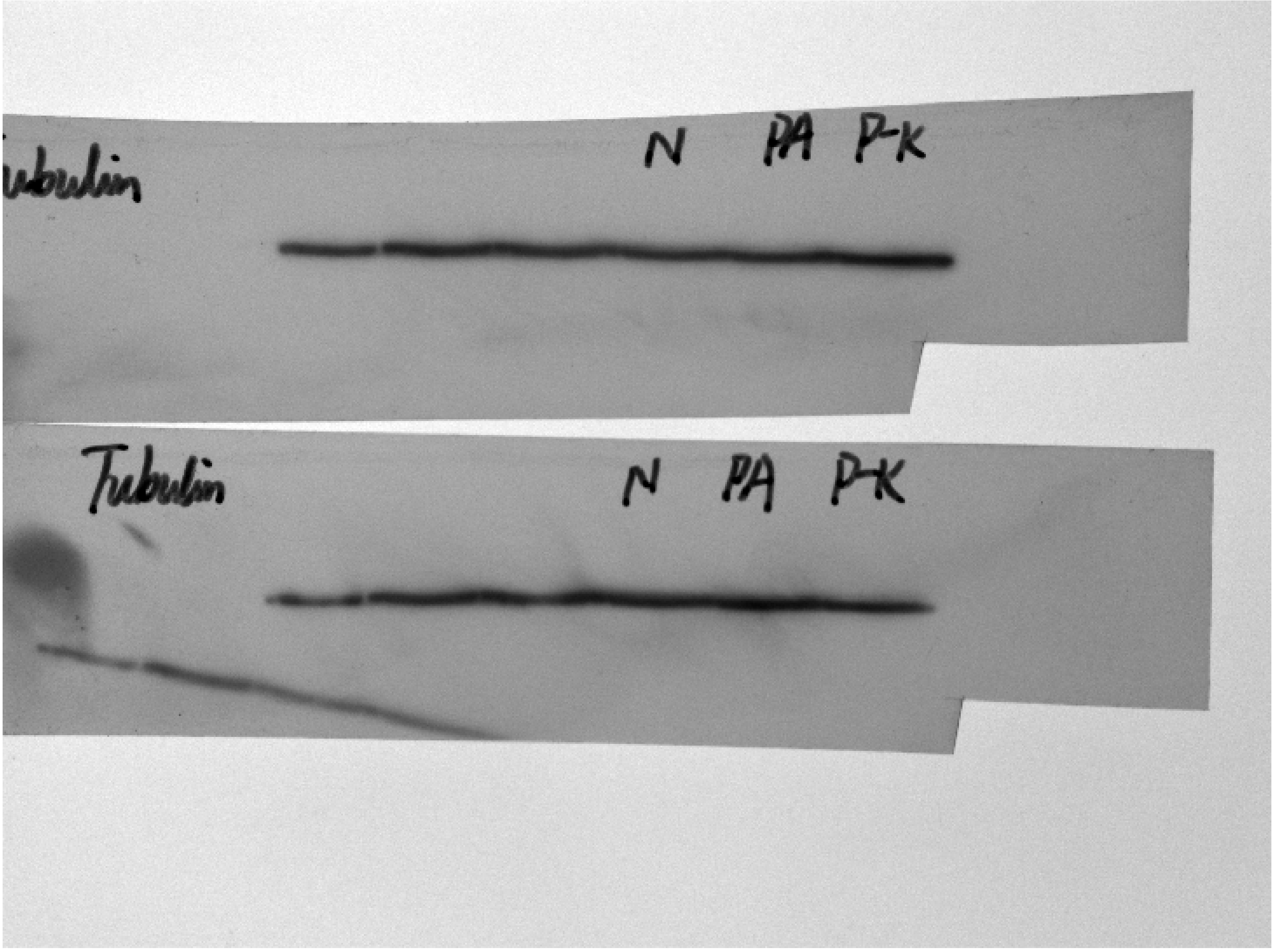

Figure 6 D、E

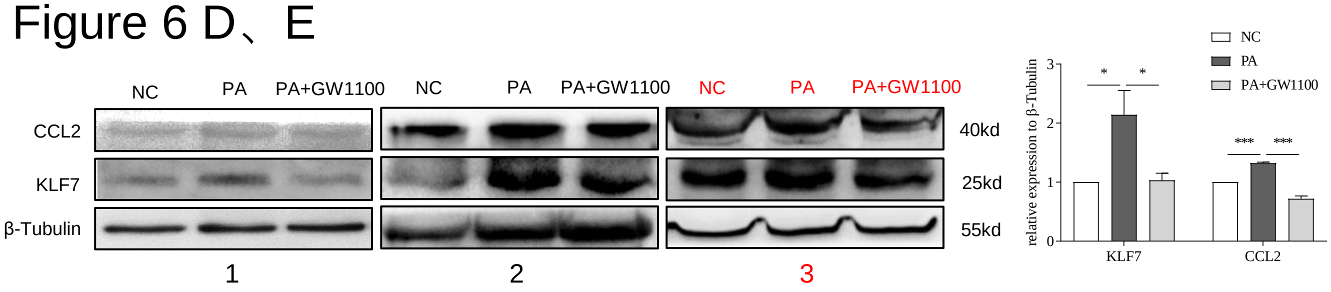
NC
PA
PA+GW1100
NC
PA
PA+GW1100
NC
PA
PA+GW1100
CCL2
KLF7
β-Tubulin
40kd
25kd
55kd
1
2
3
relative expression to β-Tubulin
NC
PA
PA+GW1100
KLF7
CCL2
*
*
***
***

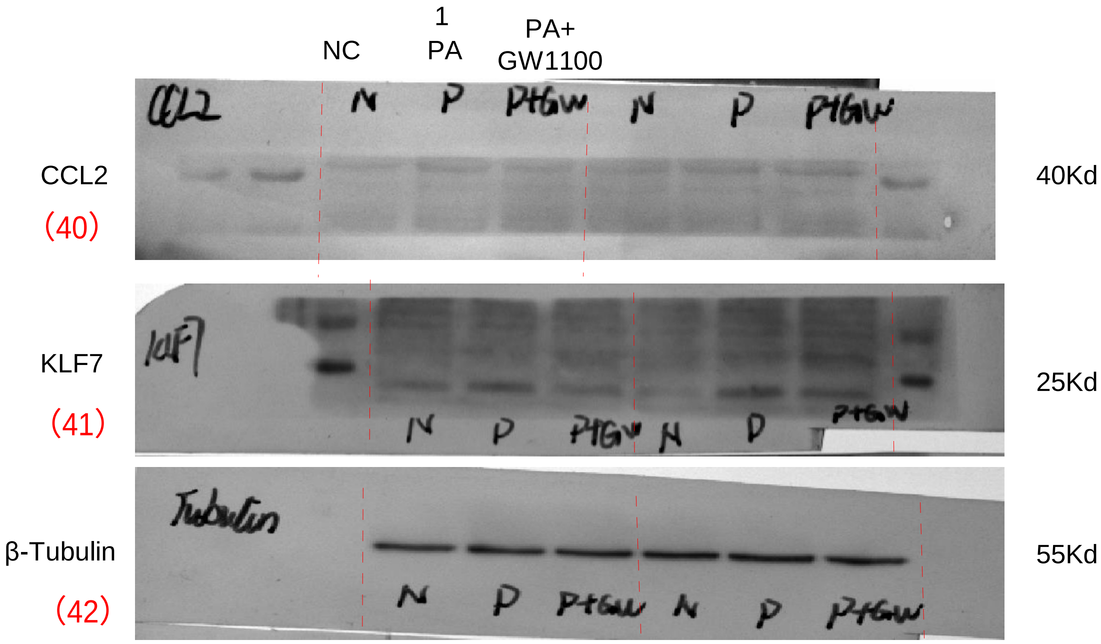
1
NC
PA
PA+
GW1100
CCL2
(40)
KLF7
(41)
β-Tubulin
(42)
40Kd
25Kd
55Kd

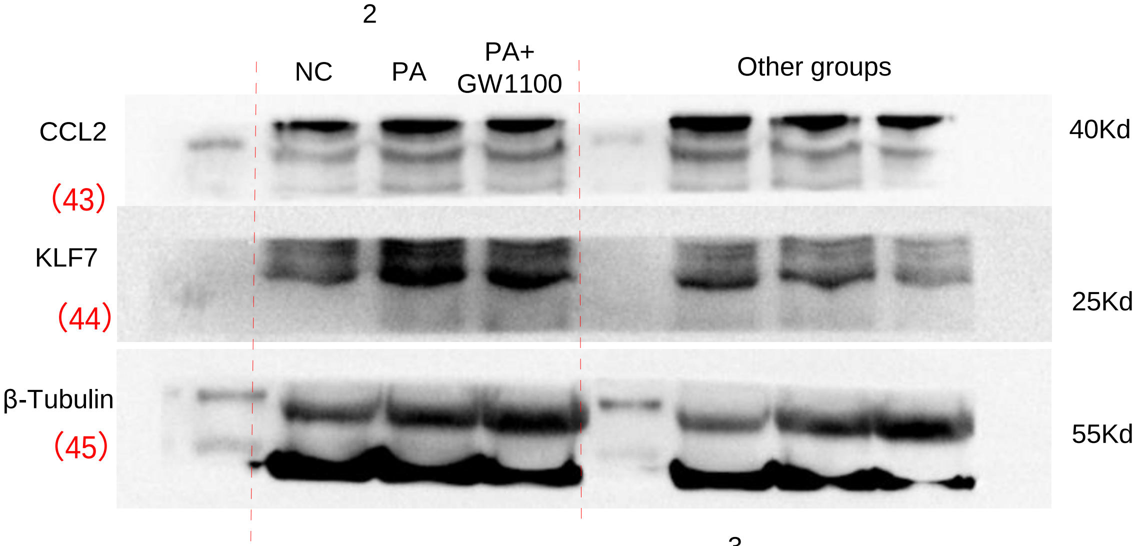
2
NC
PA
PA+
GW1100
Other groups
CCL2
(43)
KLF7
(44)
β-Tubulin
(45)
40Kd
25Kd
55Kd

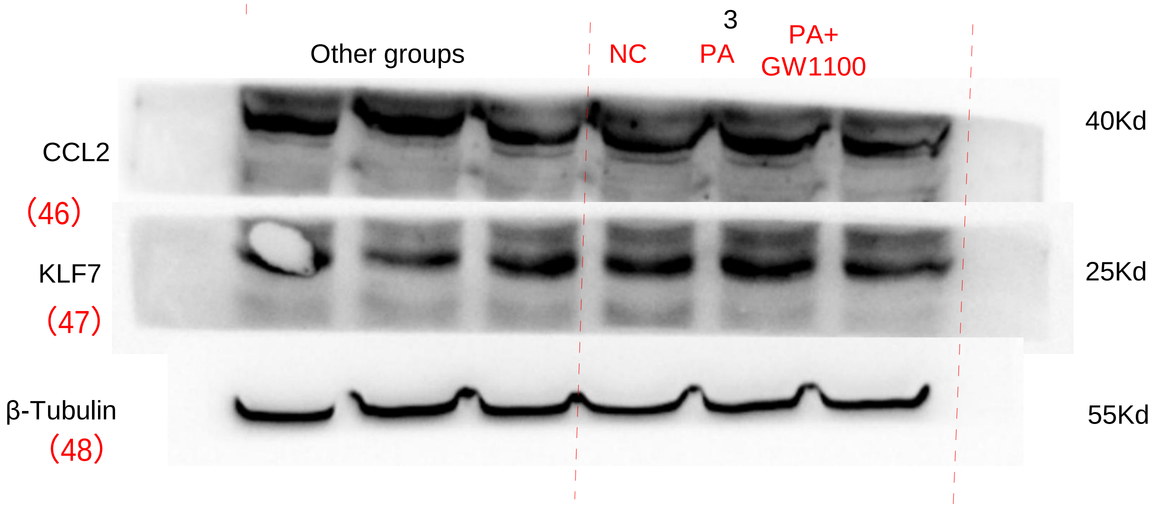
Other groups
3
NC
PA
PA+
GW1100
CCL2
(46)
KLF7
(47)
β-Tubulin
(48)
40Kd
25Kd
55Kd

Original membrane for (40)

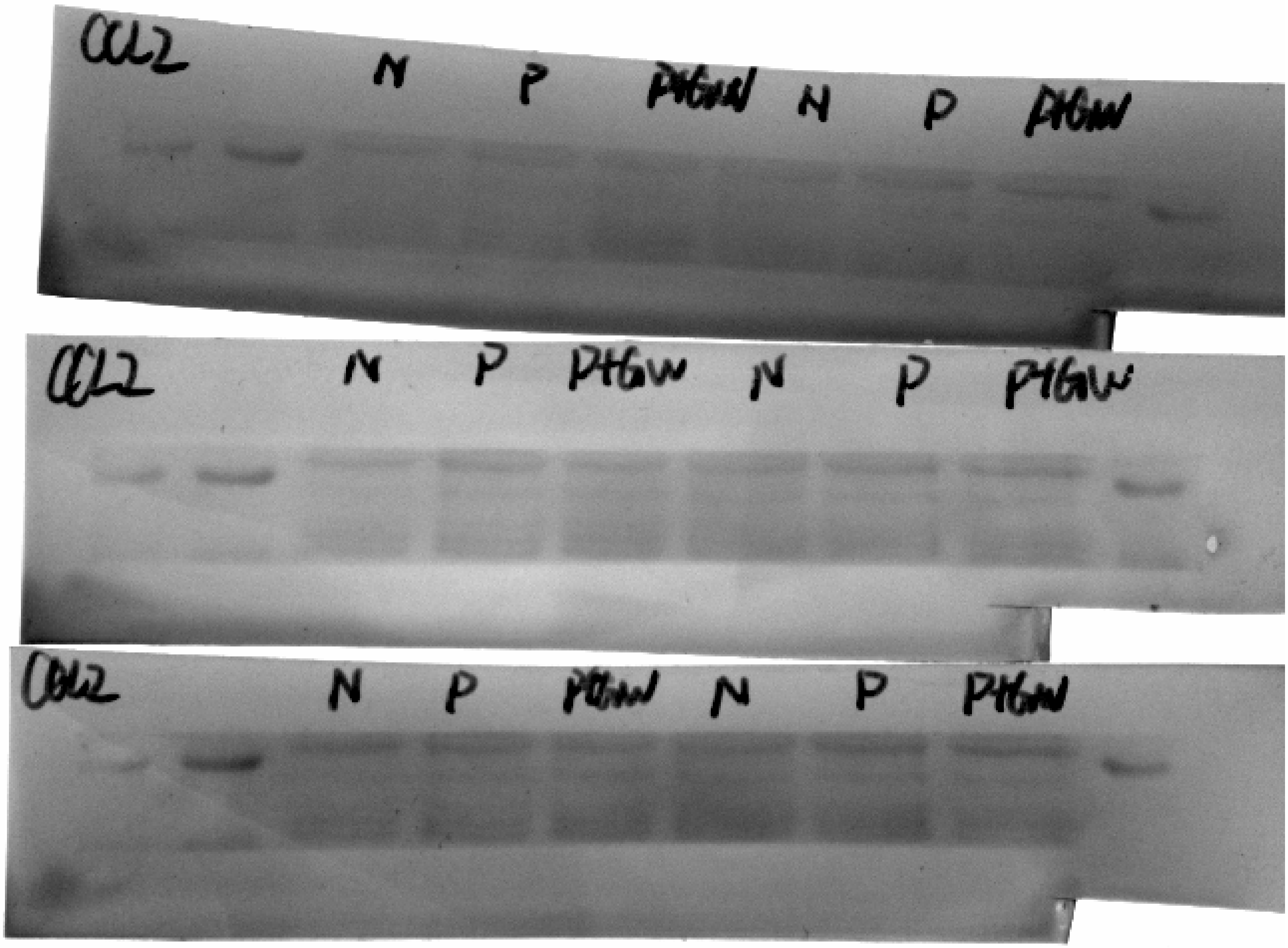

Original membrane for (41)

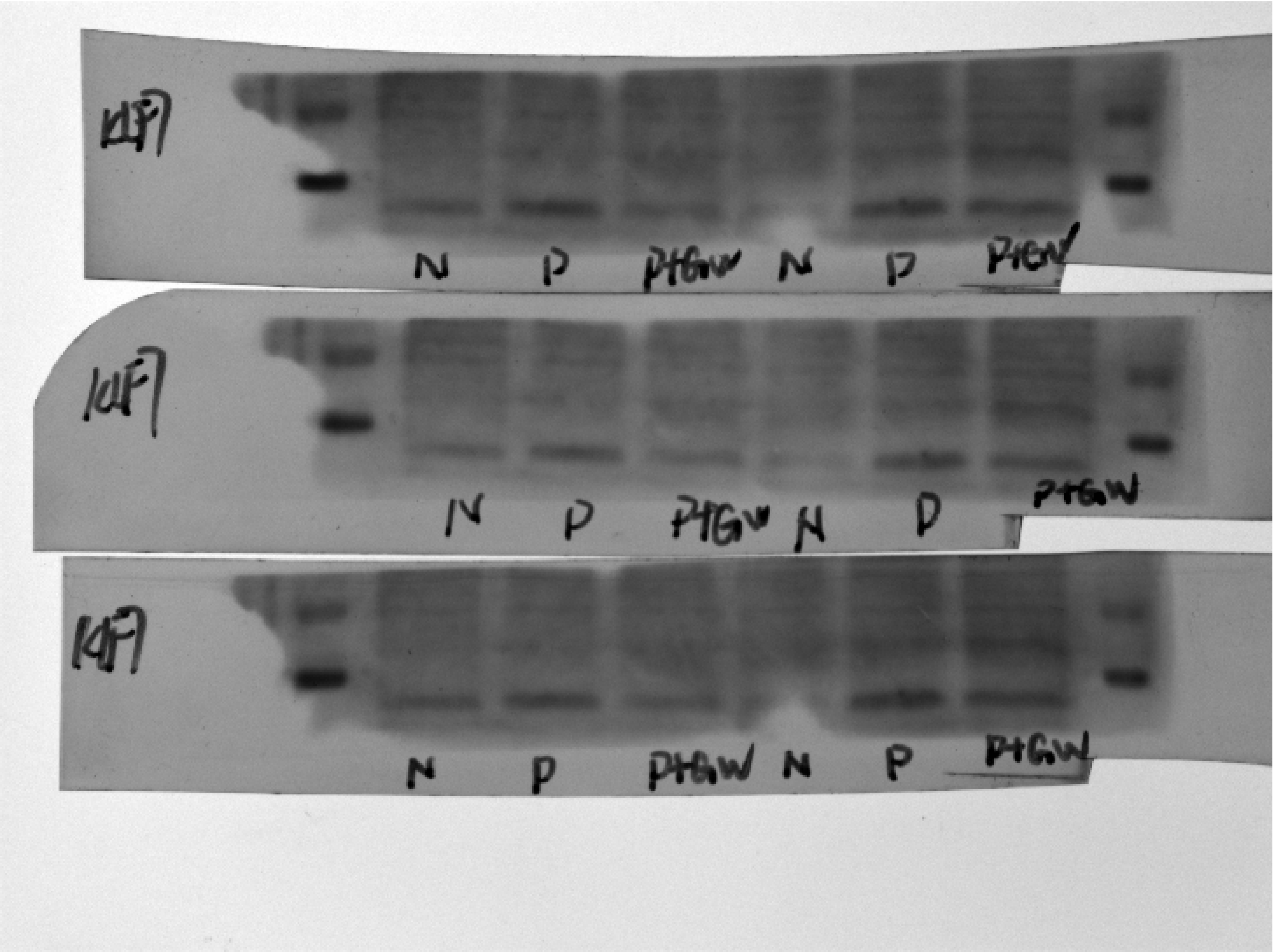

Original membrane for (42)

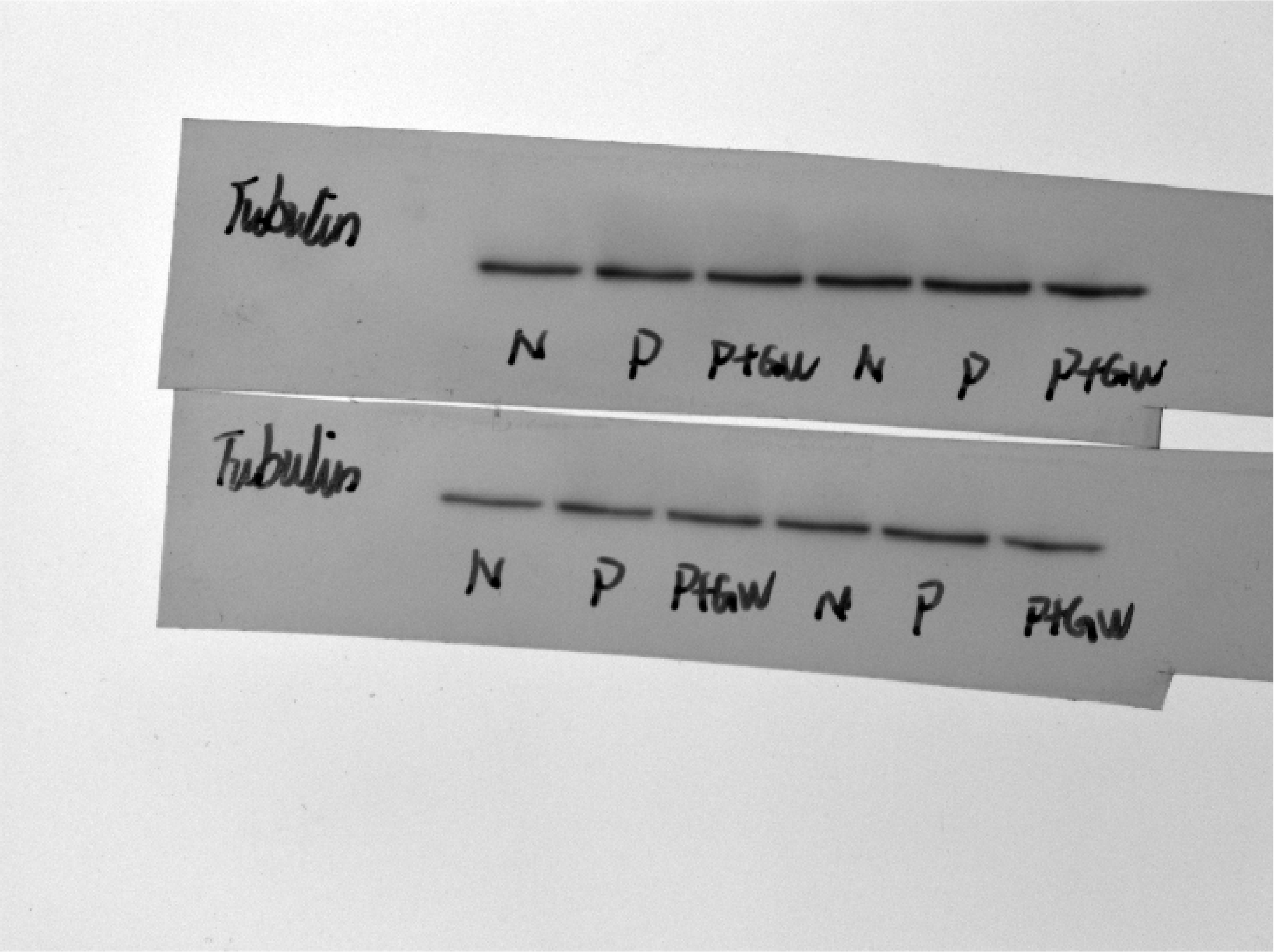

Original membrane for (43)

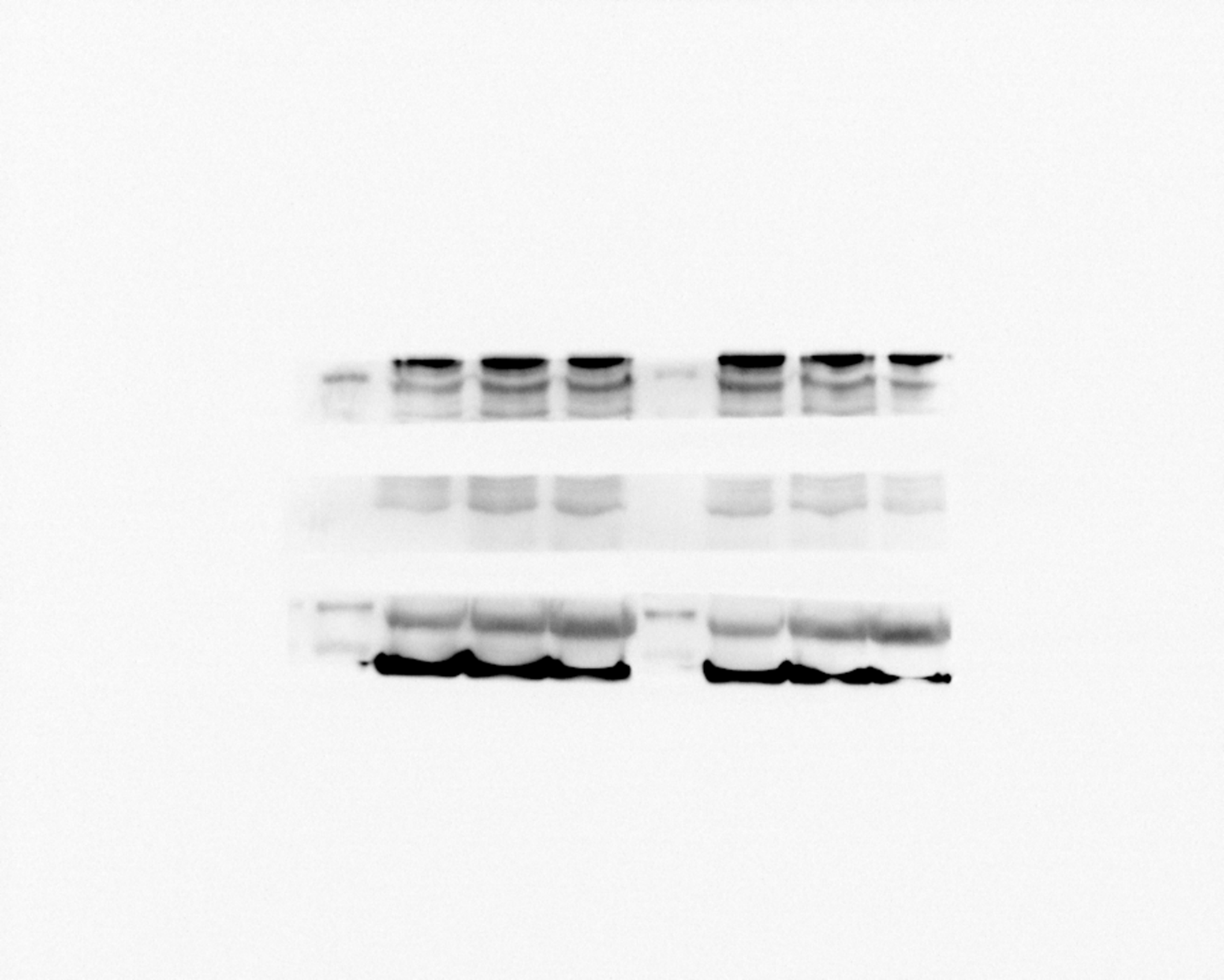

Original membrane for (44)

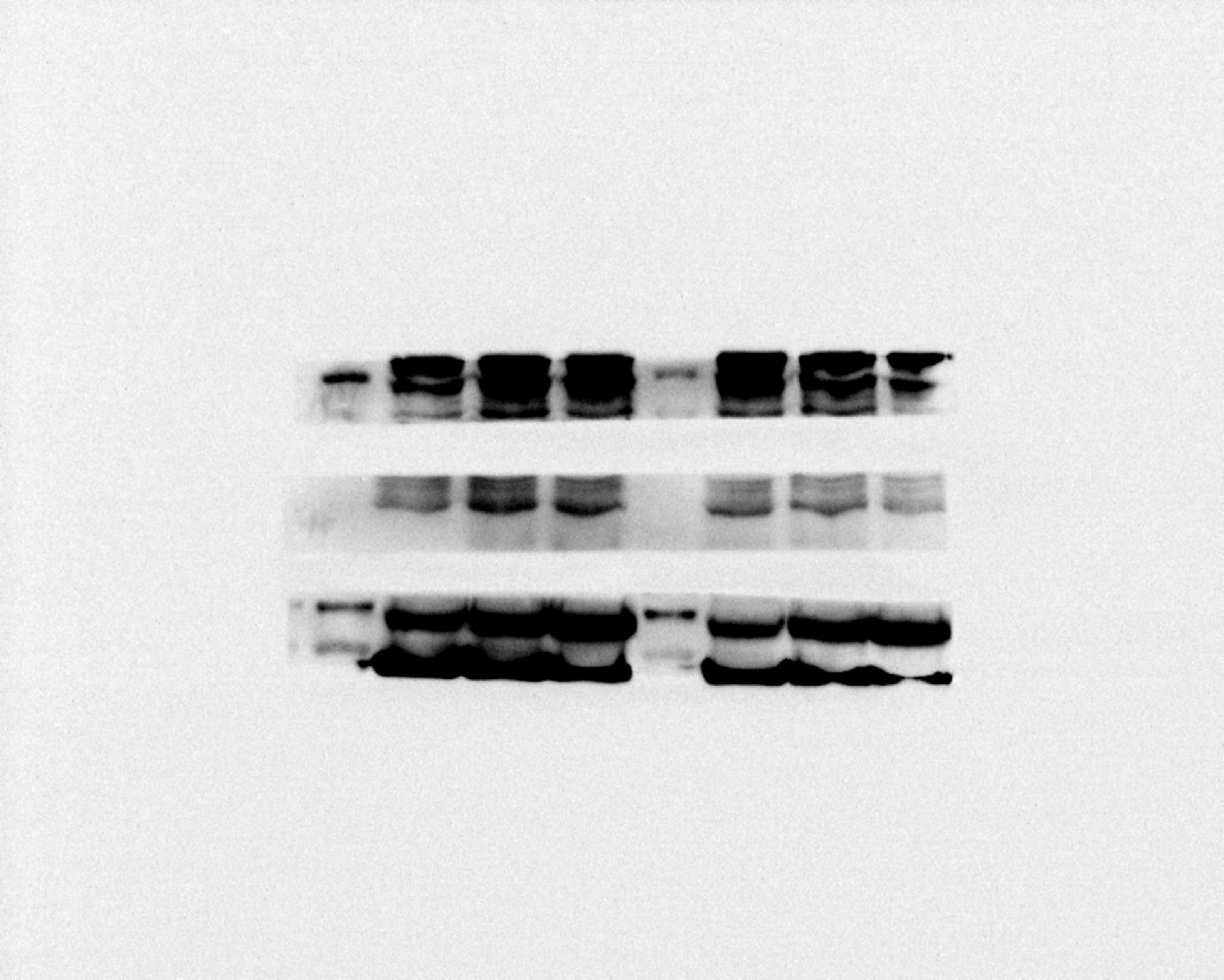

Original membrane for (45)

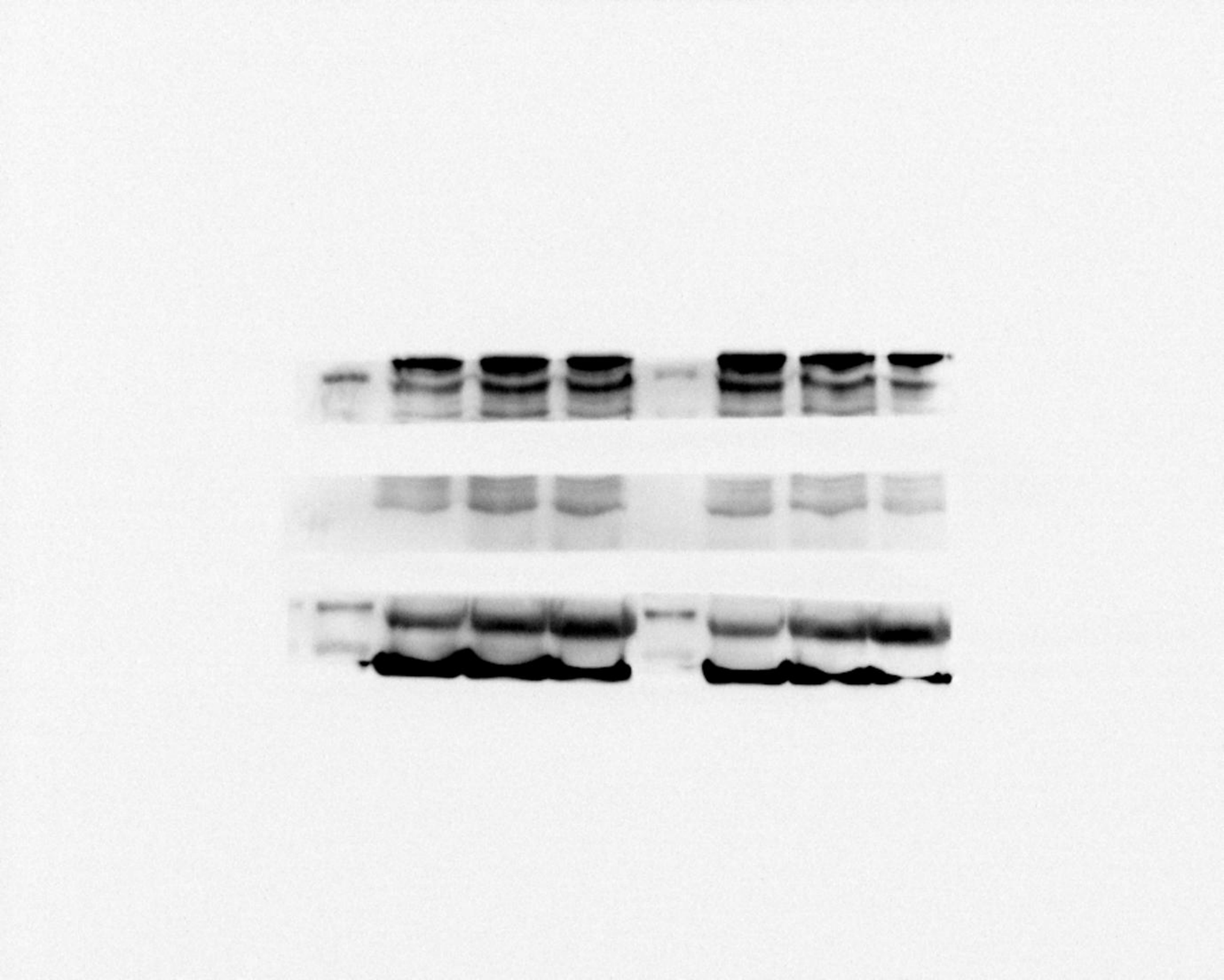

Original membrane for (46)

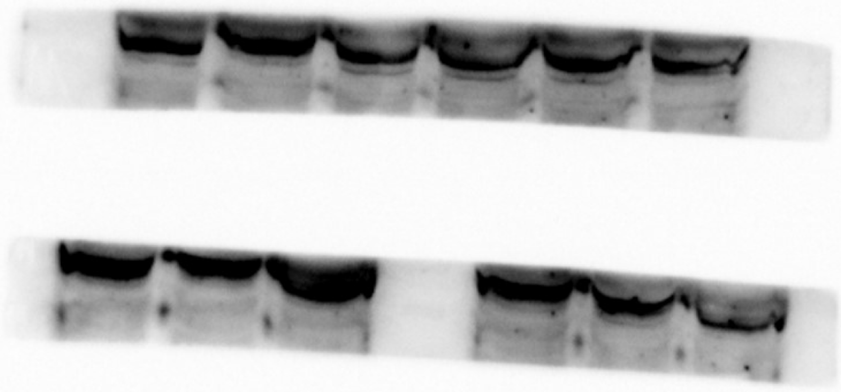

Original membrane for (47)

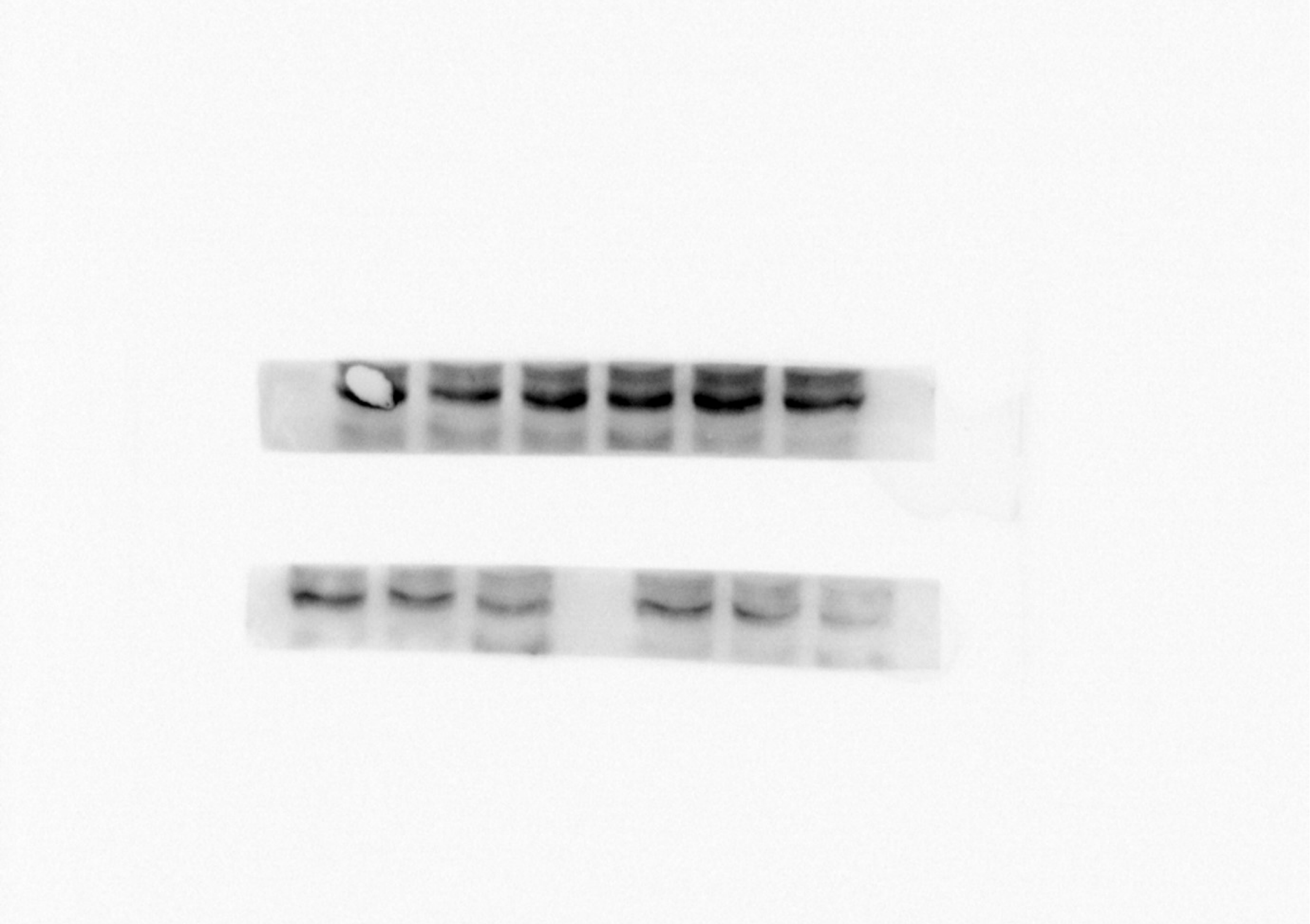

Original membrane for (48)

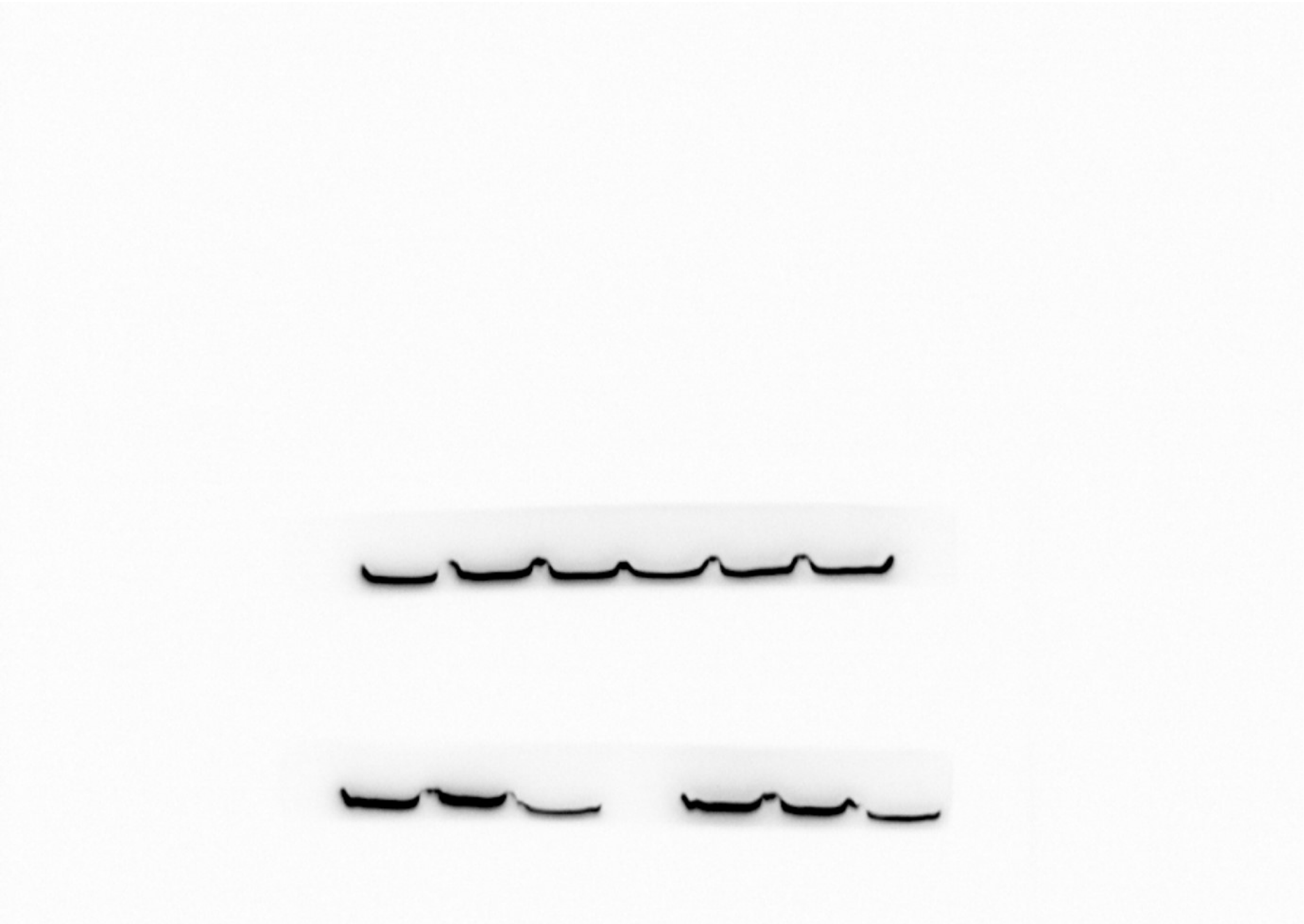

Figure 6 L、M

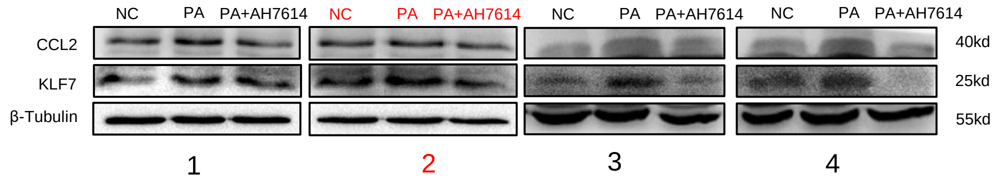
NC PA PA+AH7614 NC PA PA+AH7614 NC PA PA+AH7614 NC PA PA+AH7614
CCL2
KLF7
β-Tubulin
40kd
25kd
55kd
1
2
3
4

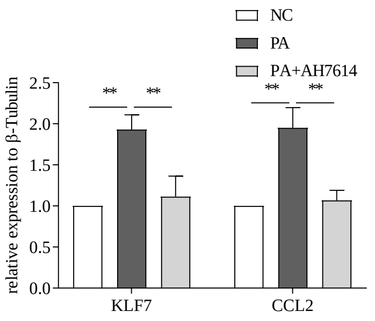
NC
PA
PA+AH7614
relative expression to β-Tubulin
2.5
2.0
1.5
1.0
0.5
0.0
**
KLF7
CCL2

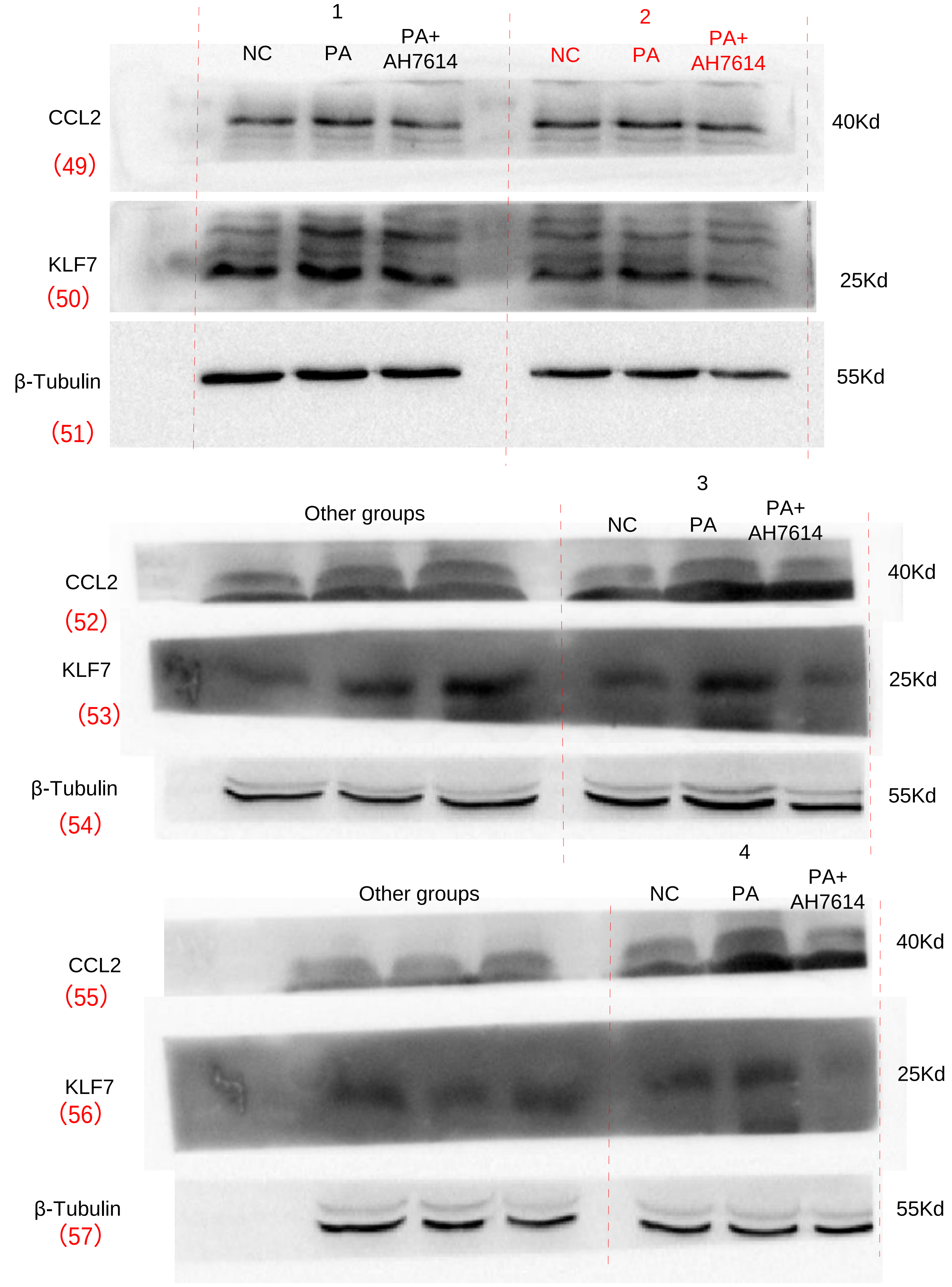
1
NC PA PA+ AH7614
2
NC PA PA+ AH7614
CCL2 (49)
KLF7 (50)
β-Tubulin (51)
40Kd
25Kd
55Kd
Other groups
3
NC PA PA+ AH7614
CCL2 (52)
KLF7 (53)
β-Tubulin (54)
40Kd
25Kd
55Kd
4
Other groups
NC PA PA+ AH7614
CCL2 (55)
KLF7 (56)
β-Tubulin (57)
40Kd
25Kd
55Kd

Original membrane for (49)

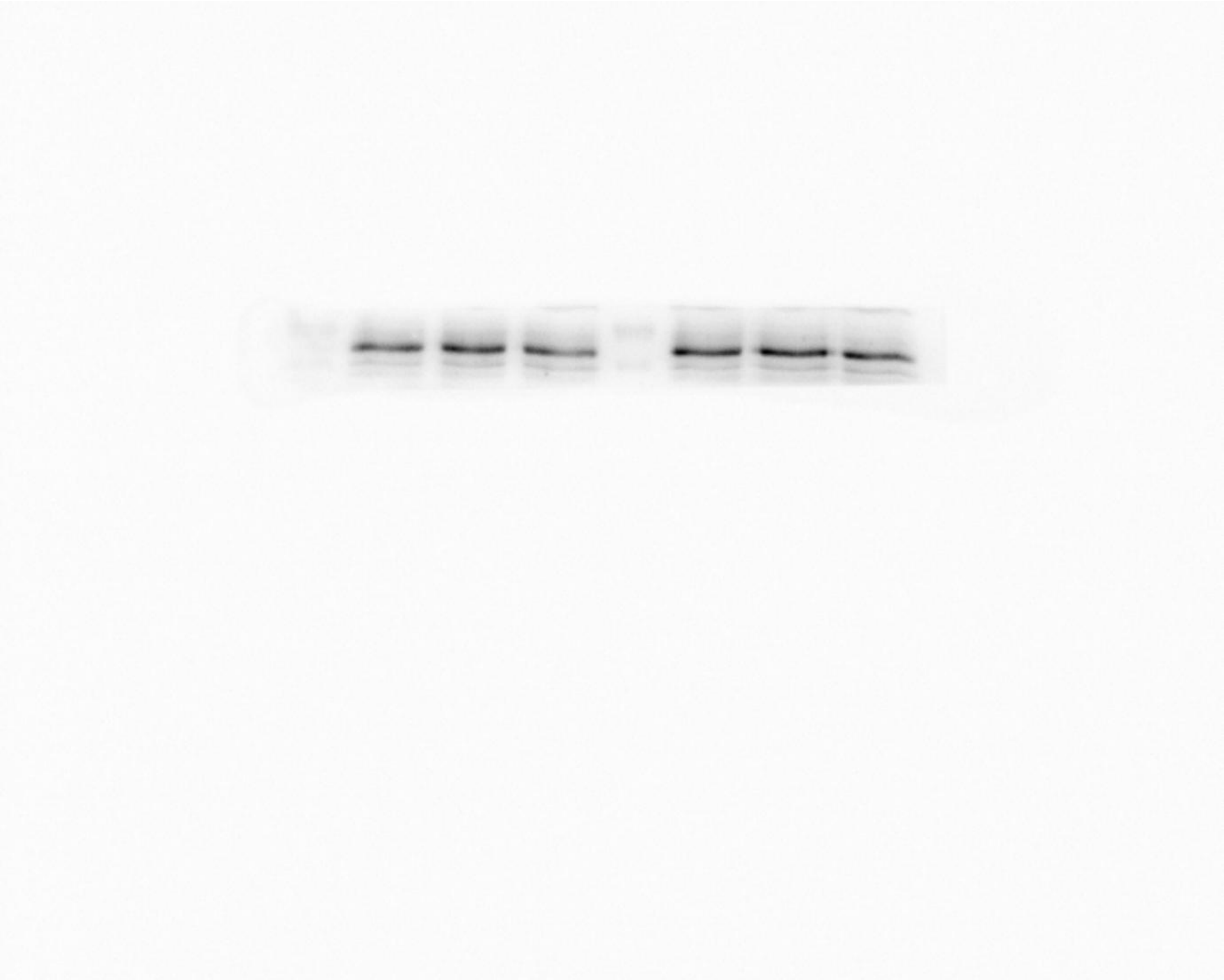

Original membrane for (50)

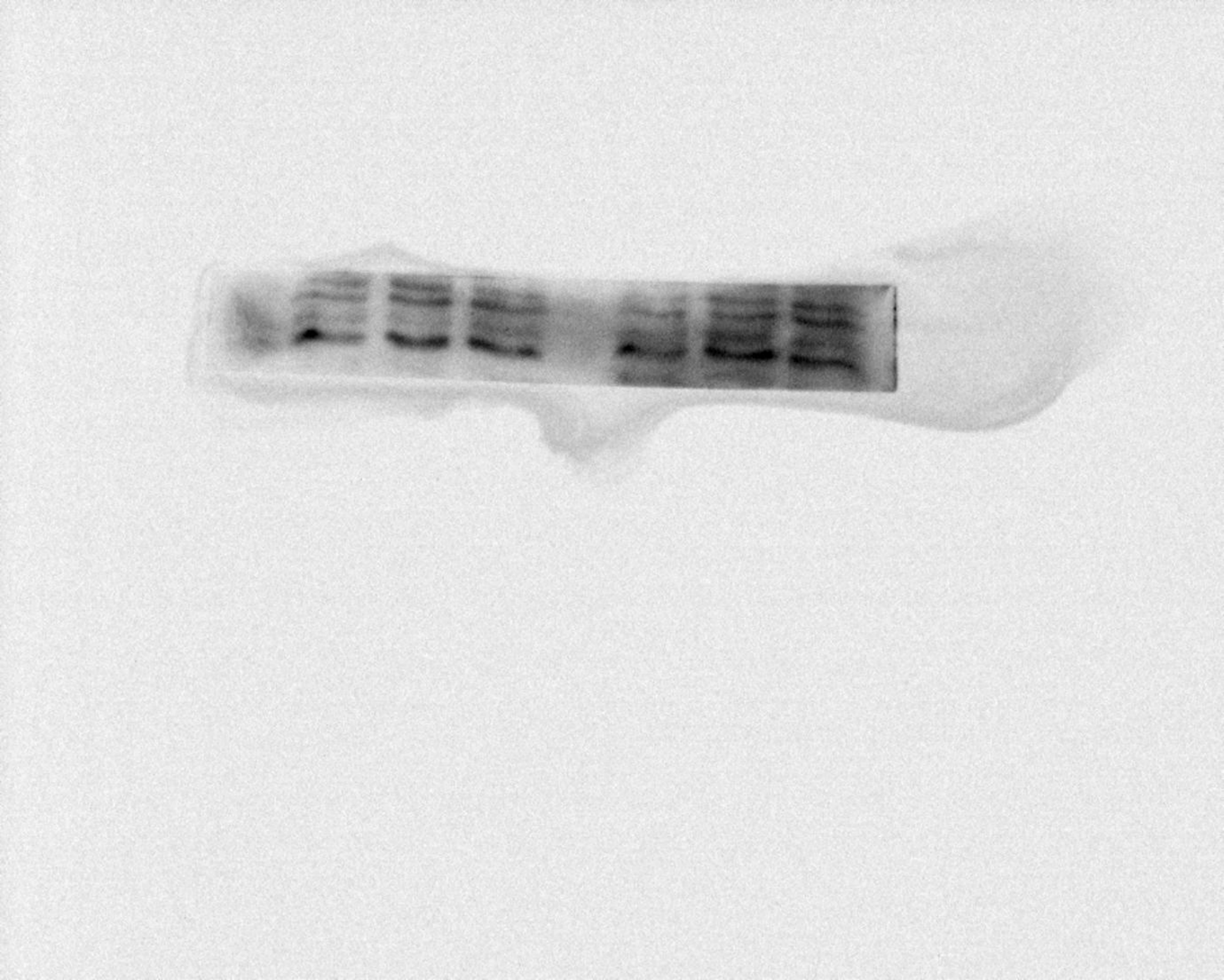

Original membrane for (51)

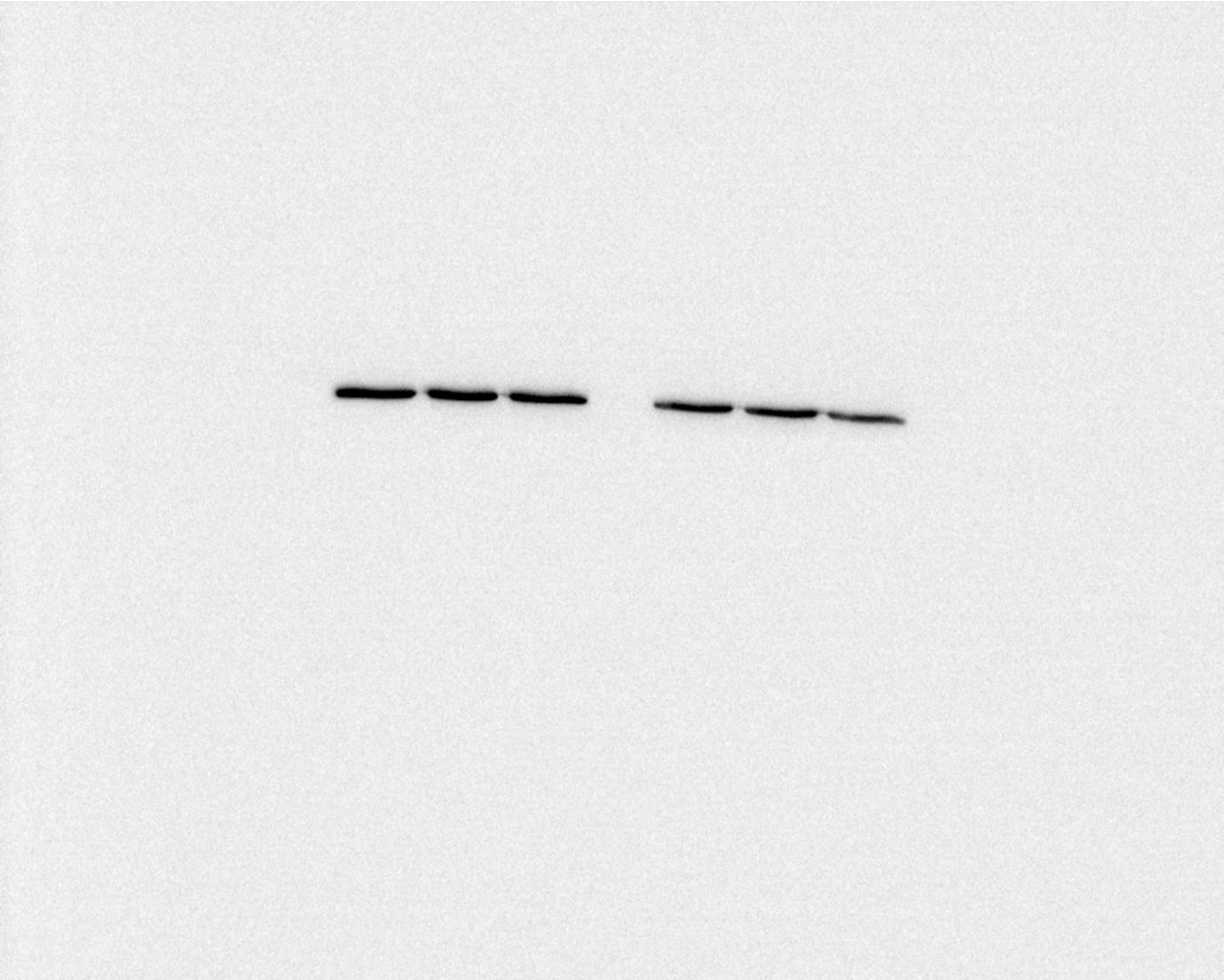

Original membrane for (52) (53)

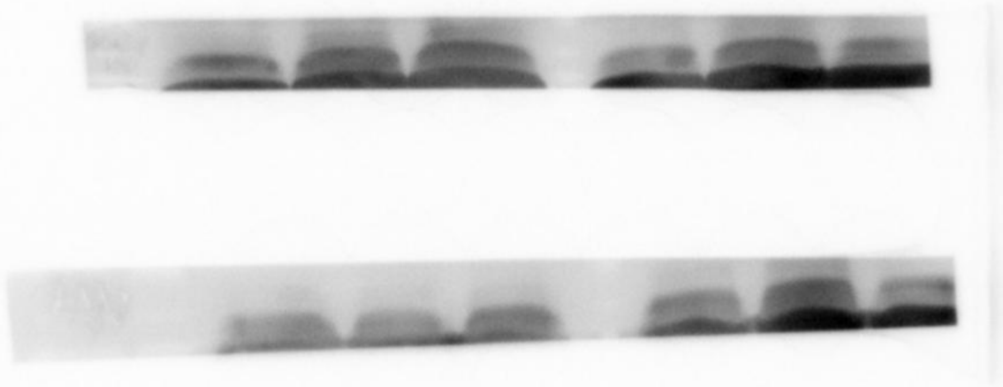

Original membrane for (54) (55)

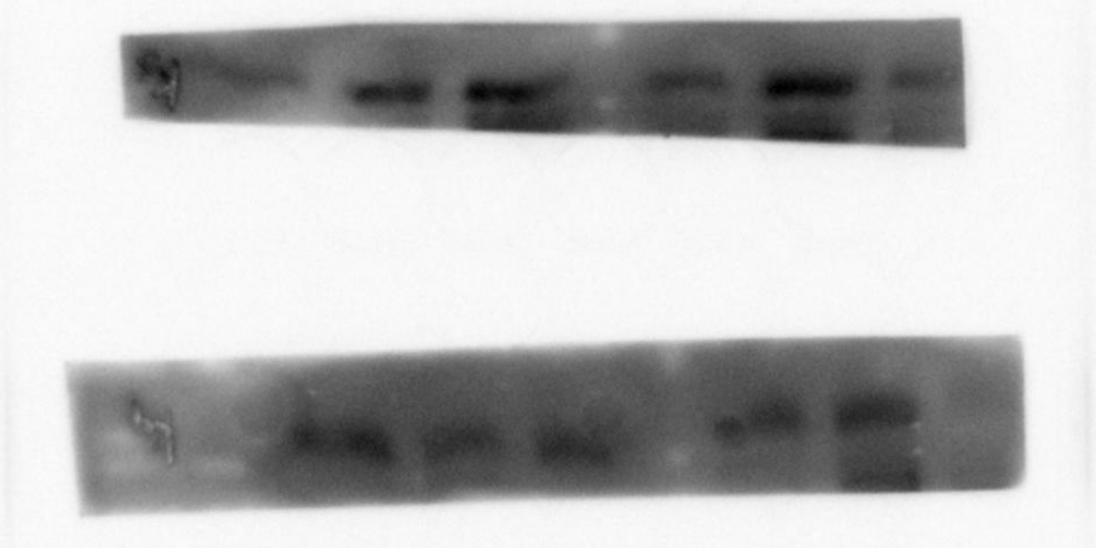

Original membrane for (56) (57)

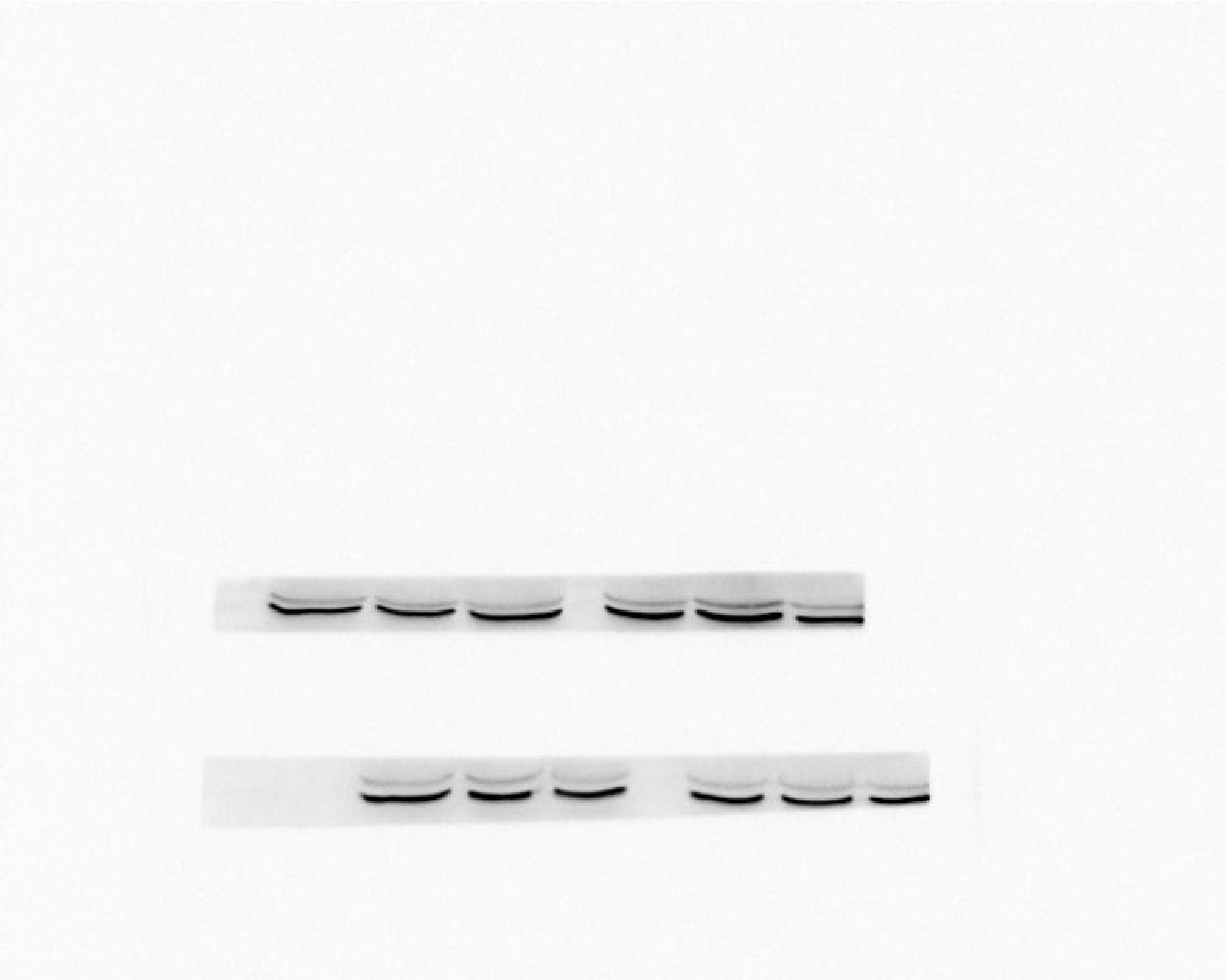

Figure 7 E、F

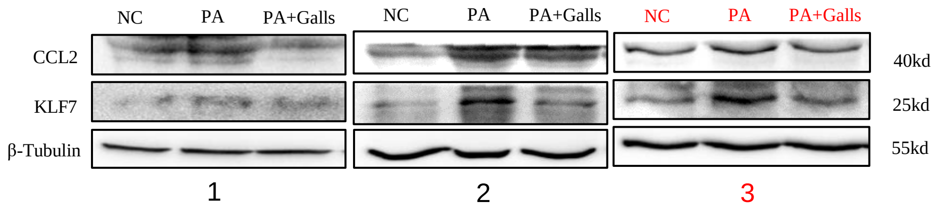
NC PA PA+Galls NC PA PA+Galls NC PA PA+Galls
CCL2
KLF7
β-Tubulin
40kd
25kd
55kd
1
2
3

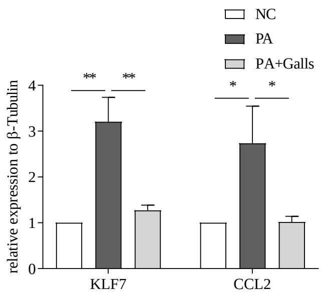
NC
PA
PA+Galls
relative expression to β-Tubulin
**
*
KLF7
CCL2

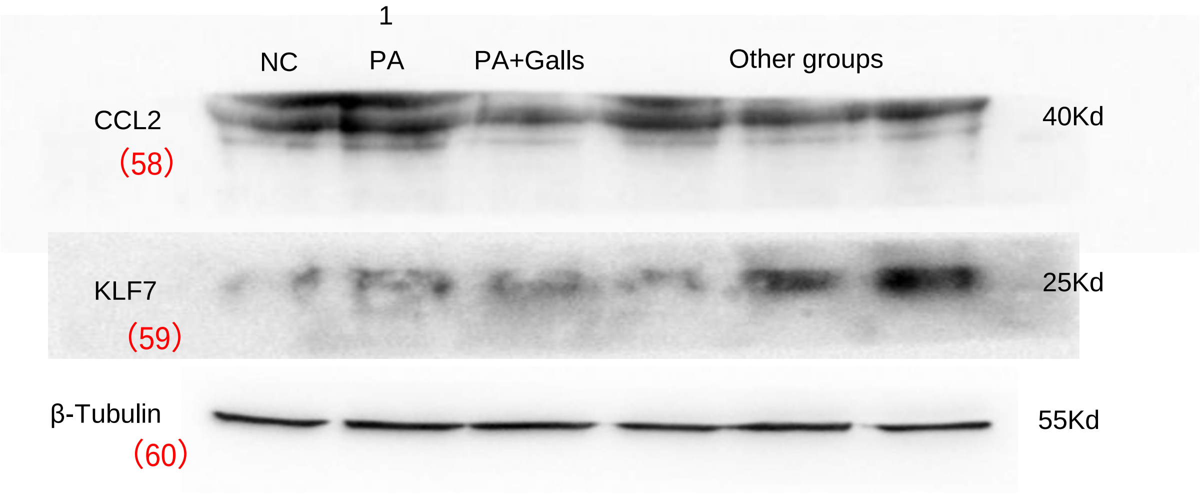
1
NC PA PA+Galls Other groups
CCL2 (58)
KLF7 (59)
β-Tubulin (60)
40Kd
25Kd
55Kd

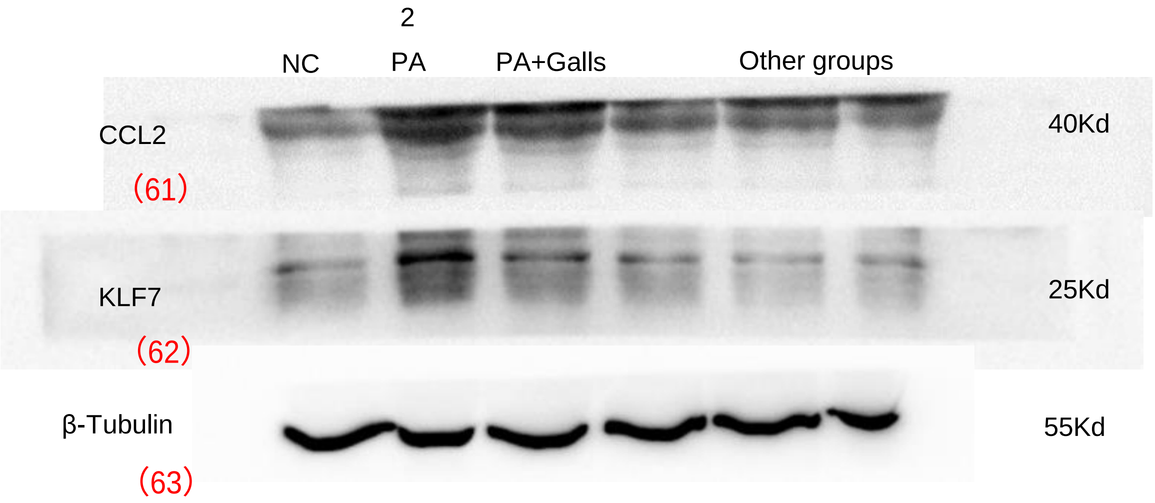
2
NC PA PA+Galls Other groups
CCL2 (61)
KLF7 (62)
β-Tubulin (63)
40Kd
25Kd
55Kd

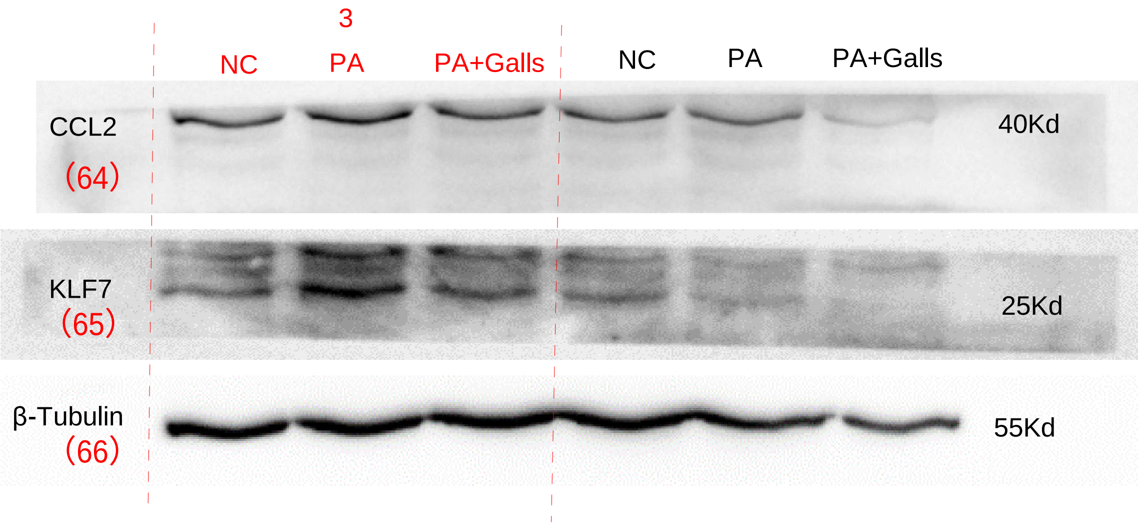
3
NC PA PA+Galls NC PA PA+Galls
CCL2 (64)
KLF7 (65)
β-Tubulin (66)
40Kd
25Kd
55Kd

Original membrane for (58)

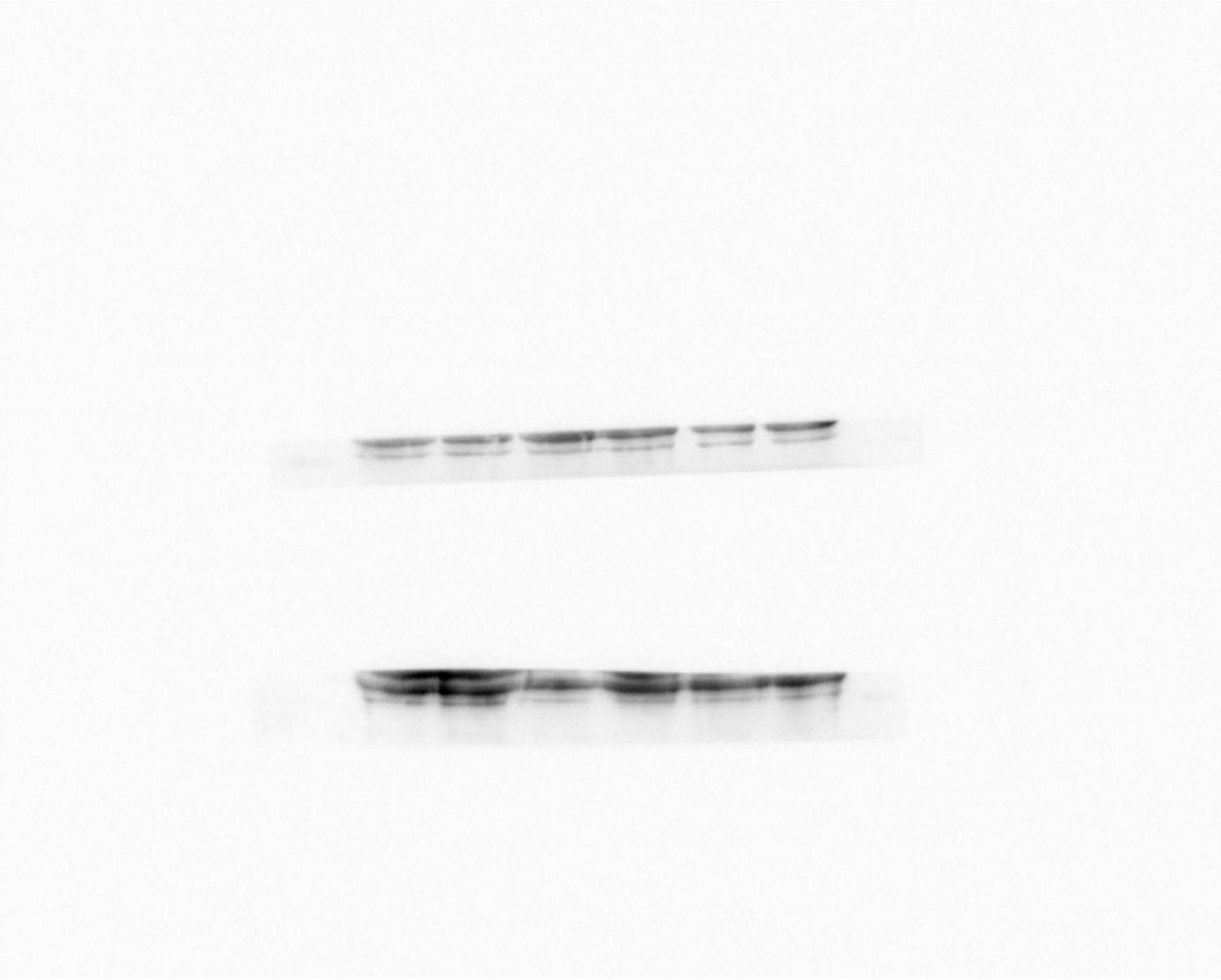

Original membrane for (59)

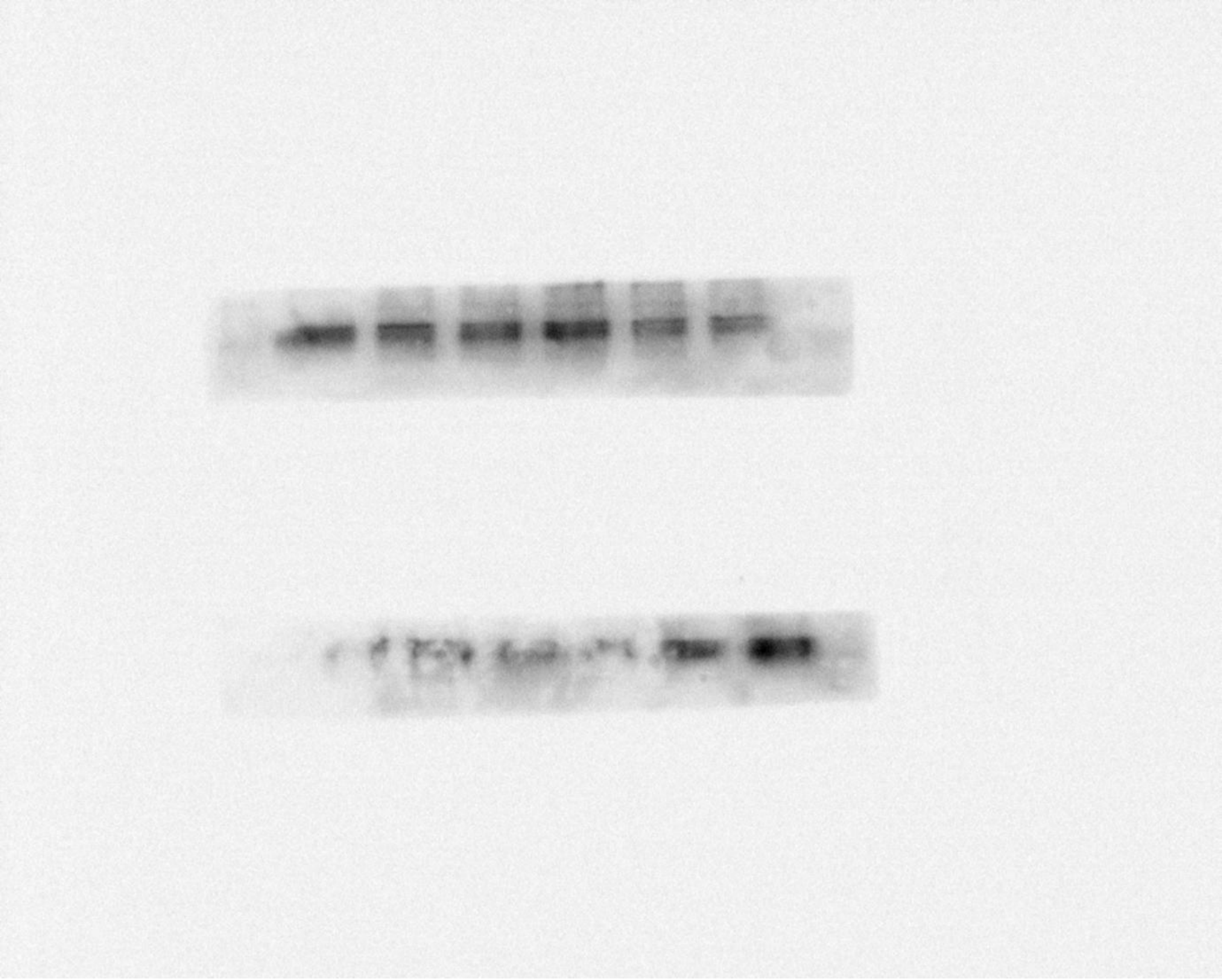

Original membrane for (60)

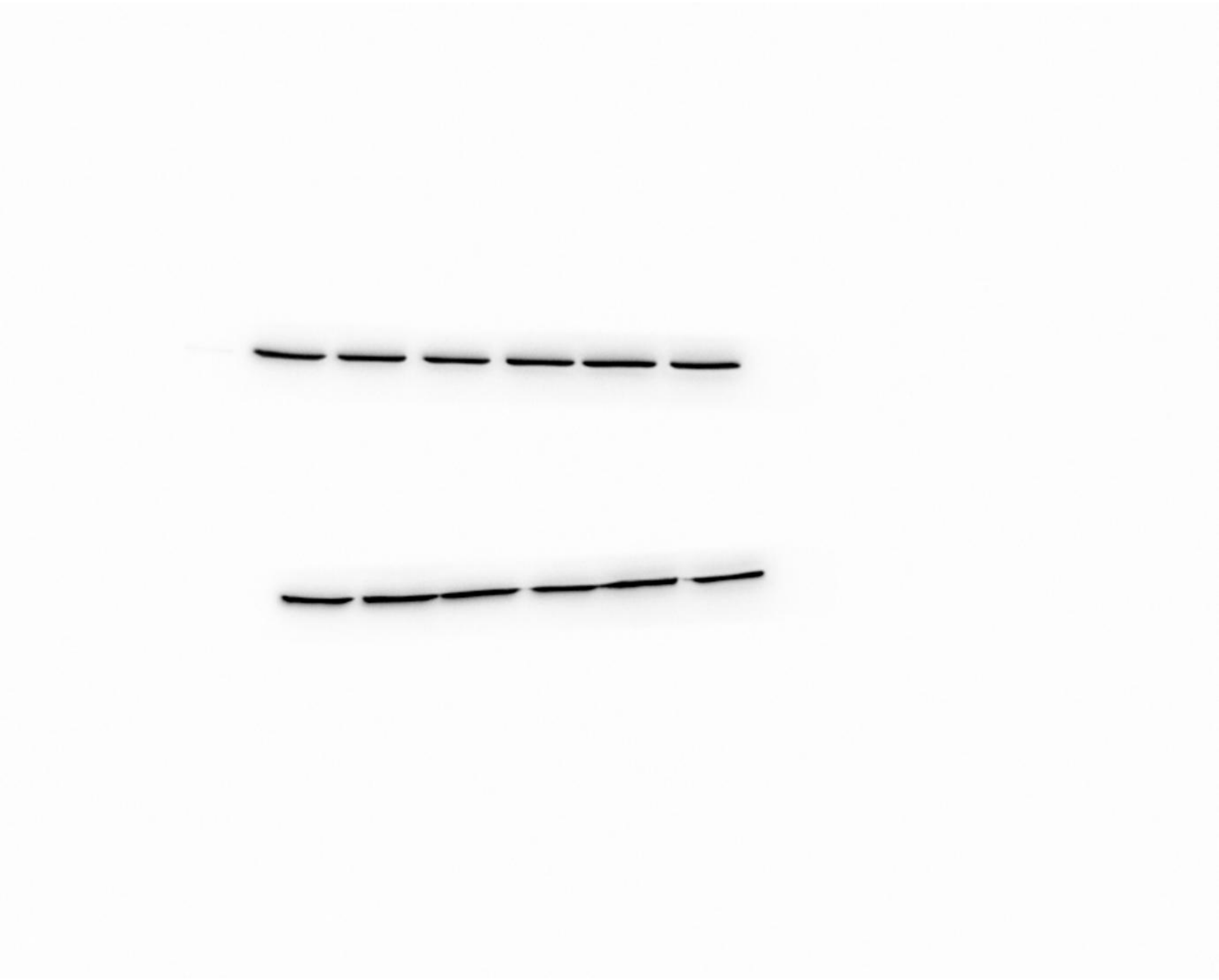

Original membrane for (61)

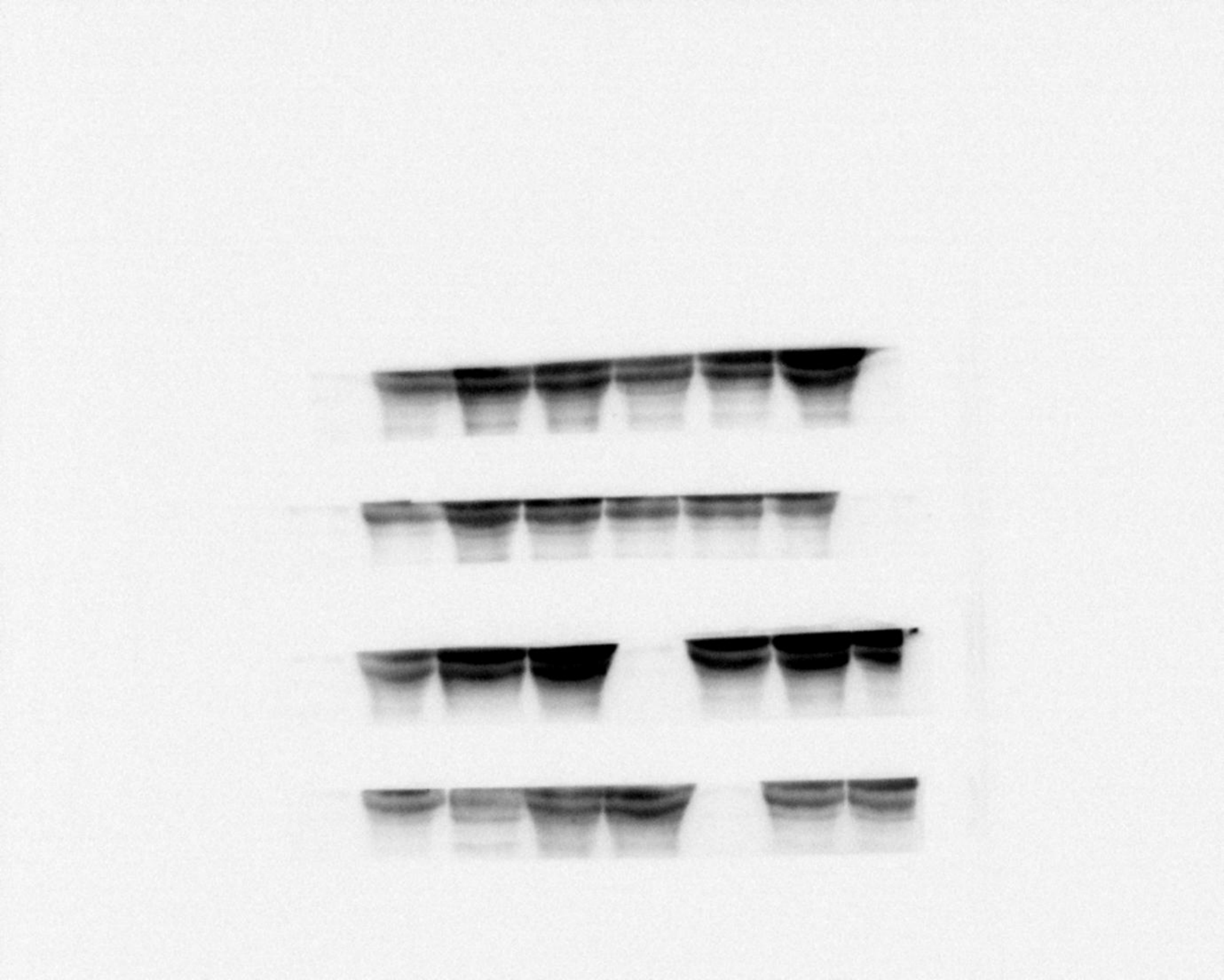

Original membrane for (62)

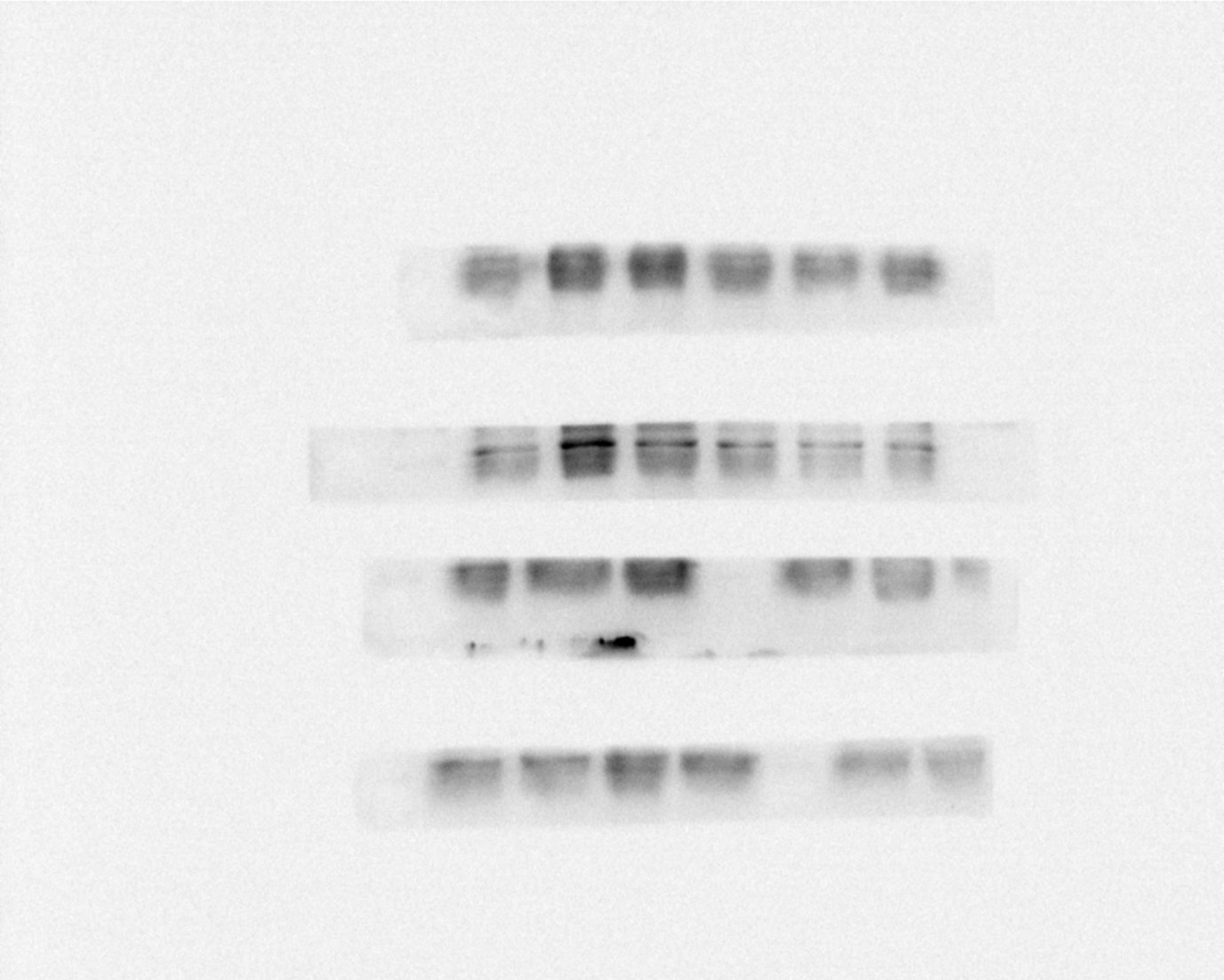

Original membrane for (63)

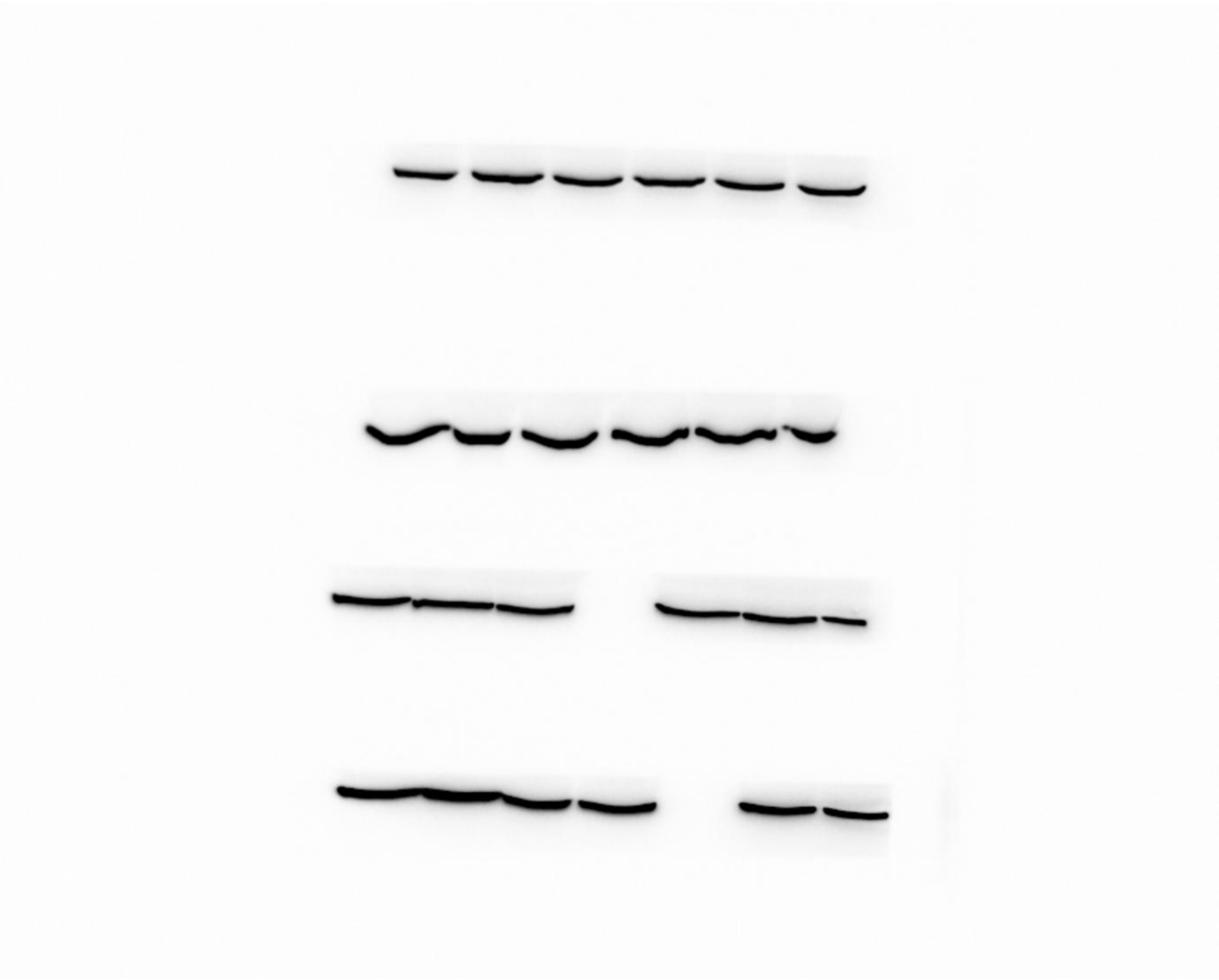

Original membrane for (64)

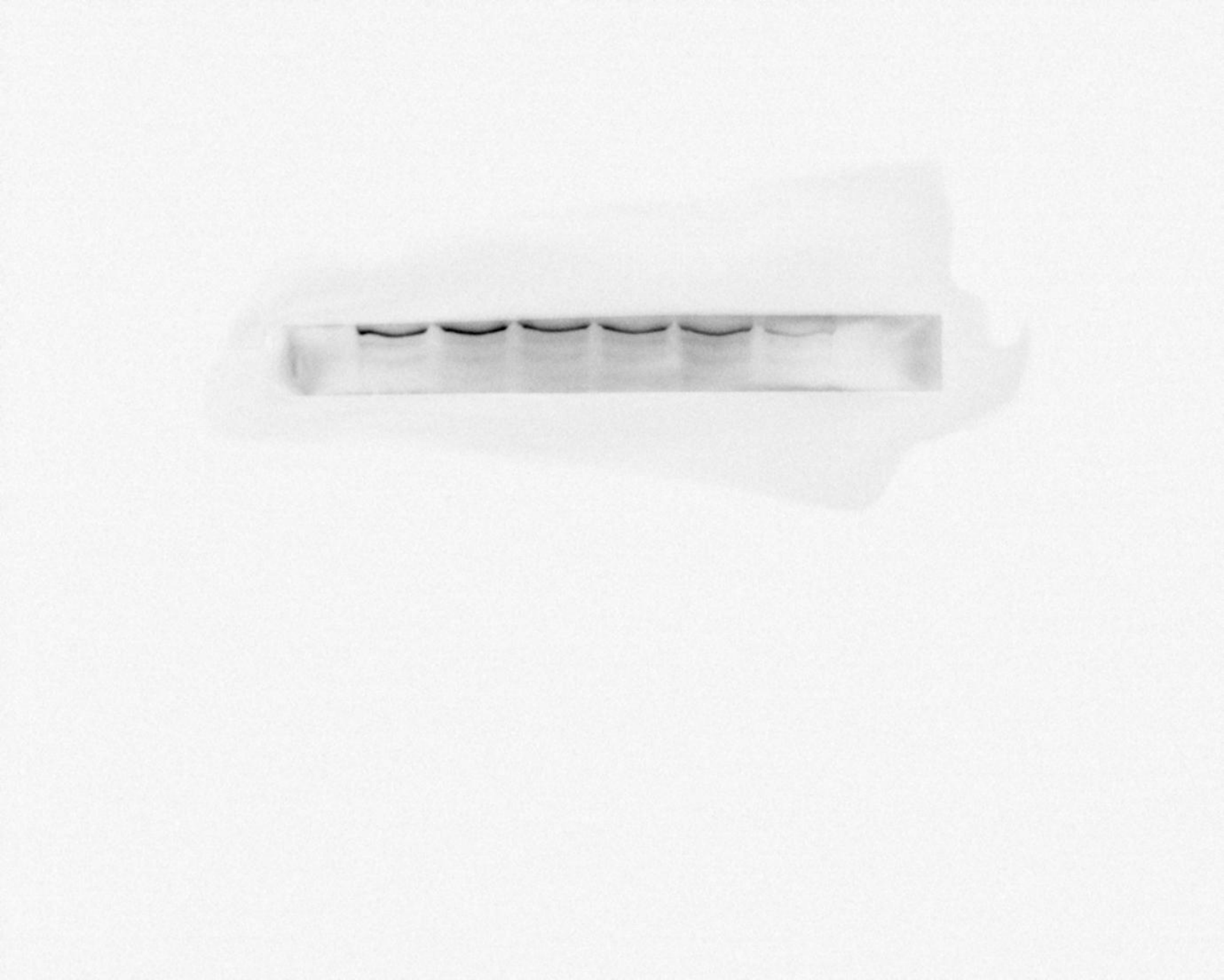

Original membrane for (65)

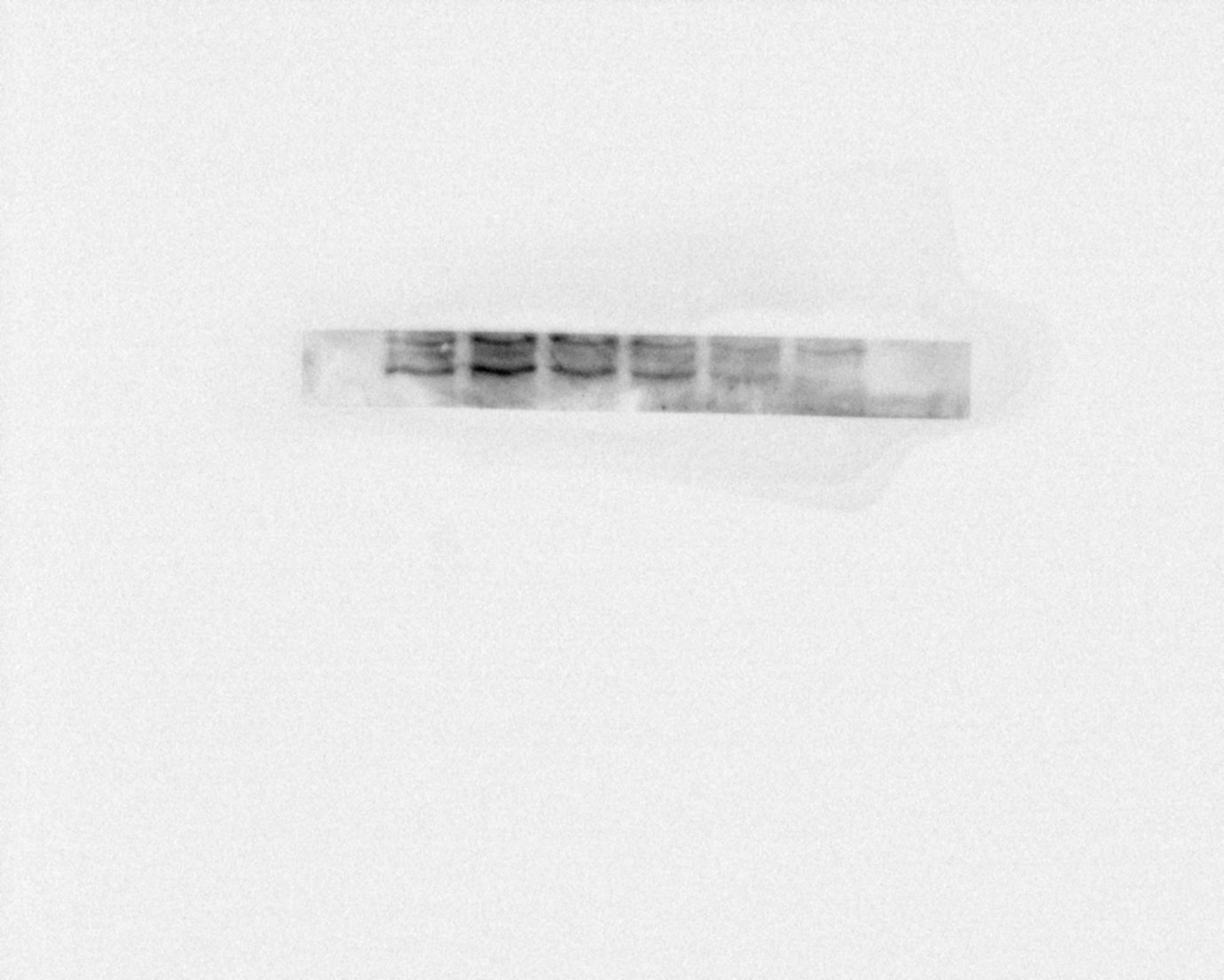

Original membrane for (66)

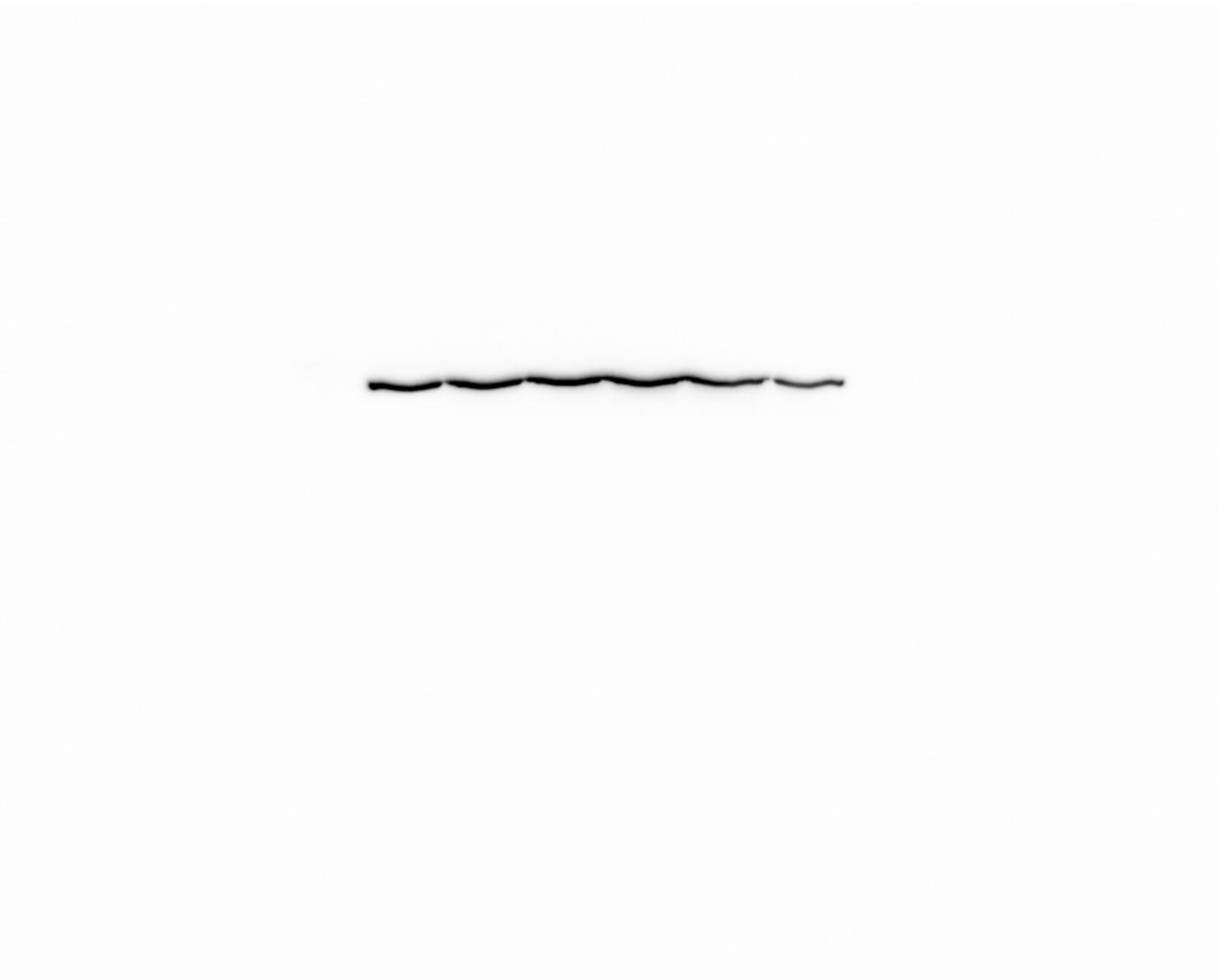

Supplement: Supplementary file 4 — Additional file 4. WB original images [file 12885_2024_11826_MOESM4_ESM.pdf]
